# A comparison of modeling approaches for static and dynamic prediction of central line-associated bloodstream infections using electronic health records (part 2): random forest models

**DOI:** 10.1186/s41512-025-00194-8

**Published:** 2025-07-21

**Authors:** Elena Albu, Shan Gao, Pieter Stijnen, Frank E Rademakers, Christel Janssens, Veerle Cossey, Yves Debaveye, Laure Wynants, Ben Van Calster

**Affiliations:** 1 Department of Development & Regeneration, KU Leuven, Leuven, Belgium; 2 Management Information Reporting Department, University Hospitals Leuven, Leuven, Belgium; 3 Faculty of Medicine, KU Leuven, Leuven, Belgium; 4 Vascular Access Specialty Team, University Hospitals Leuven, Leuven, Belgium; 5 Department of Infection Control and Prevention, University Hospitals Leuven, Leuven, Belgium; 6 Department of Cellular and Molecular Medicine, University Hospitals Leuven, Leuven, Belgium; 7 Leuven Unit for Health Technology Assessment Research (LUHTAR), KU Leuven, Leuven, Belgium; 8 School for Public Health and Primary Care, Maastricht University, Maastricht, The Netherlands

**Keywords:** Random forests, Competing risks, Survival, CLABSI, EHR, Dynamic prediction

## Abstract

**Objective:**

Prognostic outcomes related to hospital admissions typically do not suffer from censoring, and can be modeled either categorically or as time-to-event. Competing events are common but often ignored. We compared the performance of static and dynamic random forest (RF) models to predict the risk of central line-associated bloodstream infections (CLABSI) using different outcome operationalizations.

**Methods:**

We included data from 27,478 admissions to the University Hospitals Leuven, covering 30,862 catheter episodes (970 CLABSI, 1466 deaths and 28,426 discharges) to build static and dynamic RF models for binary (CLABSI vs no CLABSI), multinomial (CLABSI, discharge, death or no event), survival (time to CLABSI) and competing risks (time to CLABSI, discharge or death) outcomes to predict the 7-day CLABSI risk. Static models used information at the onset of the catheter episode, while dynamic models updated predictions daily for 30 days (landmark 0-30). We evaluated model performance across 100 train/test splits.

**Results:**

Performance of binary, multinomial and competing risks models was similar: AUROC was 0.74 for predictions at catheter onset, rose to 0.77 for predictions at landmark 5, and decreased thereafter. Survival models overestimated the risk of CLABSI (E:O ratios between 1.2 and 1.6), and had AUROCs about 0.01 lower than other models. Binary and multinomial models had lowest computation times. Models including multiple outcome events (multinomial and competing risks) display a different internal structure compared to binary and survival models, choosing different variables for early splits in trees.

**Discussion and conclusion:**

In the absence of censoring, complex modelling choices do not considerably improve the predictive performance compared to a binary model for CLABSI prediction in our studied settings. Survival models censoring the competing events at their time of occurrence should be avoided.

**Supplementary information:**

The online version contains supplementary material available at 10.1186/s41512-025-00194-8.

## Background and significance

Electronic Health Records (EHR) are commonly used to develop prediction models using statistical or machine learning (ML) methods. Many models utilizing EHR data focus on acute clinical events during hospital or ICU admissions, such as sepsis [1–4], ventilator-associated pneumonia [5] and acute kidney injury [6]. These models vary with regards to the prediction time point: static (e.g., early detection at admission) or dynamic models (predictions are updated at different times during the patient follow up). Models using EHR data are often built against surveillance event definitions. In contrast to outcomes recorded after patient discharge (such as ICD codes), the time on the patient timeline when the clinical event of interest has occurred is typically known whenever surveillance event definitions are used. This makes time-to-event models, also known as survival models, an attractive modeling choice. The outcome of interest is typically a specific event (e.g., sepsis), but competing events may preclude the occurrence of the event of interest (e.g., being discharged in good health conditions or dying of other causes). Competing events are regularly ignored during model building, which may be detrimental to the models’ predictive performance according to statistical literature [7].

A particularity of EHR data is that patients are normally followed up until discharge; therefore in-hospital outcomes based on EHR data are not subject to loss to follow up and the outcome can be modeled either categorically (i.e., as binary or multinomial) or using time-to-event approaches. While survival or competing risks models are the preferred methods when censoring is present, the absence of censoring does not render these models invalid; it only simplifies the settings in which these models operate.

Random forests (RFs) [8] are ensemble machine learning models, initially proposed for regression and classification. They have been extended for survival [9] and competing risks [10] settings, with adapted split rules for the survival outcome (survival difference between the left and right daughter nodes) or competing risks outcome (cause-specific cumulative hazard function or cumulative incidence function).

## Objective

We perform a methodological comparison of random forest models built against different outcome types (binary, multinomial, survival and competing risks) for predicting central line-associated bloodstream infection (CLABSI). CLABSI is a hospital acquired infection defined as a bloodstream infection in patients with a central line that is not related to an infection at another site. CLABSI prediction models are built primarily on EHR data and most models use a binary outcome without a fixed time horizon (CLABSI event at any time during adm [11]. Most models are static, making predictions at a fiission) or survival models not accounting for competing risks [xed moment in time (usually at catheter placement), without updating the predictions throughout the patient’s hospitalization.

The question we aim to answer is whether incorporating more information in the outcome (exact event times for survival and competing risks; additional events in multinomial and competing risks models) leads to improved performance compared to the binary model to predict the 7 day risk of CLABSI. We build both static and dynamic models and consider discharge, catheter removal and patient death as competing events that preclude the occurrence of the event of interest.

## Data and methods

### Study design and participants

Patient data are extracted from the EHR system of the University Hospitals Leuven for hospital admissions in the period January 2012–December 2013. We included patient admissions with registration of one or multiple central lines: centrally inserted central catheter (CICC), tunneled cuffed and non-cuffed central venous catheter, port catheter (totally implanted vascular access devices (TIVAD)), peripherally inserted central catheter (PICC) and dialysis catheter. The terms “central line” and “catheter” will be used interchangeably. Patients with only a dialysis catheter (and no other catheter type) were included only if they were admitted to ICU (due to the data extraction constraint). Because the neonatology department did not record catheters in the EHR system before October 2013, 260 neonates admissions have been excluded. The dataset consists of 27,478 hospital admissions after excluding neonates.

The following levels of the outcome are considered:

- **CLABSI**: any laboratory-confirmed bloodstream infection (LC-BSI) during hospital admission for a patient with central line or within 48 h after the central line removal that is not present in the first 48 h after admission, that is not a secondary infection, is not a skin contamination and is not a mucosal barrier injury LC-BSI. The CLABSI definition has been calculated retrospectively based on the extracted data following the Sciensano definition published in 2019 [12].
- **Discharge**: hospital discharge or 48 h after catheter removal, whichever happens first. According to the Sciensano definition, the patient remains at risk of CLABSI for 48 h after catheter removal.
- **Death**: either the first contact with palliative care during admission, transfer to palliative care or patient death, whichever happens first. Patients stop being closely monitored in palliative care and predictions on this ward are not actionable.

**Fig. 1:**
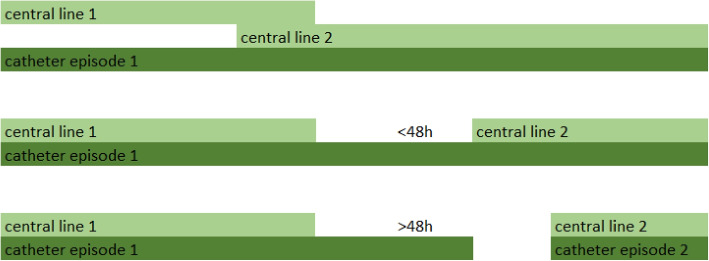
Catheter episodes

Patient admissions are split in catheter episodes. A catheter episode starts at catheter placement or at the registration of the first catheter observation during that admission (e.g., in case of admission to hospital with a long-term catheter) and ends when no catheter observation is made for 48 h or when discharge, death or CLABSI occurs. In case of multiple concomitant catheters (e.g., admission with a long-term catheter and placement of another central venous catheter during hospitalization), these are grouped in the same catheter episode if they are overlapping or if there is a time gap of less than 48 h between removal of one catheter and placement of a new one (Fig. 1).

A catheter episode is further split in landmarks (LMs). The exact time of the first catheter observation is considered landmark 0 (LM0). Subsequent landmarks are created every 24 h (LM1 is 24 h after LM0, LM2 48 h after LM0, and so on up to LM30); each LM corresponds to a catheter-day in the catheter episode. Predictions are made every 24 h. The landmark dataset contains in rows each LM and in columns the features which may have varying values on different landmarks. An excerpt of the dataset is presented in Supplementary material 1.

The dataset consists of 27,478 admissions, 30,862 catheter episodes with complete follow-up for all event types: 970 CLABSI, 1466 deaths and 28,426 discharges. There are in total 227,928 landmarks.

### Prediction horizon and outcome types

The risk of CLABSI in any of the following 7 days is predicted at each landmark. We will represent the outcome in four different ways:

- Binary: CLABSI vs. no CLABSI within 7 days.
- Multinomial: CLABSI, death, discharge and no event within 7 days.
- Survival: status 1 if CLABSI occurs and 0 (censored) if no CLABSI occurs; the event time is the time of CLABSI occurrence or the censoring time.
- Competing risks (CR): CLABSI, death and discharge with their event times.

### Features

The dataset consists of 302 baseline (onset of catheter episode) and time-varying features comprising admission and demographics, medication, laboratory test results, comorbidities, vital signs and catheter registrations. The features have been reviewed by three clinical experts (an infection preventionist, a clinical nurse specialist in vascular access and a critical care physician) and assessed as important or unimportant for CLABSI prediction. Out of the complete set of features, 21 have been selected for the model building, based on the clinical review as well as their inclusion in other CLABSI prediction studies [11]. The features included in the model and the patient characteristics at baseline are presented in Table 1. Full features descriptions are available in Supplementary material 2.

**Table 1:** Patient characteristics at baseline using the features included in the models (n = 30,862 catheter episodes); mean (sd) for continuous variables, before missing data imputation; n (%) for categorical variables. Patients can have two different catheter types simultaneously with different locations, therefore catheter type and location have been coded as binary and not categorical features with multiple categories

Feature name	Description	Statistic	Total	CLABSI	Death	Discharge
			*n *= 30,862	*n *= 970	*n *= 1466	*n* = 28,426
CVC	Catheter type CICC	0, *n*(%)	17,264 (55.9)	340 (35.1)	872 (59.5)	16,052 (56.5)
		1, *n*(%)	13,598 (44.1)	630 (64.9)	594 (40.5)	12,374 (43.5)
Port catheter	Catheter type TIVAD	0, *n*(%)	16,855 (54.6)	768 (79.2)	744 (50.8)	15,343 (54.0)
		1, *n*(%)	14,007 (45.4)	202 (20.8)	722 (49.2)	13,083 (46.0)
Tunneled CVC	Catheter type tc-CICC or t-CICC	0, *n*(%)	29,489 (95.6)	870 (89.7)	1426 (97.3)	27,193 (95.7)
		1, *n*(%)	1373 (4.4)	100 (10.3)	40 (2.7)	1233 (4.3)
PICC	Catheter type PICC	0, *n*(%)	29,579 (95.8)	941 (97.0)	1380 (94.1)	27,258 (95.9)
		1, *n*(%)	1283 (4.2)	29 (3.0)	86 (5.9)	1168 (4.1)
Jugular	Catheter location jugular	0, *n*(%)	14,104 (45.7)	581 (59.9)	592 (40.4)	12,931 (45.5)
		1, *n*(%)	16,758 (54.3)	389 (40.1)	874 (59.6)	15495 (54.5)
Subclavian	Catheter location subclavian	0, *n*(%)	18,793 (60.9)	448 (46.2)	1026 (70.0)	17,319 (60.9)
		1, *n*(%)	12,069 (39.1)	522 (53.8)	440 (30.0)	11,107 (39.1)
TPN	Total parenteral nutrition (TPN) order in previous 7 days	0, *n*(%)	28,494 (92.3)	686 (70.7)	1292 (88.1)	26,516 (93.3)
		1, *n*(%)	2368 (7.7)	284 (29.3)	174 (11.9)	1910 (6.7)
AB	Antibacterials order in previous 7 days	0, *n*(%)	13,060 (42.3)	279 (28.8)	563 (38.4)	12,218 (43.0)
		1, *n*(%)	17,802 (57.7)	691 (71.2)	903 (61.6)	16,208 (57.0)
Chemotherapy	Antineoplastic agents order in previous 7 days	0, *n*(%)	24,720 (80.1)	897 (92.5)	1410 (96.2)	22,413 (78.8)
		1, *n*(%)	6142 (19.9)	73 (7.5)	56 (3.8)	6013 (21.2)
CLABSI history	CLABSI history	0, *n*(%)	30,304 (98.2)	940 (96.9)	1434 (97.8)	27,930 (98.3)
		1, *n*(%)	558 (1.8)	30 (3.1)	32 (2.2)	496 (1.7)
Tumor history	Tumor history	0, *n*(%)	14,078 (45.6)	598 (61.6)	634 (43.2)	12,846 (45.2)
		1, *n*(%)	16,784 (54.4)	372 (38.4)	832 (56.8)	15,580 (54.8)
Temperature	Max. temperature in last 24 h [°C]	Mean (SD)	36.8 (0.8)	37.0 (0.9)	37.0 (0.9)	36.8 (0.8)
Systolic BP	Last systolic blood pressure in last 24 h [mmHg]	Mean (SD)	125.8 (22.3)	123.0 (23.5)	121.3 (25.7)	126.1 (22.1)
WBC	White blood cell count in last 24 h [10**9/L]	Mean (SD)	9.4 (8.4)	10.4 (12.1)	11.7 (13.5)	9.2 (7.7)
Lymphoma history	Lymphoma history	0, *n*(%)	29,480 (95.5)	928 (95.7)	1387 (94.6)	27,165 (95.6)
		1, *n*(%)	1382 (4.5)	42 (4.3)	79 (5.4)	1261 (4.4)
Transplant history	Transplant history	0, *n*(%)	29,355 (95.1)	904 (93.2)	1391 (94.9)	27,060 (95.2)
		1, *n*(%)	1507 (4.9)	66 (6.8)	75 (5.1)	1366 (4.8)
Other infection than BSI	Other infection than BSI in previous 17 days	0, *n*(%)	27768 (90.0)	847 (87.3)	1252 (85.4)	25,669 (90.3)
		1, *n*(%)	3094 (10.0)	123 (12.7)	214 (14.6)	2757 (9.7)
CRP	C-reactive protein unit in last 24 h [mg/L]	Mean (SD)	48.7 (71.9)	59.3 (84.0)	95.3 (96.6)	45.1 (68.1)
Admission source: home	Admission source: home	0, *n*(%)	3728 (12.3)	240 (24.9)	344 (23.7)	3144 (11.2)
		1, *n*(%)	26,637 (87.7)	725 (75.1)	1107 (76.3)	24,805 (88.8)
MV	Mechanical ventilation in last 24 h	0, *n*(%)	29,352 (95.1)	872 (89.9)	1253 (85.5)	27,227 (95.8)
		1, *n*(%)	1510 (4.9)	98 (10.1)	213 (14.5)	1199 (4.2)
ICU	Patient currently in ICU (intensive care unit) ward	0, *n*(%)	25,807 (83.6)	683 (70.4)	973 (66.4)	24,151 (85.0)
		1, *n*(%)	5055 (16.4)	287 (29.6)	493 (33.6)	4275 (15.0)
Days to event	Days to event	Mean (SD)	7.3 (9.4)	13.0 (14.6)	9.8 (13.6)	7.0 (8.8)

### Train/test split

One hundred random train/test splits are generated on the landmark dataset, keeping two thirds of the hospital admissions for training and one third for test, so that a full admission (with all its catheter episodes and all its landmarks) falls entirely in train set or entirely in the test set. Baseline datasets have then been generated by filtering the dynamic datasets for LM0. Missing data have been imputed using a combination of mean/mode imputation, normal value imputation and the missForestPredict algorithm [13] (Supplementary material 4). The average number and proportion of events in the baseline and dynamic training and test sets are presented in Table 2. Cumulative incidence functions (CIF) for all events are presented in Supplementary material 3.

**Table 2:** Average number and average proportion of events in train and test sets; for baseline datasets the averaging is done over all catheter episodes; for dynamic datasets the averaging is done over all landmarks

Baseline/dynamic	Horizon	Train/test	CLABSI, *n *(%)	Death, *n *(%)	Discharge, *n* (%)	No event until horizon, *n *(%)
Baseline	Any	Train	646 (3.1%)	977 (4.7%)	18947 (92.1%)	0 (0%) by definition
Baseline	Any	Test	324 (3.1%)	489 (4.8%)	9479 (92.1%)	0 (0%) by definition
Baseline	7 days	Train	269 (1.3%)	558 (2.7%)	13039 (63.4%)	6704 (32.6%)
Baseline	7 days	Test	135 (1.3%)	279 (2.7%)	6522 (63.4%)	3356 (32.6%)
Dynamic	Any	Train	8700 (5.4%)	10050 (6.2%)	143264 (88.4%)	0 (0%) by definition
Dynamic	Any	Test	4376 (5.4%)	5078 (6.3%)	71716 (88.4%)	0 (0%) by definition
Dynamic	7 days	Train	1152 (0.7%)	1905 (1.2%)	48327 (29.8%)	110631 (68.3%)
Dynamic	7 days	Test	584 (0.7%)	945 (1.2%)	24176 (29.8%)	55464 (68.3%)

### Static and dynamic model building using random forests

On each imputed baseline and dynamic training set, RF models are trained for the different outcome types (binary, multinomial, survival and competing risks). Baseline or static models are built on the baseline datasets (only LM0 of each catheter episode) and dynamic models are built on the landmark dataset. Static models consider each catheter episode as an independent observation, while 8.2% of the admissions have more than one catheter episode. The dynamic models are built considering each landmark in each catheter episode an independent observation; the landmark number is included in the dynamic model, allowing the dynamic models to account for time effects as well as interactions with time. For static and dynamic model tuning, all catheter episodes belonging to an admission are assigned to either the training or validation data sample.

Random forest models are built using the randomforestSRC package [14]. Each tree is built on an “in-bag” (a sample of the training set) and evaluated on an “out-of-bag” (the remaining observations in the train set that are not in-bag) for hyperparameter tuning. The in-bags are created by sampling complete admissions with all their catheter episodes, so that an admission falls completely in-bag or completely out-of-bag. Sampling is done with replacement (bootstraps of size equal to the number of admissions) for static models and without replacement (subsamples with tuned subsample size between 30% and 80% of the admissions) for dynamic models; we consider that when data are large enough [15] we can build the trees on subsamples from the data; because of the limited number of events in the baseline data, we consider bootstraps a more appropriate strategy. The number of trees is fixed to 1000 trees, considering this as large enough and unnecessary to tune [15]. We thus create 1000 in-bags for each of the 100 training sets. Because randomforestSRC package imposes the limitation that all in-bags for the 1000 trees must have the same size and sampling by admission id will not result in equal in-bag sizes as not all admissions have an equal numbers of catheter episodes (and landmarks for the dynamic models), we further adjusted the in-bags to the minimum size (minsize) of all the 1000 inbags for all trees by randomly sampling out some catheter episodes and/or landmarks, which will consequently fall out-of-bag. The resulting subsample size for in-bags for dynamic models ranges between 43724 for 30% subsample size and 119,865 for 80% subsample size. This procedure had negligible effects on model performance (Supplementary material 5).

**Fig. 2:**
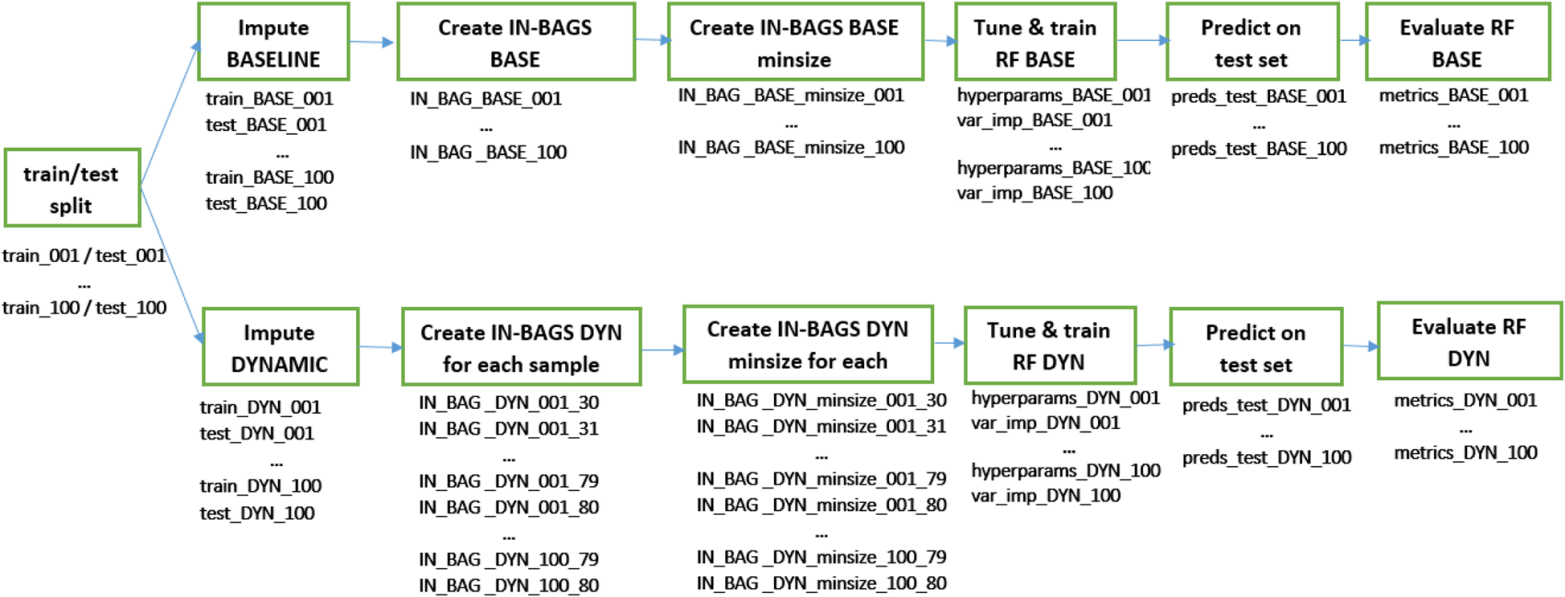
Model building pipeline

The tuned hyperparameters are the number of variables selected at each split (mtry) and the minimum size of a terminal node (nodesize) for both static and dynamic models. Additionally for dynamic models, the subsample size is tuned. Model tuning is based on the out-of-bag binary logloss for next 7 days prediction to allow all models to perform at their best for the outcome of interest. Model-based optimization tuning is performed using the mlrMBO R package [16]. (details in the Supplementary material 6). The best hyperparameters are chosen and a final RF model is built using these hyperparameters. The hyperparameter values used to build the final model, as well as a variable importance measure (the minimum depth of the maximal subtree [17]) are saved. The runtimes for model tuning, final model building and prediction on the test set are logged. The pipeline is schematically presented in Fig. 2.

Survival and competing risks models are unfeasible to train on a large dataset when using all event times. We have manipulated the event times for all events (CLABSI, death, and discharge) to speed up computations by discretizing the event times and applying administrative censoring [18]:

- **Discretized event times**: the split statistics [19] are calculated for all event times present in the dataset which proves computationally inefficient. To considerably speed up computations, event times have been discretized using ceiling(t), e.g., an event happening at day 6.1 is considered to have happened at time 7. This approach shifts events into the future rather than the past to prevent time leaks. For instance, rounding 6.1 down to 6 would imply having knowledge at day 6 of an event that was only observed later.
- **Administrative censoring**: we have applied artificial censoring as suggested by Van Houwelingen and Putter [18] by considering catheter episodes with events after a specific time t as censored. Two options are used for administrative censoring: (1) at day 7 since landmark time (the time of interest for the predictions) or (2) at day 30 since landmark time (92% of the events happen in the first 30 days after the start of the catheter episode; we consider it close to no administrative censoring).

Additional considerations have been implemented for survival and competing risks models:

- **Censoring (for survival models)**: for survival models we use two types of censoring for the competing events (death and discharge): (1) censoring at event time, e.g.: a discharge happening at day 5 is censored at time 5 (surv7 d and surv30 d models in Table 3); (2) censoring at day 7 since landmark time all competing events that happened before day 7 since landmark time, e.g., a discharge happening at time 5 is censored at time 7, keeping in the risk set catheter episodes with competing events until our time of interest, as done in Fine-Gray models [20] (surv7 d_cens7 and surv30 d_cens7 in Table 3). Alternatively, competing events could be censored beyond the time of interest, e.g., at day 30. However, we limit the analysis strictly up to the time of interest.
- **Split rule (for CR models)**: the two split rules available for competing risks models [19] are compared: (1) “logrank”, based on the difference in cause-specific hazard function and advised when the interest lies in a specific event and (2) “logrankCR” based on the difference in event-specific cumulative incidence and advised for prediction settings [19].
- **Cause (for CR models)**: when splitting a node in a competing risks model, the split-statistics weight by default all events equally. We compare: (1) the default equal weighting of events with (2) custom weighting (specified in parameter “cause”) using weight 1 for CLABSI and 0 for the other events (death and discharge outcome levels will not be used in determining tree splits).cm

**Table 3:** Models

Model name	Outcome type	Splitrule	Other hyperparameters/options	Static model	Dynamic model
bin	Binary	gini		Yes	Yes
multinom	Multinomial	gini		Yes	Yes
surv7 d	Survival	logrank	Administrative censoring at day 7; Censoring death and discharge at event time	Yes	Yes
surv7 d_cens7	Survival	logrank	Administrative censoring at day 7; Censoring death and discharge at time 7	Yes	Yes
surv30 d	Survival	logrank	Administrative censoring at day 30; Censoring death and discharge at event time	Yes	No
surv30 d_cens7	Survival	logrank	Administrative censoring at day 30; Censoring death and discharge at time 7	Yes	No
CR7 d_LRCR_c_1	Competing risks	logrankCR	cause = 1 (CLABSI); Administrative censoring at day 7	Yes	Yes
CR7 d_LR_c_1	Competing risks	logrank	cause = 1 (CLABSI); Administrative censoring at day 7	Yes	Yes
CR7 d_LRCR_c_all	Competing risks	logrankCR	cause = default (all events have equal weights); Administrative censoring at day 7	Yes	Yes
CR7 d_LR_c_all	Competing risks	logrank	cause = default (all events have equal weights); Administrative censoring at day 7	Yes	Yes
CR30 d_LRCR_c_1	Competing risks	logrankCR	cause = 1 (CLABSI); Administrative censoring at day 30	Yes	No
CR30 d_LR_c_1	Competing risks	logrank	cause = 1 (CLABSI); Administrative censoring at day 30	Yes	No
CR30 d_LRCR_c_all	Competing risks	logrankCR	cause = default (all events have equal weights); Administrative censoring at day 30	Yes	No
CR30 d_LR_c_all	Competing risks	logrank	cause = default (all events have equal weights); Administrative censoring at day 30	Yes	No

The overview of all models is presented in Table 3. We compare 14 static models and 8 dynamic models. We chose not to run the dynamic models with administrative censoring at day 30 because of increased computation time.

Models tuning, building and predictions have been run on a high-performance computing cluster using 36 cores and 128 GB RAM. R version 4.2.1 and randomForestSRC 3.2.2 have been used to build the models.

### Model evaluation

Predictions are made on the test sets for each of the model types. We retain the predicted risks of developing CLABSI in any of the next 7 days. We assume CLABSI risk within 7 days is the only outcome of clinical interest and the users of the model are not interested in predictions with other prediction horizons, nor are they interested in prediction of additional events (death or discharge). Survival models yield as predictions the survival probabilities for all time horizons with events present in the training dataset. For instance, considering the discretized event times and administrative censoring at day 30, predictions will cover each time point from day 1 to day 30, if at least an event occurred at each day.. We retain only the predictions for the time horizon of interest: $1 - p(survival\ up\ to\ day\ 7)$. Competing risks models predict the cumulative incidence function conditional on predictors and we retain the predicted CIF of CLABSI at day 7.

Following metrics are evaluated on each test set: AUPRC (area under the precision recall curve), AUROC (Area Under the ROC curve), BSS (Brier Skill Score, the Brier Score divided by a reference Brier Score of a “no skill learner” that predicts the class prevalence), E:O ratio (the mean of predicted risks divided by the mean of observed binary events), calibration slope (calculated by regressing the true binary outcome on the logit of the predicted risks [21]) and ECI (Estimated Calibration Index; the mean squared difference between the predicted probabilities and the predicted probabilities obtained with a loess fit of the observed outcome on the predicted risks, multiplied by 100 [22]). For dynamic models, time-dependent metrics (metrics calculated at each LM) are presented. The median and interquartile range (IQR) for each metric over the 100 test sets are compared.

### Code availability

The code used for model tuning, prediction and model evaluation is available at: https://github.com/sibipx/CLABSI_compare_RFSRC_models.

## Results

### Predictive performance for static models

**Fig. 3:**
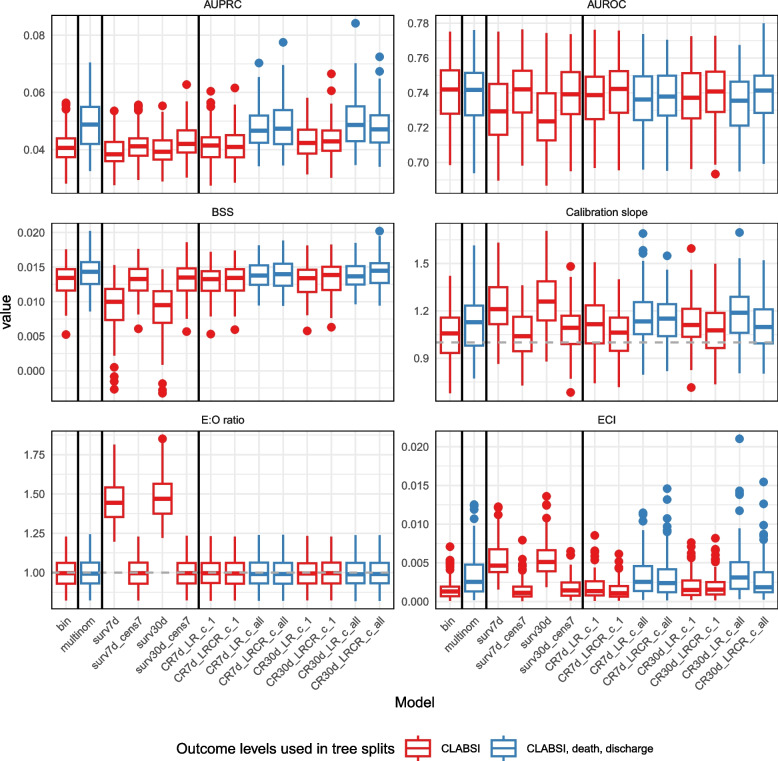
Prediction performance for static models. From left to right, separated by vertical lines: binary outcome model, multinomial outcome model, survival models, competing risk models. Models that consider all outcome classes to determine splits are displayed in blue, models that only consider CLABSI to determine splits are displayed in red

Figure 3 and Supplementary materials 7 and 10 show the performance of the static models. Survival models with competing events censored at their occurrence time (surv7 d and surv30 d) show overestimated predicted risks (median E:O ratio of 1.44 and 1.47 respectively, compared to values between 0.99 and 1.00 for the other models) and poorer discrimination (median AUROC 0.729 for surv7 d and 0.724 for surv30 d, compared to AUROC between 0.735 and 0.742 for the other models). However, if individuals experiencing competing risks are kept in the risk set by censoring the event at the time of interest for predictions (surv7 d_cens7 and surv30 d_cens7 models), the performance becomes comparable to other models: median E:O ratio of 1.00 and AUROC of 0.742 and 0.739 respectively.

**Fig. 4:**
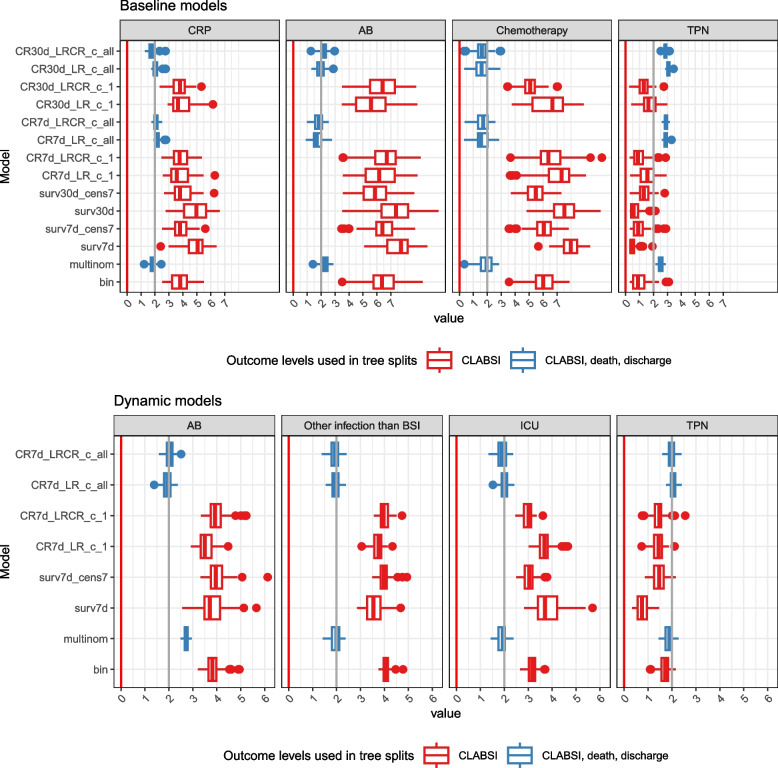
Feature minimal depth for static (baseline) and dynamic models. Includes only a subset of “important” features for which the median minimal depth is less than 2 in at least one model type. Lower minimal depths indicate more important variables. The minimal depth for all features is included in Supplementary material 7. Models that consider all outcome classes to determine splits are in blue, models that only consider CLABSI to determine splits are in red

Models using all outcome levels (multinomial, CR7 d_LRCR_c_all, CR7 d_LR_c_all, CR30 d_LRCR_c_all, CR30 d_LR_c_all) show slightly higher AUPRC, despite slight miscalibration visible in the ECI, but with no noticeable difference in AUROC. These models venture into predicting higher risks than the other models and these larger predictions lead to miscalibration (Supplementary material 7 and 10) but better discrimination. Despite small differences in performance metrics, the internal structure of these models is different. Figure 4 presents the minimal depth of the maximal subtree [17], which is the depth in a tree on which the first split is made on a variable $v$, averaged over all trees in the forest. The lowest possible value is 0 (root node split). Chemotherapy, antibiotics and CRP features are selected at first splits for models using all outcome levels, while binary and survival models favor TPN at early splits. These models also differ in hyperparameter values and predicted risk distributions (Supplementary material 7).

The choice for administrative censoring for survival and CR model (day 7 or day 30) or the splitrule used by CR models (logrank or logrankCR) did not yield any visible difference.

### Predictive performance for dynamic models

**Fig. 5:**
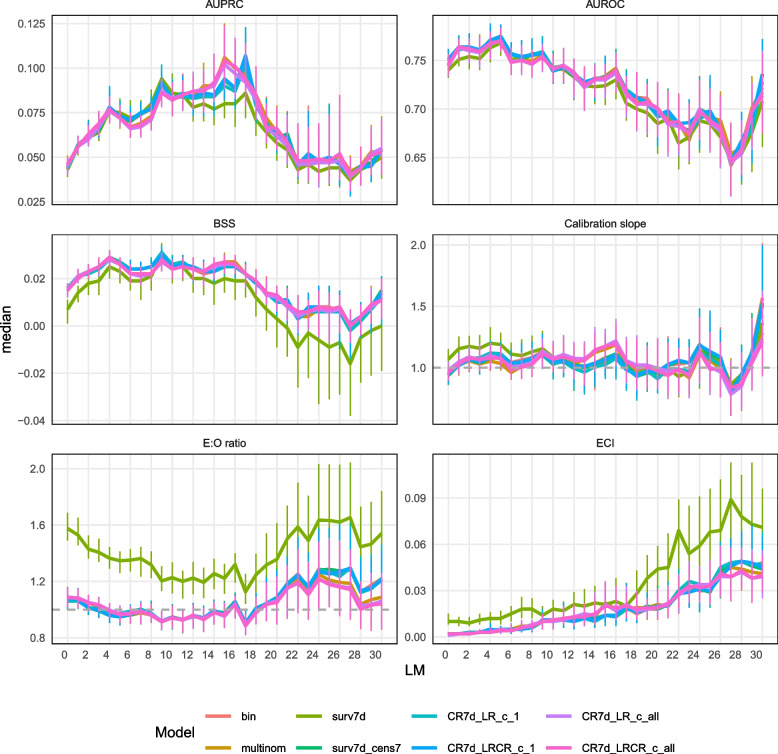
Prediction performance for dynamic models–time dependent metrics. The median value of each metric is plotted over time (landmark) and the vertical bars indicate the IQR

Figure 5 and Supplementary materials 8 and 11 show the dynamic models’ performance. All models reach their maximum AUROC at LM5. Similar to static models, the survival model with competing events censored at their occurrence time (surv7 d) shows overestimated predicted risks (median E:O ratio of 1.58 at LM0 and 1.35 at LM5, compared to values between 1.06 and 1.09 at LM0 and between 0.95 and 0.97 at LM5 for the other models) and slightly poorer discrimination (median AUROC 0.740 at LM0 and 0.768 at LM5, compared to AUROC between 0.744 and 0.752 at LM 0 and 0.770 and 0.775 at LM 5 for the other models). Censoring the competing event at the time of interest for predictions instead of at its event time (surv7 d_cens7 model) corrects this loss of calibration and discrimination. The models using multiple outcome levels choose at the first splits in the trees antibiotics, other infection than BSI and ICU (Fig. 4), while the other models favor TPN at early splits. They also differ in tuned hyperparameter values but do not exhibit an obvious difference in predicted risk distribution (Supplementary material 8) or in performance metrics. Dynamic models perform better than static models at LM0 in terms of discrimination (AUROC) and BSS, but worse in terms of calibration (Supplementary material 7).

### Computational speed

**Fig. 6:**
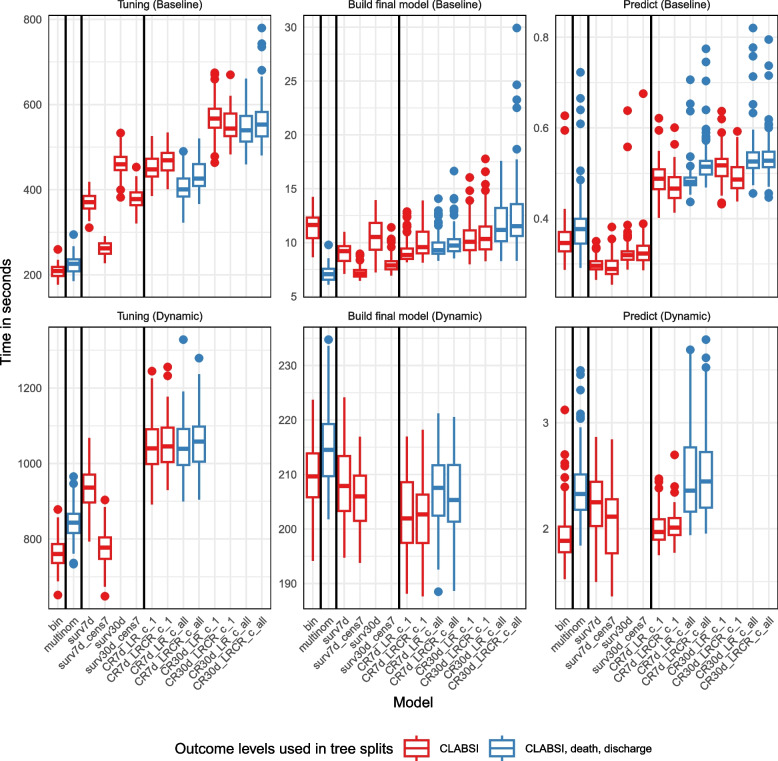
Runtimes for static (baseline) and dynamic models

The runtimes are presented in Fig. 6 and Supplementary material 9. The tuning time increases with model complexity, with binary, multinomial and the survival model with administrative censoring at day 7 and event censoring at day 7 being the fastest for both static and dynamic models; censoring at a later time (30 days) produces higher runtimes for static models and competing risks models take generally longest to tune. The runtimes vary less for the final model building, probably depending more on the final hyperparameter values. The prediction times also increase with model complexity but are fast for all models, with a median of less than one second for an entire baseline test set (on average slightly more than 10,000 observations) and less than two seconds for a dynamic test set (on average slightly more than 80,000 observations).

## Discussion

Survival and competing risks models are not widely used in machine learning prediction studies, and even less so on EHR data [23]. To our knowledge no other study compared RF models under competing risks settings. We compared different modelling options (binary, multinomial, survival, and competing risks outcome) for building static and dynamic random forest models for predicting the 7 days CLABSI risk using the randomforestSRC R package.

In our settings, complex models did not display a competitive advantage in terms of model performance. Moreover, complex models were slower to tune. Binary classification models remain the simplest choice for model building, implemented in a wide range of software libraries. Survival RF models that censor the competing event at its time of occurrence yield inferior calibration and discrimination compared to all the other modelling choices. Keeping the observations with competing events in the risk set until the prediction horizon, as done in Fine-Gray models [24], provides reliable estimates of the cumulative incidence function and superior prediction performance. This finding is potentially valuable for researchers with interest in predictions on multiple time horizons but unable to build a competing risks model due to software limitations. For instance, the ranger R package [25] supports survival models but not competing risks models; this might be the situation for other model choices or software libraries.

A companion study using regression models on the same dataset also found that binary, multinomial and competing risk models had similar performance [26]. The RF models in this analysis had higher discrimination: median AUROC up to 0.742 for static RF models and 0.775 for dynamic RF models at landmark 5, compared to 0.721 and 0.747 respectively for regression models. The calibration was similar for RF and regression models: median E:O ratio at LM5 between 0.95 and 0.97 for RF models and between 0.940 and 0.977 for regression models. Lower performance for survival models that censor the competing risks at their time of occurrence was also observed in the companion study, and this finding is in line with the extensive research done on statistical survival models [7].

For static models, different modeling approaches lead to similar model performance in terms of AUROC but different risk predictions in individuals. This is in line with a growing body of research studying the “between model” variability, which refers to differences in individual predictions arising from variations in model types [27] or feature selection choices [28]. We observed that models using all levels of the outcome (multinomial and competing risks) predicted higher risks than the other models, displayed higher AUPRC and slight miscalibration (higher ECI), but did not display considerable differences in AUROC. Despite the miscalibration, the BSS does not penalize these models, as there is gain of predictive performance in the high predicted risks. Zhou et al demonstrate that for low event rate outcomes, AUPRC puts larger weight on the model’s discriminating ability in high risk predictions compared AUROC and conduct a numerical study in which they observe that AUPRC is often correlated with the Brier Score [29]. Li et al. [30] conducted a study to predict cardiovascular disease in the presence of censoring using survival models (accounting for censoring) and ML methods that did not account for censoring. They observed that misspecified models (models that ignored censoring) produced lower, more conservative predicted risks and correctly specified models (not ignoring censoring) produced higher predicted risks while the differences in AUROC were minimal.

Despite extensive functionality in randomforestSRC (unsupervised and supervised learning with various outcome types, multiple split rules, missing data imputation, variable importance metrics, variable selection strategies) which makes it stand out in the software packages space, limitations exist with regards to tuning strategies, out-of-bag sampling and its feasibility for large datasets. The tuning objectives (e.g., logloss, Brier Score) cannot be changed in the default *tune* function of the package and the subsample size is not a tunable parameter; we have in change opted to tune the hyperparameters using mlrMBO [16] which offers great flexibility. The randomforestSRC package does not offer the possibility of user-defined in-bags and out-of-bags of different sample sizes, which are useful for dynamic and clustered data. Users can opt in turn for cross-validation, a slower procedure compared to tuning based on OOB predictions; we have opted to adjust the in-bags to a minimum size and gain computational efficiency. While this approach is in essence a test leak, its impact was negligible in our specific setting, as confirmed by comparisons with models built using the ranger package, which does not pose the constraint of equal size in-bags. Possibly admissions present both in-bag and out-of-bag could not be identified based on the small number of features included in our models. However, this approach should be carefully evaluated before being used in other contexts, such as a larger feature set, where it may introduce more pronounced bias. Even after applying all documented suggestions for improving computation time for survival and competing risks models [31], running these models on our large dynamic dataset would not have been feasible. Discretizing event times and applying administrative censoring, as explained in the Data and methods section, resulted in split statistics to be calculated over a limited number of horizons of interest and came with significant computational gain. This solution has broader applications; other studies resort to using only binary classification ML models for computational efficiency, even in the presence of censoring [30], which can lead to decreased sample sizes or introduce bias.

The current study has limitations. As a methodological comparison study, we neither recommend a specific model for clinical use nor evaluate the clinical usefulness of the models. We do not account for the correlation between repeated catheter episodes and landmarks within an admission, assuming that after adjusting for landmark and other features, any residual correlation is minimal. Our comparison between the static model and the dynamic model evaluated at LM0 showed that dynamic models generally outperformed static models in overall performance and discrimination, and had similar calibration, suggesting that ignoring this correlation did not negatively impact the predictive performance.

Two important topics remain to be studied further. First, we assume the only outcome of interest is CLABSI event in any of the next 7 days; the undeniable advantage of survival and competing risks models is that they can present the user with predictions for other time horizons in situations where one prediction horizon is of main interest but other horizons might be of secondary interest. Binary models are the fastest to tune, but if we were to build binary models for horizons 1 to 7 (either independent models or built with a monotonicity constraint on predictions), their computational advantage would most probably not persist. Moreover, we are not exploring multiple prediction horizons of interest (7, 14, 30,...) due to the high computational time on our large dataset.

Second, the models that use all levels of the outcome (multinomial and competing risks) display some particularities in tuned hyperparameters, variables used in early tree splits, distribution of predicted risks and slight advantages in some of the performance metrics. The difference in minimal depth for these models is most probably a consequence of optimizing the split statistic over multiple outcome classes. We selected the feature set with CLABSI prediction in mind and we did not explicitly include predictive features for death and discharge. Extensive research would be needed, either using simulated data or real datasets including strong predictors for all outcome levels to understand to which extent the feature selection impacts the model performance when multiple outcome levels are used.

## Conclusion

In our studied settings, complex models did not considerably improve the predictive performance compared to a binary model, which can be considered the easiest choice both in terms of model development and in computational time. Importantly, censoring the competing events at their time of occurrence should be avoided in survival models. More research is needed to study the impact of feature selection in models with multiple levels of outcome (multinomial and competing risks).

## Supplementary information

Additional file 1: Supplementary materials 1–11.

## Data Availability

The data used in this study cannot be shared publicly due to for the privacy of individuals that participated in the study.

## Abbreviations

AUPRC: Area under the precision recall curve
AUROC: Area under the receiver operating characteristic curve
BS: Brier Score
BSI: Bloodstream infection
BSS: Brier Skill Score
CICC: Centrally inserted central catheters
CIF: Cumulative incidence functions
CLABSI: Central line-associated bloodstream infections
CR: Competing risks
CRP: C-reactive protein
CVC: Central venous catheter
ECI: Estimated Calibration Index
EHR: Electronic Health Records
FWO: Research Foundation-Flanders
ICD: International Classification of Diseases
ICU: Intensive care unit
IQR: Interquartile range
KWS: Klinisch Werkstation (EHR System)
LC-BSI: Laboratory-confirmed bloodstream infection
LM: Landmark
LR: logrank
LRCR: logrankCR (logrank competing risks)
LWS: Laboratorium Werkstation (Laboratory System)
ML: Machine learning
NMSE: Normalized mean square error
OOB: Out of bag
PICC: Peripherally inserted central catheter
PDMS: Patient Data Management System (ICU Management System)
RAM: Random access memory
RF: Random Forest
TIVAD: Totally implanted vascular access devices
TPN: Total parenteral nutrition
VSC: Flemish Supercomputer Center
WBC: White blood cells count
