# Supplementary material for: A comparison of modeling approaches for static and dynamic prediction of central line-associated bloodstream infections using electronic health records (part 2): random forest models
Source: Diagn Progn Res. 2025 Jul 21;9:21. doi: 10.1186/s41512-025-00194-8 (PMC12278561; doi:10.1186/s41512-025-00194-8)
Supplement: Supplementary file 1 — Additional file 1: Supplementary materials 1–11. [file 41512_2025_194_MOESM1_ESM.pdf]

Supplementary material for "A Comparison of modeling approaches for static and dynamic prediction of central line-associated bloodstream infections using electronic health records (part 2): random forests models"

Supplementary material 1 - Example of stacked dataset for dynamic model building

An example of stacked dataset for dynamic model building is presented in S-Table 1. The admission id is kept in the dataset for train/test split but not retained for model building.

S-Table 1: Example of stacked data structure including some selected features; missing values are represented with NA; admission IDs are fictive.

Admission ID	Catheter episode	LM	CVC	Subclavian	TPN	Temperature	WBC	Event type	Event time
1	1	0	1	1	0	38.6	4.0	CLABSI	9.7
1	1	1	1	1	0	38.3	3.8	CLABSI	9.7
1	1	2	1	1	0	38.0	5.7	CLABSI	9.7
1	1	3	1	1	0	37.2	3.7	CLABSI	9.7
1	1	4	1	1	0	37.8	3.2	CLABSI	9.7
1	1	5	1	1	0	38.8	2.7	CLABSI	9.7
1	1	6	1	1	0	39.5	2.4	CLABSI	9.7
1	1	7	1	1	0	38.3	2.6	CLABSI	9.7
1	1	8	1	1	0	38.1	4.4	CLABSI	9.7
1	1	9	1	1	0	37.0	2.7	CLABSI	9.7
1	2	0	1	1	0	39.5	0.1	Death	1.2
1	2	1	1	1	0	39.8	0.2	Death	1.2
2	1	0	1	1	0	38.3	8.7	Discharge	3.5
2	1	1	1	1	0	38.1	NA	Discharge	3.5
2	1	2	1	1	0	37.1	9.1	Discharge	3.5
2	1	3	1	1	0	37.0	NA	Discharge	3.5
3	1	0	1	1	0	37.2	5.6	Discharge	4.2
3	1	1	1	1	0	36.6	7.2	Discharge	4.2
3	1	2	1	1	0	36.5	8.3	Discharge	4.2
3	1	3	1	1	0	37.1	NA	Discharge	4.2
3	1	4	1	1	0	37.0	6.2	Discharge	4.2
3	2	0	1	1	0	37.7	NA	Discharge	2.5
3	2	1	1	1	0	37.3	6.2	Discharge	2.5
3	2	2	1	1	0	37.4	NA	Discharge	2.5

Supplementary material 2 - Features descriptions

Baseline variables are invariant for a catheter episode and are known at the start of the catheter episode. Time-varying features represent features that can vary from one landmark to another.

Continuous features: Whenever multiple measurements are available during a time window (typically 24 hours) for continuous features these are aggregated into a single landmark value. The aggregation rule (e.g.: maximum, minimum) with the most clinical significance is chosen (e.g.: maximum temperature in last 24 hours). Whenever no measurements are taken in the time window, the feature value is represented as a missing value.

Binary features are coded for presence or absence of specific clinical events that are recorded in the EHR only when present, e.g.: total parenteral nutrition (TPN).

Categorical features (e.g.: catheter type) that might occur simultaneously in the aggregation window are coded as binary features (0/1) for all values recorded (e.g.: if a patient has two catheters: CICC or TIVAD, two binary features are kept for CICC or TIVAD). Whenever categorical features (with two or more categories) are expected to be recorded regardless of the category (e.g.: admission source), and no value is present, the feature value is represented as a missing value.

Values outside the possible range have been deleted before feature aggregation, for the following variables and ranges (min – max): temperature, [30, 45]; systolic and diastolic blood pressure, [30, 370]; respiratory rate, [0, 900]; heart rate, [0, 500]; oxygen saturation, [0, 100]; CVP (central venous pressure), [-5, 20]; weight, [0.05, 250]; length, [0.05, 250]; glycemia, [0, 2000]. These deletions might result in missing values to be imputed later.

The descriptions of variables selected for the model building are presented in S-Table 2.

S-Table 2: Features included in the model (binary_all means that all feature values encountered in the aggregation window are kept as binary values (0/1) for categorical features

Short name	Feature	Description	Type	Baseline / time-varying
CVC	CAT_catheter_type_binary_all_CVC	Was there a catheter of type CICC connected since previous LM?	binary	TV
Port catheter	CAT_catheter_type_binary_all_Port_a_cath	Was there a catheter of type TIVAD connected since previous LM?	binary	TV
Tunneled CVC	CAT_catheter_type_binary_all_Tunneled_CVC	Was there a catheter of type t-CICC or tc-CICCconnected since previous LM?	binary	TV
PICC	CAT_catheter_type_binary_all_PICC	Was there a catheter of type PICC connected since previous LM?	binary	TV
Jugular	CAT_catheter_location_binary_all_Collarbone	Was there a catheter connected at location Collarbone since previous LM?	binary	TV
Subclavian	CAT_catheter_location_binary_all_Neck	Was there a catheter connected at location Neck since previous LM?	binary	TV
CLABSI history	CLABSI_history	Did the patient experience a CLABSI event in the past 3 months since LM time?	binary	TV
Admission source Home	ADM_admission_source_binary_all_Home	Admission source (home or other places)	binary	BASE
TPN	MED_7d_TPN	Has TPN (total parenteral nutrition) been ordered for the patient in the previous 7 days from LM time	binary	TV
AB	MED_L2_7d_J01_ANTIBACTERIALS_FOR_SYSTEMIC_USE	Have any drugs in ATC group (level 2) J01 (ANTIBACTERIALS FOR SYSTEMIC USE) been ordered for the patient in the previous 7 days from LM time	binary	TV
Chemotherapy	MED_L2_7d_L01_ANTINEOPLASTIC_AGENTS	Have any drugs in ATC group (level 2) L01 (ANTINEOPLASTIC AGENTS) been ordered for the patient in the previous 7 days from LM time	binary	TV
Systolic BP	CARE_VS_systolic_BP_last	Last value of systolic blood pressure since previous landmark. For baseline (LM 0) the last value from the previous 24 hours is used.	cont	TV
Temperature	CARE_VS_temperature_max	Maximum value of temperature since previous landmark. For baseline (LM 0) the last value from the previous 24 hours is used. Only temperatures in the range (30 °C, 45 °C) are kept, the others are deleted. Maximum value is used to correct for very low temperatures measured by devices in ICU, when the temperature falls closer to the room temperature.	cont	TV
MV	CARE_VS_MV	Is the patient on mechanical ventilation (MV) since previous landmark? A patient is considered on MV if at least one value of PEEP or FiO2 are recorded between 2 landmarks. Only valid for ICU patients	binary	TV
ICU	MS_is_ICU_unit	Is the patient now (at the exact second of the current LM) in ICU?	binary	TV
Lymphoma history	COM_lymphoma_before_LM	Has lymphoma been registered as a comorbidity before current LM time?	binary	TV
Tumor history	COM_PATH_tumor_before_LM	Has a tumour pathology been registered before current LM time?	binary	TV
Transplant history	COM_PATH_transplant_before_LM	Has a transplant pathology been registered before current LM time?	binary	TV
CRP	LAB_CRP_last	CRP, last value since previous LM. Unit: mg/L	cont	TV
WBC	LAB_WBC_count_last	WBC count, last value since previous LM. Unit: 10**9/L	cont	TV
Other infection than BSI	MB_other_infection_than_BSI_during_window	Has there been a positive culture, of any other type than blood, in the last 17 days (time window used for secondary BSIs). The validation time of the sample is used (as opposed to the CLABSI calculation, where the date foreseen for the sample collection is used)	binary	TV

Supplementary material 3 - Cumulative incidence function curves

Cumulative incidence function curves for all events are presented in S-Figure 1. Cumulative incidence function curves for death and discharge are presented in S-Figure 2.

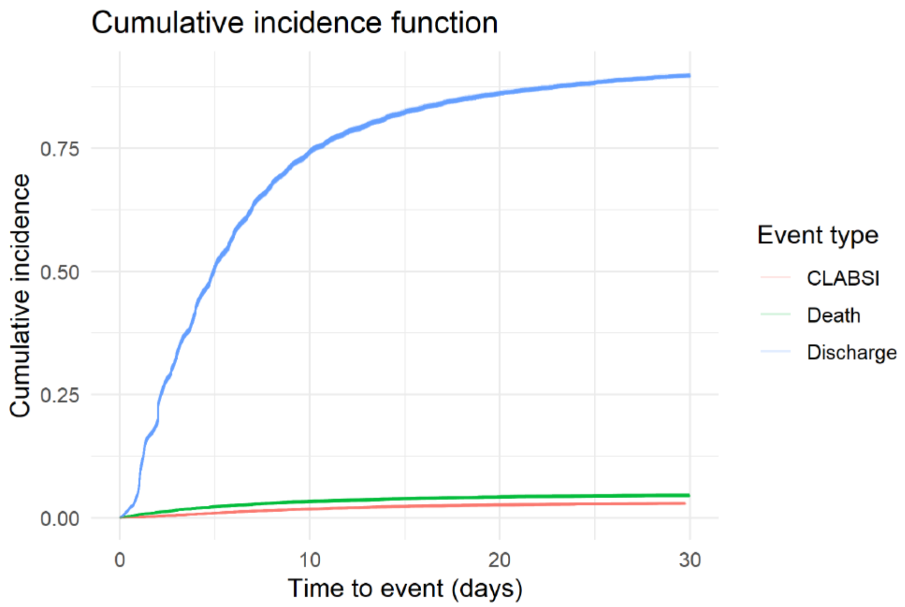

S-Figure 1: Cumulative incidence function curves for all events (all train sets)

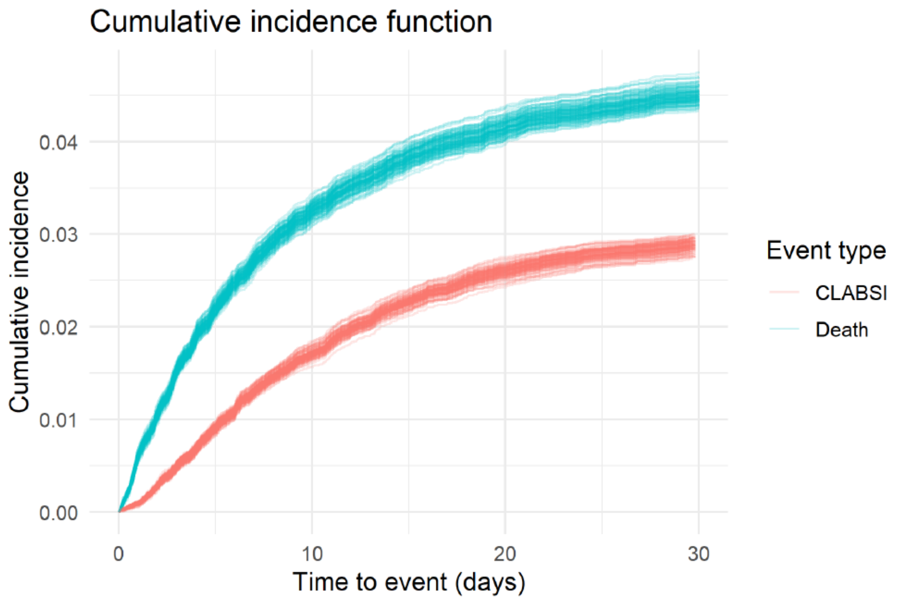

S-Figure 2: Cumulative incidence function curves for CLABSI and Death events (all train sets)

Supplementary material 4 - Missing data imputation

Missing data have been imputed in the larger context (all 302 features) using a combination of mean/mode imputation, normal value imputation and the missForestPredict algorithm (Albu 2023). The test sets are imputed using the mean/mode or the missForestPredict models learned on the train sets. S-Table 3 describes the missingness of the features included in the baselin1e and dynamic prediction models.

Missing data imputation has been performed separately for baseline data (using only the data available at LM0) and for the dynamic data ("pooling" together all landmarks from all catheter episodes as independent observations, as in the dataset example in S-Table 1. We have separated the baseline imputation, as we consider that studies presenting static prediction models utilize only data available at baseline. Three steps have been applied in the imputation process:

- Feature exclusion based on missingness rate or sparsity
- Simple imputation
- missForestPredict imputation

S-Table 3: Features included in the model with missing values; for baseline datasets, catheter episodes are the unit of observation; for dynamic datasets landmarks are the unit of observation

Feature	Number present	Number missing	Percentage missing	Imputation strategy	Rationale	Baseline/dynamic
Admission source Home	30365	497	1.6%	mode	less than 3% missing	Baseline
Temperature	22919	7943	25.7%	missForestPredict		Baseline
Systolic BP	22616	8246	26.7%	missForestPredict		Baseline
WBC	17990	12872	41.7%	missForestPredict		Baseline
CRP	16480	14382	46.6%	missForestPredict		Baseline
Admission source Home	226244	1684	0.74%	mode	less than 3% missing	Dynamic
Temperature	215107	12821	5.63%	missForestPredict		Dynamic
Systolic BP	205661	22267	9.77%	missForestPredict		Dynamic
WBC	137581	90347	39.64%	missForestPredict		Dynamic
CRP	135015	92913	40.76%	missForestPredict		Dynamic

4.1 Feature exclusion based on missingness rate or sparsity

Before imputation, some preliminary cleaning has been performed using the following rules for both baseline and dynamic data:

- Based on an exploration of missingness over time, features completely missing for a part of the study timeframe have been excluded (e.g.: RASS, Richmond Agitation Sedation Scale started being recorded in the system as from February 2013 and was completely missing before).
- Features with less than 500 non-missing values are excluded; we consider that building an imputation model for a feature that has less than 500 non-missing values might prove unstable. Applying this rule will result in a different set of excluded features at baseline than in the dynamic data. As dynamic data includes all landmarks, some features that have less than 500 recorded values at baseline will have more than 500 values across all landmarks.
- Some sparse levels in the categorical feature "medical specialty" (less than 200 observations in a category) have been collapsed into category "Other".

4.2 Simple imputation

Further, for the three situations below, features have been imputed at baseline using mean/mode, normal value or based on clinical knowledge and using last-observation-carried-forward (LOCF) in the dynamic data. In case of mean/mode imputation, the mean/mode of the training set is used to impute the test set.

- Missing rate less than 3%
- Features that we consider "difficult to impute". To assess if a feature is "difficult to impute" we have run the missForestPredict algorithm using one iteration on the full baseline and dynamic datasets before train/test split and inspected the OOB (out-of-bag) normalized mean square error (NMSE) (Albu 2023). We have used only one iteration because at the first iteration the OOB error is less subject to bias than at later iterations (when imputed values of one feature are reinforced by imputed values of another feature that in turn was imputed based on the first feature). A value of NMSE close to 1 means that the algorithm does not provide imputations much superior to the mean imputation. Features with OOB NMSE at first iteration greater than 0.9 have been considered "difficult to impute."
- The number of catheter lumens is a special situation because their imputation makes sense only within the categories of catheter types. They have been imputed with typical (normal) values or based on clinical knowledge.

The baseline and dynamic features included and the imputation strategy are listed in the tables below.

After imputation of lumens per catheter type, the total number of lumens has been calculated and kept as a feature and the number of lumens per catheter type features has been removed.

4.3 missForestPredict imputation

For the remaining features with missing values, missing data imputation has been performed on each training set using the missForestPredict algorithm (Albu 2023). Complete features have been included in imputation as "predictors" for the features with missing values. The missing values are first imputed with mean/mode and then iteratively imputed using random forest models for 5 iterations. The default hyperparameter values of the ranger function are used for random forest imputation models except the maximum tree depth which has been set to 10. The outcome is not included in the imputation. Patient length and weight missing data indicators have been preserved before imputation; after imputation, the BMI has been calculated and the patient length and weight have been deleted.

Test sets have been imputed using the missForestPredict imputation models learned on training data.

All features included in the imputation for the baseline dataset are presented in S-Table 4. All features included in the imputation for the dynamic dataset are presented in S-Table 5. The OOB errors (NMSE) for each iteration are presented in S-Figures 3 and 4 Each line represents the NMSE error over iterations for one of the 100 training sets.

S-Table 4: Features and imputation method (baseline)

Feature	N present	N missing	% missing	Imputation strategy	Rationale
CAT_lumens_PICC	30800	62	0,2%	1 (fixed value)	Lumens special situation
CAT_lumens_Tunneled_CVC	30775	87	0,3%	3 (fixed value)	Lumens special situation
MS_medical_specialty	30701	161	0,5%	mode	<3% missing
MS_alternative_flag	30701	161	0,5%	mode	<3% missing
ADM_admission_referral_binary_all_GP	30379	483	1,6%	mode	<3% missing
ADM_admission_source_binary_all_Home	30365	497	1,6%	mode	<3% missing
ADM_admission_reason_binary_all_Accident	30301	561	1,8%	mode	<3% missing
ADM_admission_type_binary_all_Emergency	30245	617	2,0%	mode	<3% missing
CAT_lumens_CVC	25841	5021	16,3%	2 (fixed value)	Lumens special situation
CARE_VS_temperature_max	22919	7943	25,7%	missForestPredict	
CARE_VS_heart_rate_max	22851	8011	26,0%	missForestPredict	
CARE_VS_systolic_BP_last	22616	8246	26,7%	missForestPredict	
CARE_SAF_mobility_assistance_binary_all_partial_help	21782	9080	29,4%	missForestPredict	
CARE_SAF_mobility_assistance_binary_all_no_help	21782	9080	29,4%	missForestPredict	
CARE_SAF_mobility_assistance_binary_all_full_help	21782	9080	29,4%	missForestPredict	
LAB_is_neutropenia	18032	12830	41,6%	missForestPredict	
LAB_Hemoglobine_last	18010	12852	41,6%	missForestPredict	
LAB_WBC_count_last	17990	12872	41,7%	missForestPredict	
LAB_Platelet_count_last	17948	12914	41,8%	missForestPredict	
LAB_RBC_count_last	17865	12997	42,1%	missForestPredict	
LAB_haematocrit_last	17851	13011	42,2%	missForestPredict	
LAB_potassium_last	17409	13453	43,6%	missForestPredict	
LAB_natrium_last	17401	13461	43,6%	missForestPredict	
LAB_creatinine_last	17319	13543	43,9%	missForestPredict	
LAB_urea_last	17291	13571	44,0%	missForestPredict	
LAB_CRP_last	16480	14382	46,6%	missForestPredict	
CAT_bandage_type_binary_last_polyurethane	16469	14393	46,6%	missForestPredict	
CAT_bandage_type_binary_last_gauze	16469	14393	46,6%	missForestPredict	
CAT_needle_length_max	13747	17115	55,5%	mode	Difficult to impute
LAB_PT_sec_last	13668	17194	55,7%	missForestPredict	
LAB_PT_INR_last	13664	17198	55,7%	missForestPredict	
LAB_PT_percent_last	13663	17199	55,7%	missForestPredict	
LAB_APTT_last	11038	19824	64,2%	missForestPredict	
LAB_O2_saturation_last	10709	20153	65,3%	missForestPredict	
LAB_pO2_last	10703	20159	65,3%	missForestPredict	
LAB_pH_last	10695	20167	65,3%	missForestPredict	
LAB_glucose_arterial_last	10621	20241	65,6%	missForestPredict	
CARE_PHY_weight_mean	10498	20364	66,0%	missForestPredict	
LAB_AST_last	10412	20450	66,3%	missForestPredict	
LAB_ALT_last	10372	20490	66,4%	missForestPredict	
LAB_WBC_Neutrophils_last	10282	20580	66,7%	missForestPredict	
LAB_WBC_Monocytes_last	10254	20608	66,8%	missForestPredict	
LAB_bilirubin_last	10000	20862	67,6%	missForestPredict	
LAB_glucose_last	9972	20890	67,7%	missForestPredict	
CAT_bandage_observation_binary_all_Abnormal	9185	21677	70,2%	missForestPredict	
CARE_VS_oxygen_saturation_last	8954	21908	71,0%	missForestPredict	
LAB_LDH_last	8729	22133	71,7%	missForestPredict	
CARE_WND_wound_type_binary_all_open_wound	5557	25305	82,0%	missForestPredict	
CARE_WND_wound_type_binary_all_closed_wound	5557	25305	82,0%	missForestPredict	
CARE_WND_wound_type_binary_all_suture_and_post	5557	25305	82,0%	missForestPredict	
CARE_PHY_length_mean	3923	26939	87,3%	missForestPredict	
CARE_SAF_patient_position_binary_all_Fowler	3809	27053	87,7%	missForestPredict	
CARE_SAF_patient_position_binary_all_lateral	3809	27053	87,7%	missForestPredict	
CARE_SAF_patient_position_binary_all_supine	3809	27053	87,7%	missForestPredict	
CARE_SAF_patient_position_binary_all_sitting	3809	27053	87,7%	missForestPredict	
LAB_CK_last	3661	27201	88,1%	112 for M, 88 for F ('normal' value)	Difficult to impute
CARE_VS_respiratory_rate_last	3447	27415	88,8%	missForestPredict	
LAB_fibrinogen_last	2057	28805	93,3%	missForestPredict	
CARE_NEU_GCS_score_last	2028	28834	93,4%	missForestPredict	
LAB_TSH_last	1159	29703	96,2%	2.5 ('normal' value)	Difficult to impute
LAB_ferritin_last	1069	29793	96,5%	missForestPredict	
LAB_D_dimer_last	791	30071	97,4%	missForestPredict	
CARE_VS_CVP_last	710	30152	97,7%	8 ('normal' value)	Difficult to impute

S-Table 5: Features and imputation method (dynamic)

Feature	N present	N missing	% missing	Imputation strategy	Rationale
MS_medical_specialty	227736	192	0,1%	mode	<3% missing
MS_alternative_flag	227736	192	0,1%	mode	<3% missing
CAT_lumens_PICC	226439	1489	0,7%	1 (fixed value)	Lumens special situation
CAT_lumens_Tunneled_CVC	226419	1509	0,7%	3 (fixed value)	
CAT_lumens_CVC	181021	46907	20,6%	2 (fixed value)	
ADM_admission_referral_binary_all_GP	226371	1557	0,7%	mode	<3% missing
ADM_admission_source_binary_all_Home	226244	1684	0,7%	mode	<3% missing
ADM_admission_reason_binary_all_Accident	225772	2156	0,9%	mode	<3% missing
ADM_admission_type_binary_all_Emergency	225046	2882	1,3%	mode	<3% missing
CARE_VS_temperature_max	215107	12821	5,6%	missForestPredict	
CARE_VS_heart_rate_max	207451	20477	9,0%	missForestPredict	
CARE_VS_systolic_BP_last	205661	22267	9,8%	missForestPredict	
CARE_SAF_mobility_assistance_binary_all_partial_help	173326	54602	24,0%	missForestPredict	
CARE_SAF_mobility_assistance_binary_all_no_help	173326	54602	24,0%	missForestPredict	
CARE_SAF_mobility_assistance_binary_all_full_help	173326	54602	24,0%	missForestPredict	
CAT_bandage_type_binary_last_polyurethane	170157	57771	25,3%	missForestPredict	
CAT_bandage_type_binary_last_gauze	170157	57771	25,3%	missForestPredict	
CAT_bandage_observation_binary_all_Normal	166577	61351	26,9%	missForestPredict	
CAT_bandage_observation_binary_all_Bloody_or_Moist	166577	61351	26,9%	missForestPredict	
CAT_bandage_observation_binary_all_Red	166577	61351	26,9%	missForestPredict	
CAT_bandage_observation_binary_all_Other_Hema_Pus_Loose_Necro	166577	61351	26,9%	missForestPredict	
LAB_is_neutropenia	138216	89712	39,4%	missForestPredict	
LAB_Hemoglobine_last	137959	89969	39,5%	missForestPredict	
LAB_WBC_count_last	137581	90347	39,6%	missForestPredict	
LAB_potassium_last	137361	90567	39,7%	missForestPredict	
LAB_natrium_last	137200	90728	39,8%	missForestPredict	
LAB_Platelet_count_last	136846	91082	40,0%	missForestPredict	
LAB_creatinine_last	136062	91866	40,3%	missForestPredict	
LAB_urea_last	135748	92180	40,4%	missForestPredict	
LAB_RBC_count_last	135566	92362	40,5%	missForestPredict	
LAB_haematocrit_last	135330	92598	40,6%	missForestPredict	
LAB_CRP_last	135015	92913	40,8%	missForestPredict	
CARE_WND_wound_type_binary_all_suture	107641	120287	52,8%	missForestPredict	
CARE_WND_wound_type_binary_all_open_wound	107641	120287	52,8%	missForestPredict	
CARE_WND_wound_type_binary_all_post_suture	107641	120287	52,8%	missForestPredict	
CARE_WND_wound_type_binary_all_closed_wound	107641	120287	52,8%	missForestPredict	
CARE_VS_oxygen_saturation_last	103740	124188	54,5%	missForestPredict	
LAB_bilirubin_last	97977	129951	57,0%	missForestPredict	
LAB_AST_last	81969	145959	64,0%	missForestPredict	
LAB_ALT_last	81759	146169	64,1%	missForestPredict	
LAB_PT_sec_last	77964	149964	65,8%	missForestPredict	
LAB_PT_INR_last	77948	149980	65,8%	missForestPredict	
LAB_PT_percent_last	77946	149982	65,8%	missForestPredict	
LAB_LDH_last	73269	154659	67,9%	missForestPredict	
CAT_needle_length_max	68161	159767	70,1%	missForestPredict	
CARE_SAF_patient_position_binary_all_Fowler	67789	160139	70,3%	missForestPredict	
CARE_SAF_patient_position_binary_all_lateral	67789	160139	70,3%	missForestPredict	
CARE_SAF_patient_position_binary_all_supine	67789	160139	70,3%	missForestPredict	
CARE_SAF_patient_position_binary_all_sitting	67789	160139	70,3%	missForestPredict	
LAB_WBC_Neutrophils_last	64666	163262	71,6%	missForestPredict	
LAB_WBC_Monocytes_last	64165	163763	71,8%	missForestPredict	
LAB_APTT_last	63028	164900	72,3%	missForestPredict	
CARE_PHY_weight_mean	56798	171130	75,1%	missForestPredict	
CARE_VS_respiratory_rate_last	46708	181220	79,5%	missForestPredict	
LAB_pO2_last	46426	181502	79,6%	missForestPredict	
LAB_O2_saturation_last	46396	181532	79,6%	missForestPredict	
LAB_pH_last	46385	181543	79,6%	missForestPredict	
LAB_glucose_last	45920	182008	79,9%	missForestPredict	
LAB_glucose_arterial_last	45875	182053	79,9%	missForestPredict	
CAT_lumens_flushed	41384	186544	81,8%	missForestPredict	
CARE_NEU_GCS_score_last	39412	188516	82,7%	missForestPredict	
CARE_VS_CVP_last	31985	195943	86,0%	missForestPredict	
LAB_CK_last	22477	205451	90,1%	missForestPredict	
LAB_vancomycine_last	8912	219016	96,1%	missForestPredict	

CAT_result_infusion_binary_all_normal	7565	220363	96,7%	missForestPredict	
CAT_result_infusion_binary_all_difficult	7565	220363	96,7%	missForestPredict	
CAT_result_infusion_binary_all_impossible	7565	220363	96,7%	missForestPredict	
CAT_result_aspiration_binary_all_normal	7564	220364	96,7%	missForestPredict	
CAT_result_aspiration_binary_all_difficult	7564	220364	96,7%	missForestPredict	
CAT_result_aspiration_binary_all_impossible	7564	220364	96,7%	missForestPredict	
CARE_PHY_length_mean	6846	221082	97,0%	missForestPredict	
LAB_fibrinogen_last	6837	221091	97,0%	missForestPredict	
LAB_aspergillus_ag_last	5450	222478	97,6%	missForestPredict	
LAB_ferritin_last	3969	223959	98,3%	missForestPredict	
LAB_TSH_last	3689	224239	98,4%	missForestPredict	
LAB_creatinine_clearance_last	2917	225011	98,7%	missForestPredict	
LAB_SPE_albumin_last	2665	225263	98,8%	missForestPredict	
LAB_SPE_albumin_alpha_1_globulin_last	2665	225263	98,8%	missForestPredict	
LAB_SPE_albumin_alpha_2_globulin_last	2665	225263	98,8%	missForestPredict	
LAB_SPE_albumin_beta_globulin_last	2665	225263	98,8%	missForestPredict	
LAB_SPE_albumin_gamma_globulin_last	2665	225263	98,8%	missForestPredict	
LAB_ciclosporin_last	2409	225519	98,9%	missForestPredict	
LAB_D_dimer_last	2009	225919	99,1%	missForestPredict	

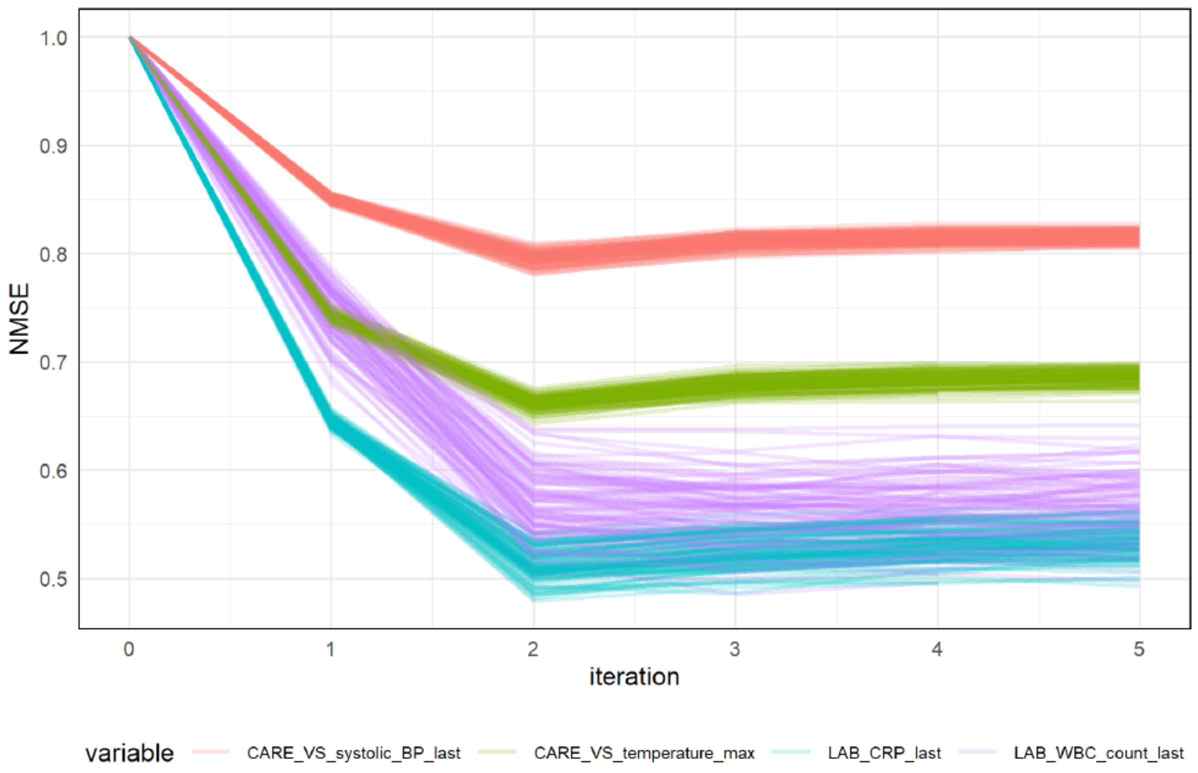

S-Figure 3: OOB NMSE for baseline imputation (features included in the model) for all train sets

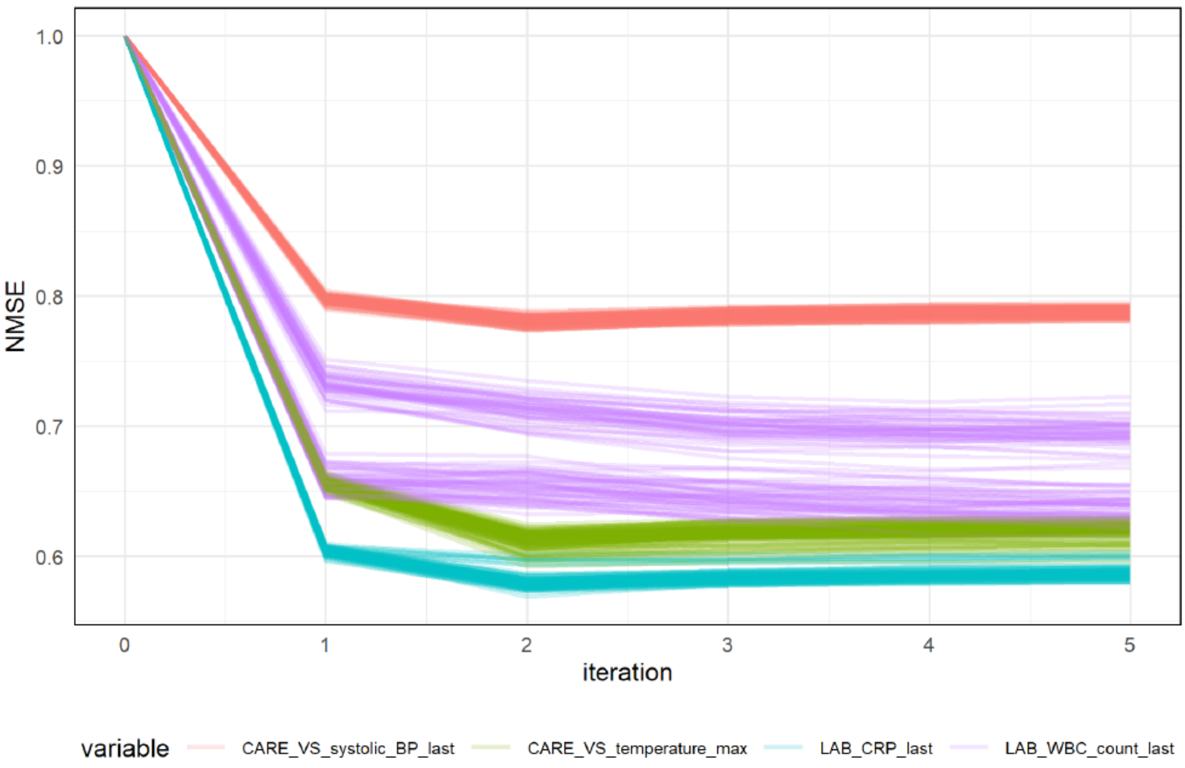

S-Figure 4: OOB NMSE for dynamic imputation (features included in the model) for all train sets

Supplementary material 5 - Comparison to ranger model

We have investigated the potential bias introduced by allowing a small number of observations belonging to the same admission to fall both in-bag and out-of-bag, incentivizing the tuning strategy to lean towards models that would memorize such admissions. This procedure affects on average (over all dynamic train sets and all inbags) between 1.1% and 1.7% of the inbag observations depending on the subsample size. We have performed a comparison for the static and dynamic binary and multinomial models against models built using the ranger package (Wright et al. 2019), which does not impose the limitation of equal size inbags, using the same inbags before adjusting for minimum size and we see no noticeable difference in results: S-Figure 5 for static models and S-Figure 6 and 7 for dynamic models. The models are evaluated on the test sets following the same procedure as in the main results. Given the small extent to which bias might potentially leak in and considering that all models (binary, multinomial, survival, CR) would suffer from the same bias, we will consider our comparisons valid under this limitation.

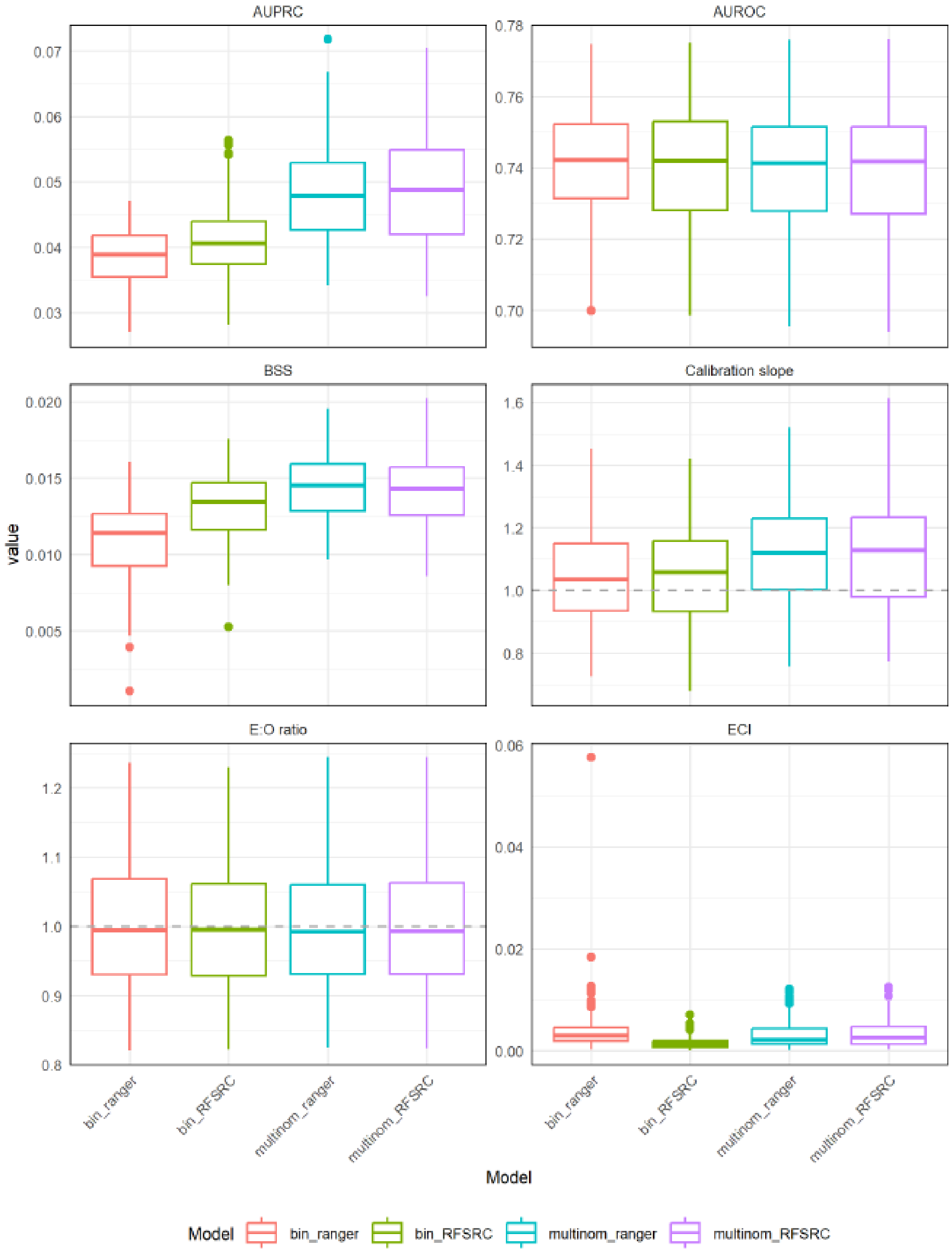

S-Figure 5: Prediction performance for static models (ranger vs. RFSRC)

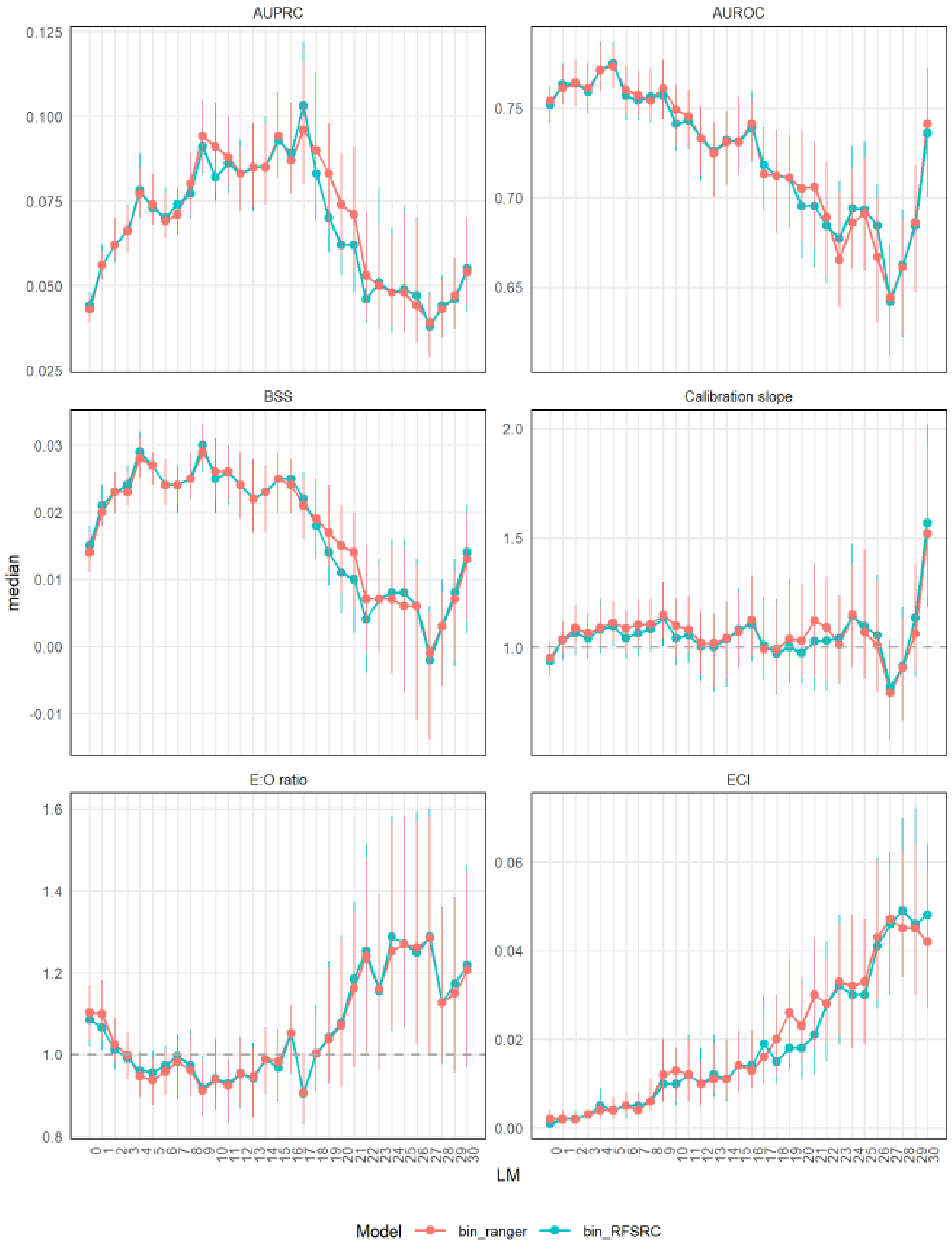

S-Figure 6: Prediction performance for binary dynamic models - time dependent metrics (ranger vs. RFSRC)

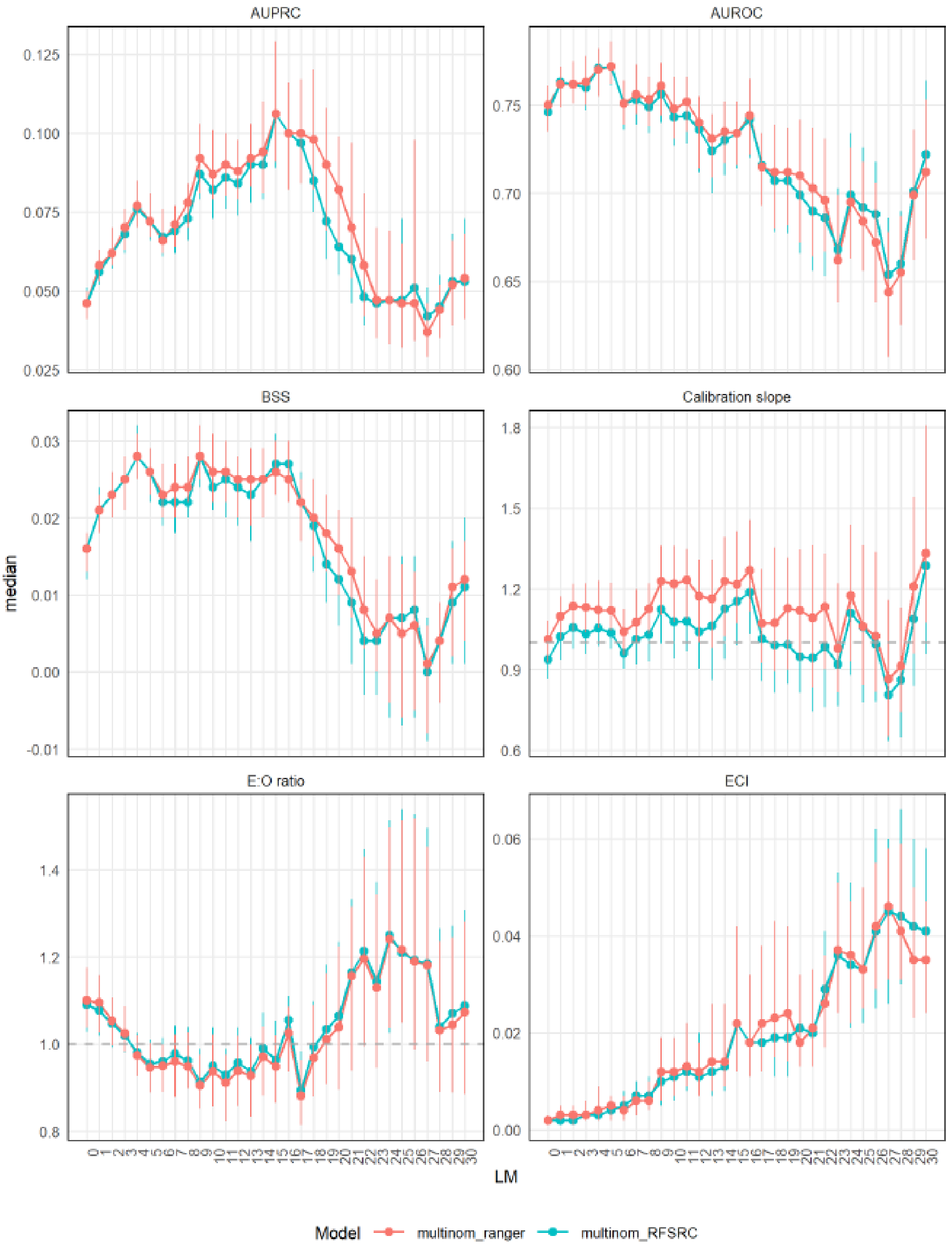

S-Figure 7: Prediction performance for multinomial dynamic models - time dependent metrics (ranger vs. RFSRC)

Supplementary material 6 - Additional documentation for methods

6.1 Model tuning

The tuned hyperparameters are the number of variables selected at each split (mtry) and the minimum size of a terminal node (nodesize) for both static and dynamic models. Additionally for dynamic models, the subsample size is tuned. The candidate tuned values of the hyperaparameters are limited to a min-max range: 2 to 15 for mtry, 50 to 4000 for minimum node size and 30% to 80% for the subsample size. The tuning is based on the out-of-bag binary logloss for next 7 days. Model based optimization tuning is performed using the mlrMBO R package (Bischl et al. 2017) with 20 design steps (randomly selected hyperparameter value combinations) and 30 optimization steps (aimed to find better hyperparameter values that minimize the logloss). An initial “design” is generated by randomly picking 20 points in the hyperparameter space (within the min-max range for each hyperparameter). Models are fit u sing the “ design” hyperparameter values and evaluated using the evaluation metric (logloss). We have used the out-of-bag logloss for the binary outcome (CLABSI within 7 days) as evaluation metric for all models, as we prefer to tune the models against the outcome of interest (instead of using survival metrics, for example). A “surrogate function” is fit through these design points modelling the evaluation metric (logloss) in function of the hyperparameters (mtry, node size, subsample size). A Kriging model is used for the “surrogate function” which performs well for continuous hyperparameters (Roustant, Ginsbourger, and Deville 2012). Further, 30 “optimization” steps are performed. At each step, new points in the hyperparameter space are proposed with either expected good model performance or with potential to improve the “surrogate function” (“trade-off exploitation and exploration”, Bischl et al. (2017)). Finally, the best hyperparameters from the 50 steps are chosen and a random forest model is built using these hyperparameters.

6.2 Linear analogues of RF models

A correspondence between the built RF models and linear models have been attempted in S-Table 6. The correspondence is established based on the outcome type and split rule of the RF and it is possibly not a perfect equivalence.

S-Table 6: Models

Model name	Outcome type	Splitrule	Other hyperparameters / options	Linear model analogue	Static model	Dynamic model
bin	Binary	gini		Logistic regression with binary outcome	YES	YES
multinom	Multinomial	gini		Multinomial logistic regression	YES	YES
surv7d	Survival	logrank	Administrative censoring at day 7; Censoring death and discharge at event time	Cox proportional hazards with censoring for competing events at their event time and administrative censoring at day 7	YES	YES
surv7d_cens7	Survival	logrank	Administrative censoring at day 7; Censoring death and discharge at time 7	Cox proportional hazards with censoring for competing events at day 7 and administrative censoring at day 7; also analogue to Fine-Gray subdistribution hazard model with administrative censoring at day 7 (in the presence of no other censoring)	YES	YES
surv30d	Survival	logrank	Administrative censoring at day 30; Censoring death and discharge at event time	Cox proportional hazards with censoring for competing events at their event time and administrative censoring at day 30	YES	NO
surv30d_cens7	Survival	logrank	Administrative censoring at day 30; Censoring death and discharge at time 7	Cox proportional hazards with censoring for competing events at day 7 and administrative censoring at day 30; also analogue to Fine-Gray subdistribution hazard model with administrative censoring at day 30 (in the presence of no other censoring)	YES	NO
CR7d_LRCR_c_1	Competing risks	logrankCR	cause = 1 (CLABSI); Administrative censoring at day 7	Fine-Gray subdistribution hazard model with administrative censoring at day 7	YES	YES
CR7d_LR_c_1	Competing risks	logrank	cause = 1 (CLABSI); Administrative censoring at day 7	No analogue	YES	YES
CR7d_LRCR_c_all	Competing risks	logrankCR	cause = default (all events have equal weights); Administrative censoring at day 7	No analogue	YES	YES
CR7d_LR_c_all	Competing risks	logrank	cause = default (all events have equal weights); Administrative censoring at day 7	Cause-specific hazard regression model with administrative censoring at day 7	YES	YES
CR30d_LRCR_c_1	Competing risks	logrankCR	cause = 1 (CLABSI); Administrative censoring at day 30	Fine-Gray subdistribution hazard model with administrative censoring at day 30	YES	NO
CR30d_LR_c_1	Competing risks	logrank	cause = 1 (CLABSI); Administrative censoring at day 30	No analogue	YES	NO
CR30d_LRCR_c_all	Competing risks	logrankCR	cause = default (all events have equal weights); Administrative censoring at day 30	No analogue	YES	NO
CR30d_LR_c_all	Competing risks	logrank	cause = default (all events have equal weights); Administrative censoring at day 30	Cause-specific hazard regression model with administrative censoring at day 30	YES	NO

Supplementary material 7 - Additional performance evaluation – static models

7.1 Performance metrics table

The performance of static models is presented in S-Table 7.

S-Table 7: Performance metrics table for static models

Model	BSS	AUPRC	AUROC	Calibration slope	ECI	E:O ratio
bin	0.013 (0.012 - 0.015)	0.041 (0.037 - 0.044)	0.742 (0.728 - 0.753)	1.058 (0.933 - 1.157)	0.001 (0.001 - 0.002)	0.995 (0.929 - 1.062)
multinom	0.014 (0.013 - 0.016)	0.049 (0.042 - 0.055)	0.742 (0.727 - 0.751)	1.128 (0.979 - 1.234)	0.003 (0.001 - 0.005)	0.993 (0.931 - 1.063)
surv7d	0.01 (0.007 - 0.012)	0.038 (0.036 - 0.043)	0.729 (0.716 - 0.745)	1.211 (1.115 - 1.35)	0.005 (0.004 - 0.007)	1.444 (1.353 - 1.541)
surv7d_cens7	0.013 (0.012 - 0.015)	0.041 (0.038 - 0.044)	0.742 (0.729 - 0.753)	1.04 (0.944 - 1.162)	0.001 (0.001 - 0.002)	0.996 (0.929 - 1.063)
surv30d	0.009 (0.007 - 0.012)	0.039 (0.037 - 0.043)	0.724 (0.713 - 0.74)	1.259 (1.14 - 1.387)	0.005 (0.004 - 0.007)	1.469 (1.375 - 1.565)
surv30d_cens7	0.014 (0.012 - 0.015)	0.042 (0.039 - 0.047)	0.739 (0.728 - 0.752)	1.092 (0.99 - 1.169)	0.001 (0.001 - 0.002)	0.995 (0.93 - 1.06)
CR7d_LR_c_1	0.013 (0.012 - 0.014)	0.041 (0.037 - 0.044)	0.739 (0.725 - 0.749)	1.116 (0.994 - 1.234)	0.001 (0.001 - 0.003)	0.996 (0.934 - 1.061)
CR7d_LRCR_c_1	0.013 (0.012 - 0.015)	0.041 (0.037 - 0.045)	0.742 (0.729 - 0.752)	1.062 (0.947 - 1.157)	0.001 (0.001 - 0.002)	0.995 (0.929 - 1.062)
CR7d_LR_c_all	0.014 (0.012 - 0.015)	0.047 (0.042 - 0.052)	0.736 (0.724 - 0.75)	1.133 (1.052 - 1.255)	0.003 (0.001 - 0.005)	0.991 (0.931 - 1.063)
CR7d_LRCR_c_all	0.014 (0.012 - 0.015)	0.047 (0.042 - 0.054)	0.738 (0.727 - 0.75)	1.151 (1.04 - 1.242)	0.002 (0.001 - 0.004)	0.99 (0.93 - 1.062)
CR30d_LR_c_1	0.013 (0.011 - 0.015)	0.042 (0.039 - 0.047)	0.737 (0.725 - 0.751)	1.111 (1.035 - 1.213)	0.001 (0.001 - 0.003)	0.995 (0.931 - 1.06)
CR30d_LRCR_c_1	0.014 (0.012 - 0.015)	0.043 (0.04 - 0.047)	0.741 (0.729 - 0.752)	1.076 (0.964 - 1.186)	0.002 (0.001 - 0.003)	0.995 (0.931 - 1.063)
CR30d_LR_c_all	0.014 (0.012 - 0.015)	0.049 (0.043 - 0.055)	0.735 (0.721 - 0.746)	1.188 (1.062 - 1.289)	0.003 (0.002 - 0.005)	0.989 (0.932 - 1.062)
CR30d_LRCR_c_all	0.014 (0.013 - 0.016)	0.047 (0.043 - 0.052)	0.741 (0.728 - 0.75)	1.097 (0.994 - 1.209)	0.002 (0.001 - 0.004)	0.991 (0.933 - 1.063)

7.2 Comparison of static and dynamic models performance at baseline

Dynamic models make predictions at all landmarks, including at baseline (LM0). A comparison between static models and dynamic models evaluated at baseline is presented in S-Figure 8. static models with administrative censoring at day 30 have been excluded as they do not have their dynamic counterpart. Dynamic models perform better at baseline in terms of discrimination (AUROC) and BSS, but worse in terms of calibration (E:O ratio, calibration slope and intercept and ECI).

7.3 ROC curves

ROC curves for each test set and each model are presented in S-Figure 9.

7.4 Precision-recall curves

Precision-recall curves for each test set and each model are presented in S-Figure 10.

7.5 Calibration curves - deciles

Using the deciles of the predicted 7 days risks on the test set, the test observations are grouped in ten groups. The average of the binary 7 day outcome within the groups is plotted against the average of predictions within the groups. Survival models with competing risks censored at the time of event (surv7d and surv30d) show overestimated predictions. The deciles calibration curves are presented in S-Figure 11.

7.6 Calibration curves - splines

The binary outcome is regressed against natural cubic splines with 6 degrees of freedom of *logit*(*model predictions*) and the resulting predictions and plotted against *expit*(*model predictions*) to obtain calibration curves for each test set. Survival models with competing risks censored at the time of event (surv7d and surv30d) show overestimated predictions. The calibration curves based on cubic splines are presented in S-Figure 12.

7.7 Decision curves

Decision analysis curves ((A. J. Vickers and Elkin 2006), (A. Vickers, Van Calster, and Steyerberg, 2019)) are shown for prediction thresholds between 0 and 6% for all test sets and all models. The net benefit of the model is plotted in grey, the net benefit for the decision to treat all in red and the net benefit for treat none in black. The decision curves are presented in S-Figure 13.

7.8 Predictions density curves

Prediction density curves for the positive class (CLABSI) and the negative class (no CLABSI) are shown in S-Figure 14 for the predictions on the test sets. Models that do not include death and discharge in

the outcome definition (binary, survival and competing risks models with zero weights for death and discharge in the splitrule) display a bimodal distribution of the predicted risks.

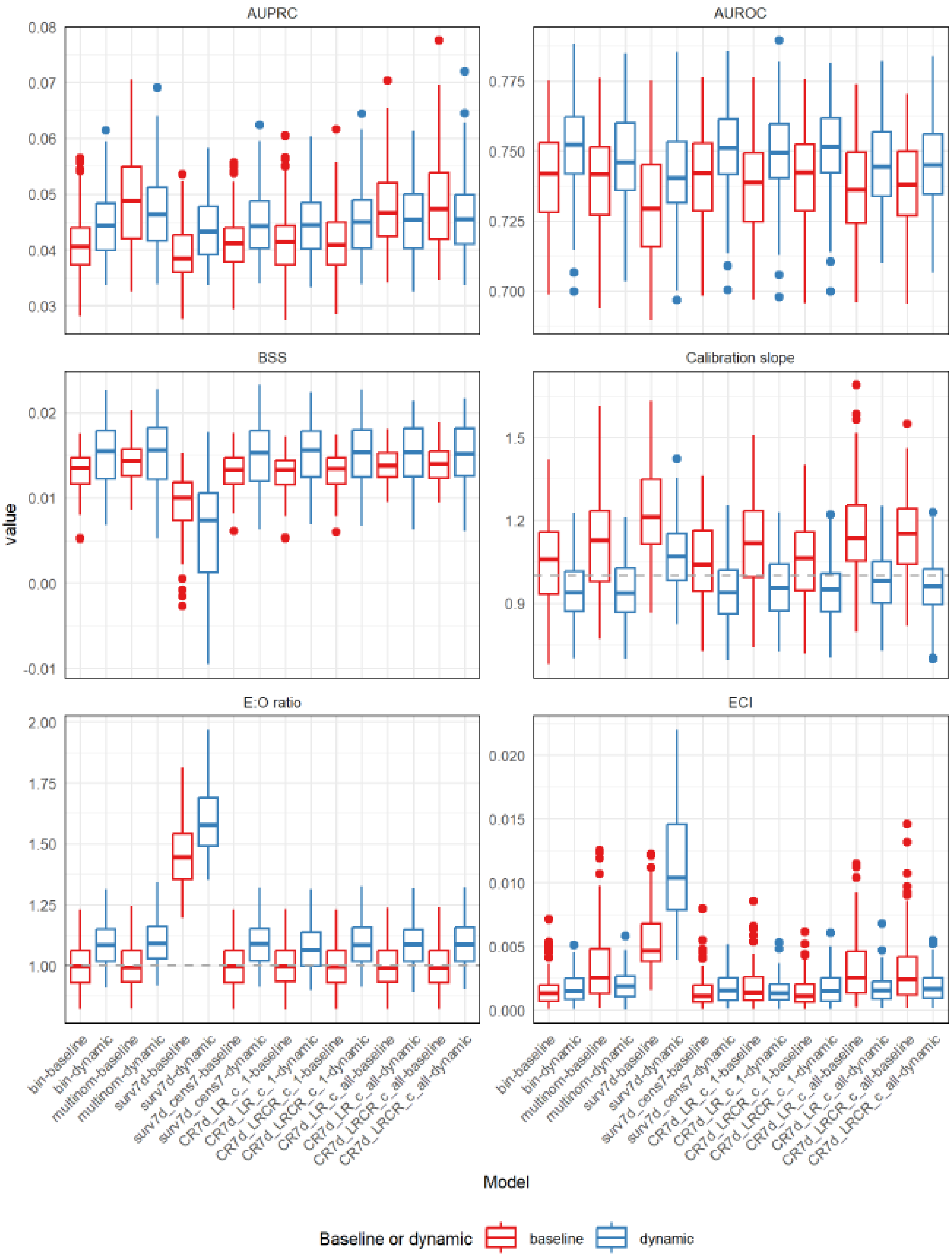

S-Figure 8: Prediction performance for static (baseline) and dynamic models at baseline (LM 0)

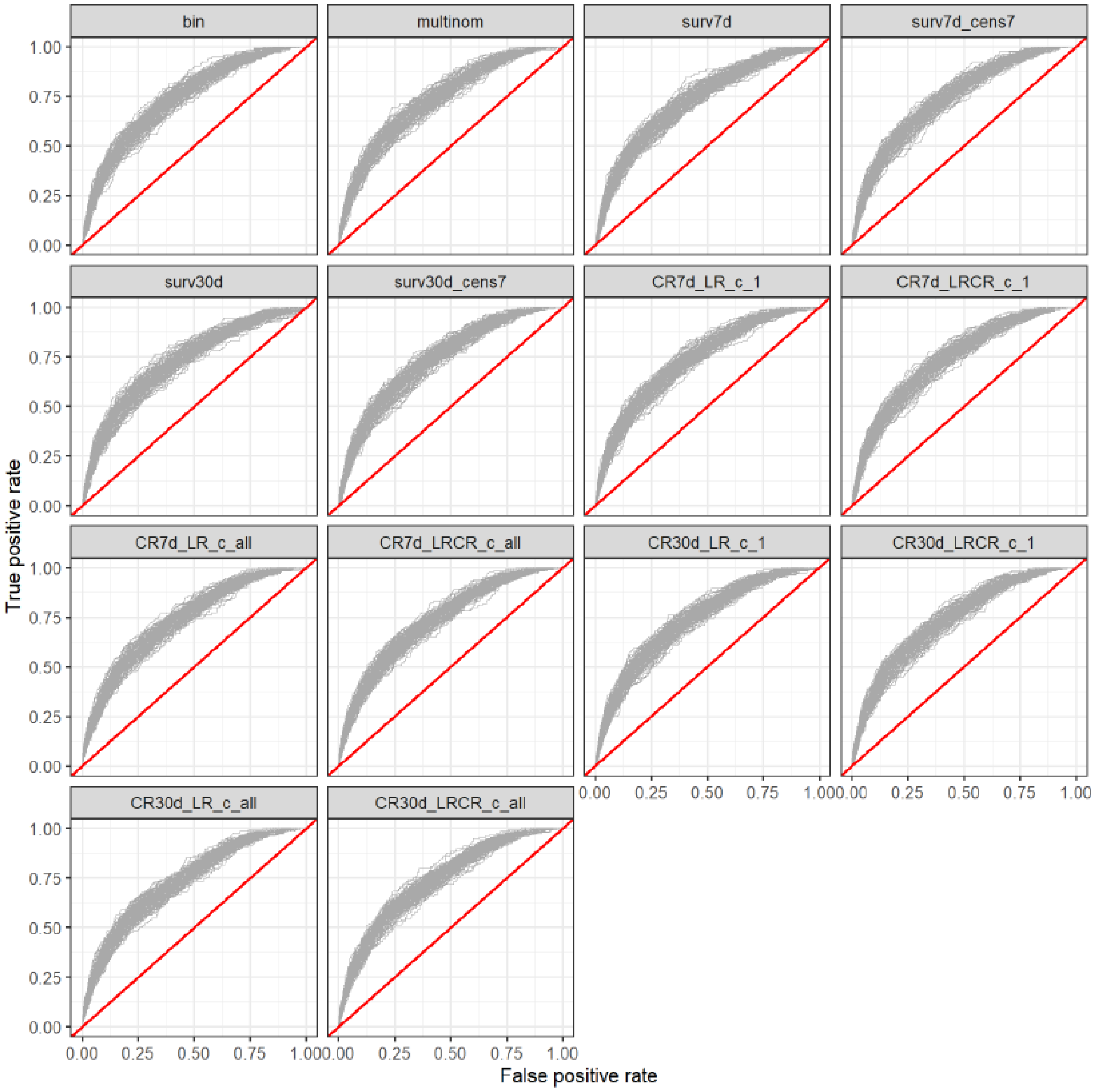

S-Figure 9: ROC curves for static models

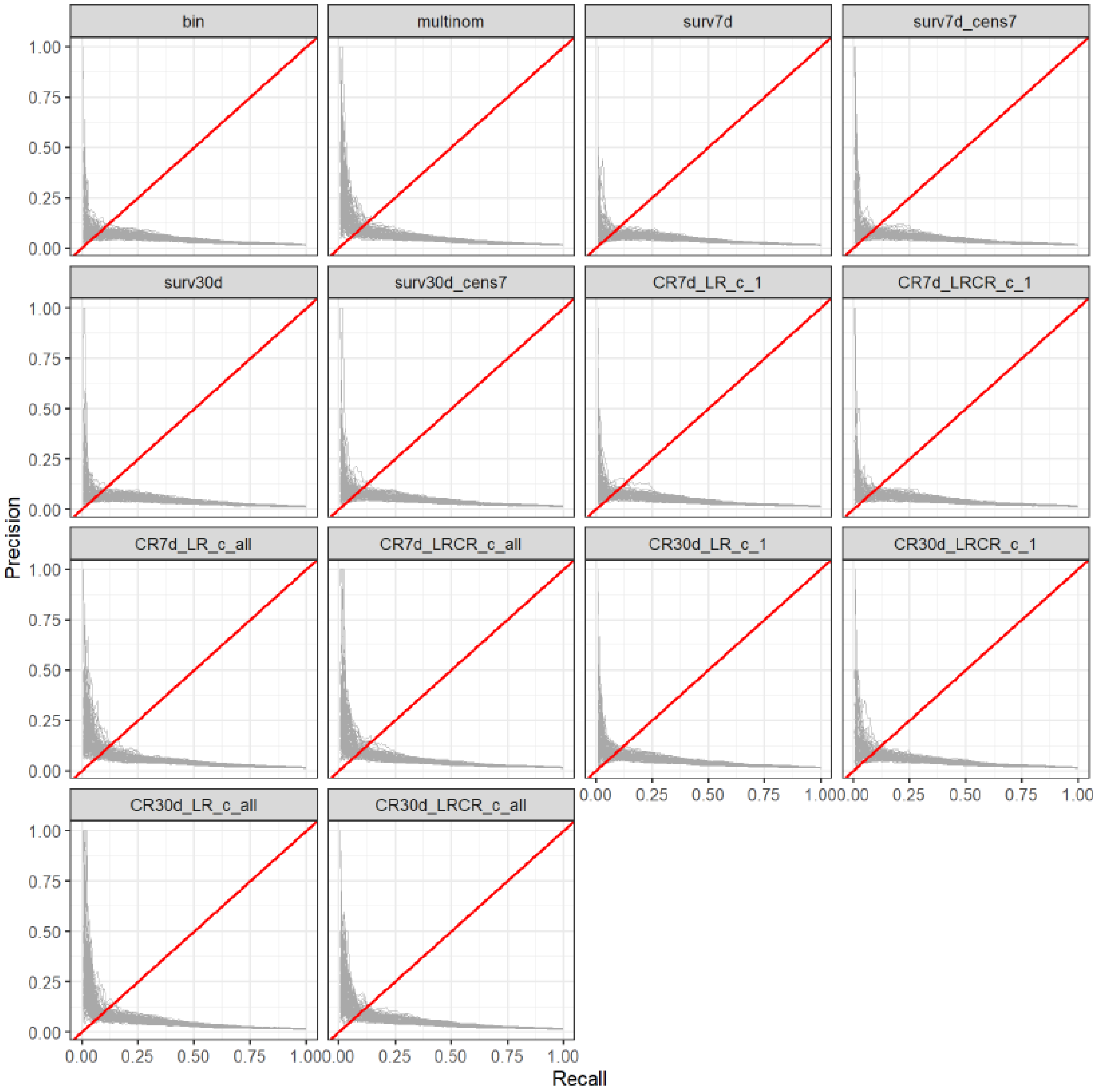

S-Figure 10: Precision-recall curves for static models

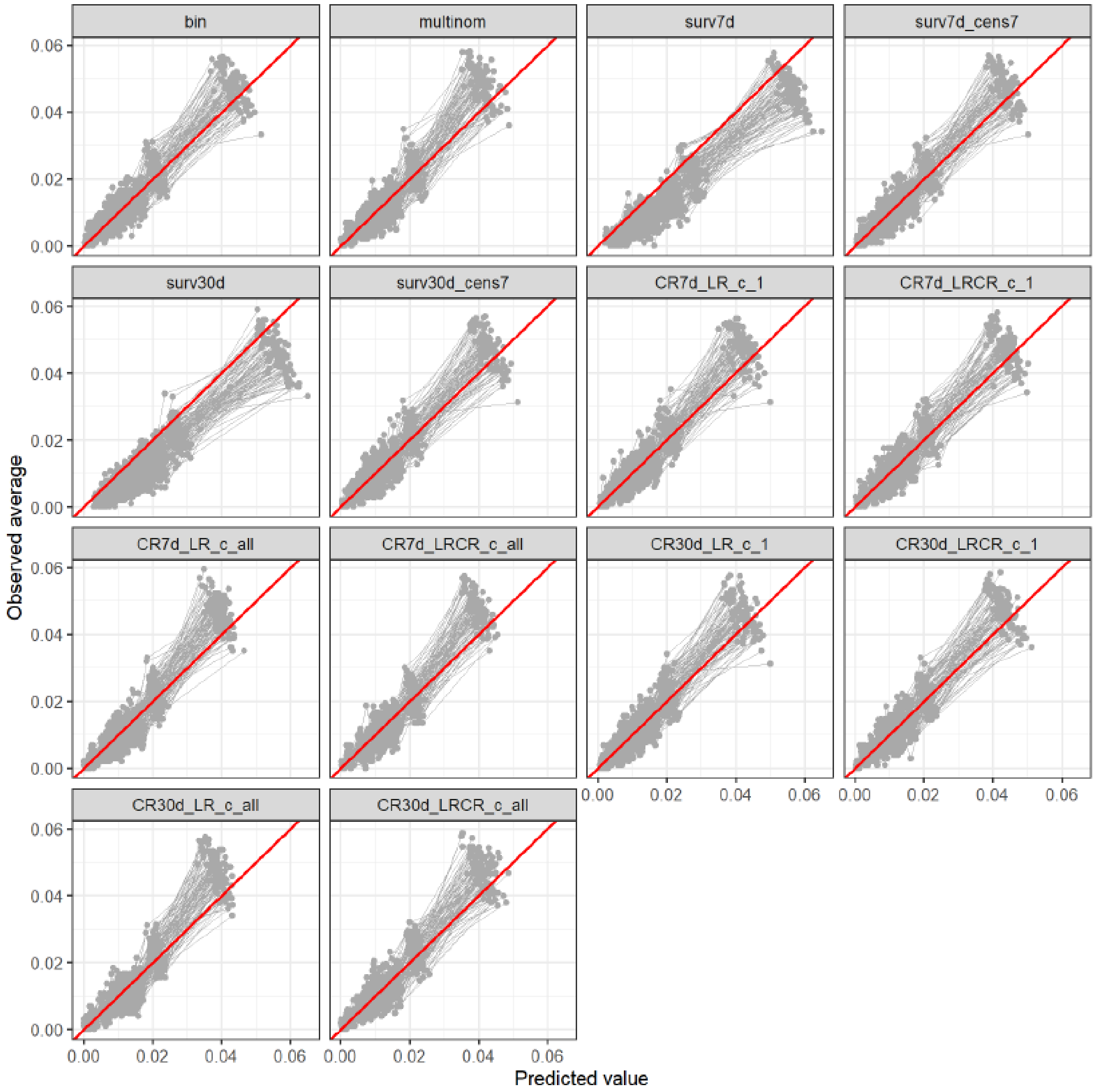

S-Figure 11: Calibration curves (deciles) for static models

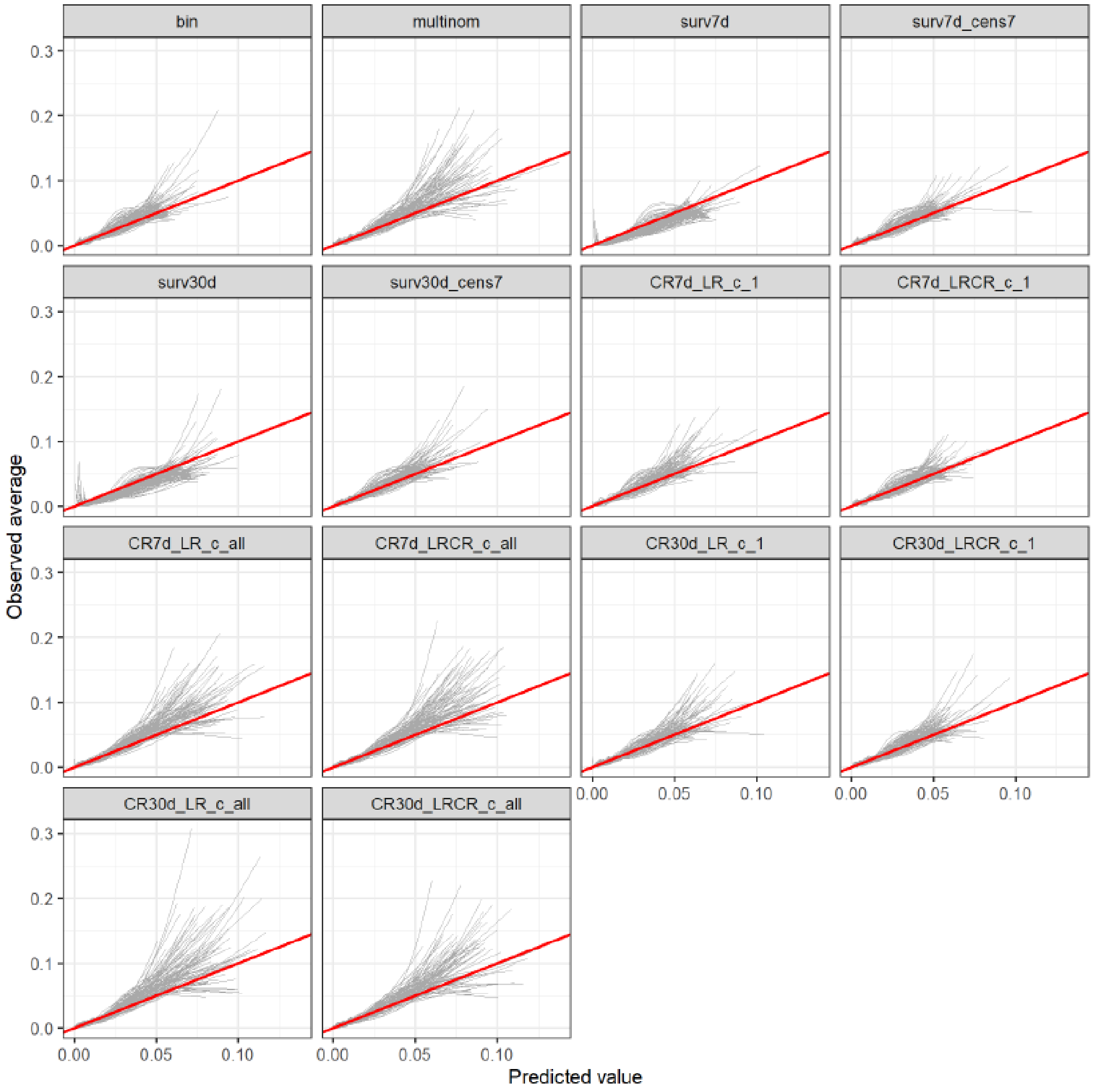

S-Figure 12: Calibration curves (splines) for static models

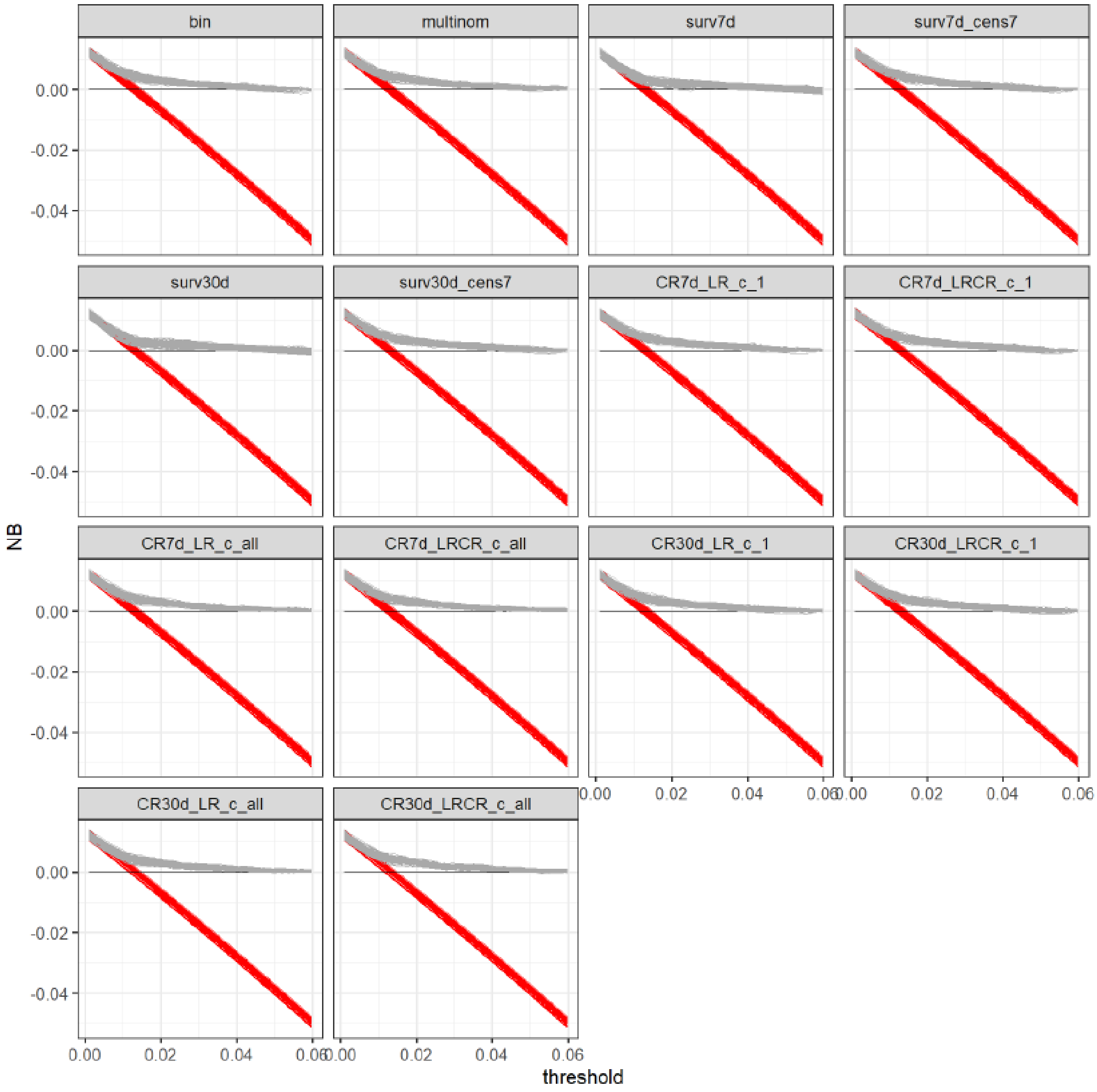

S-Figure 13: Decision curves for static models

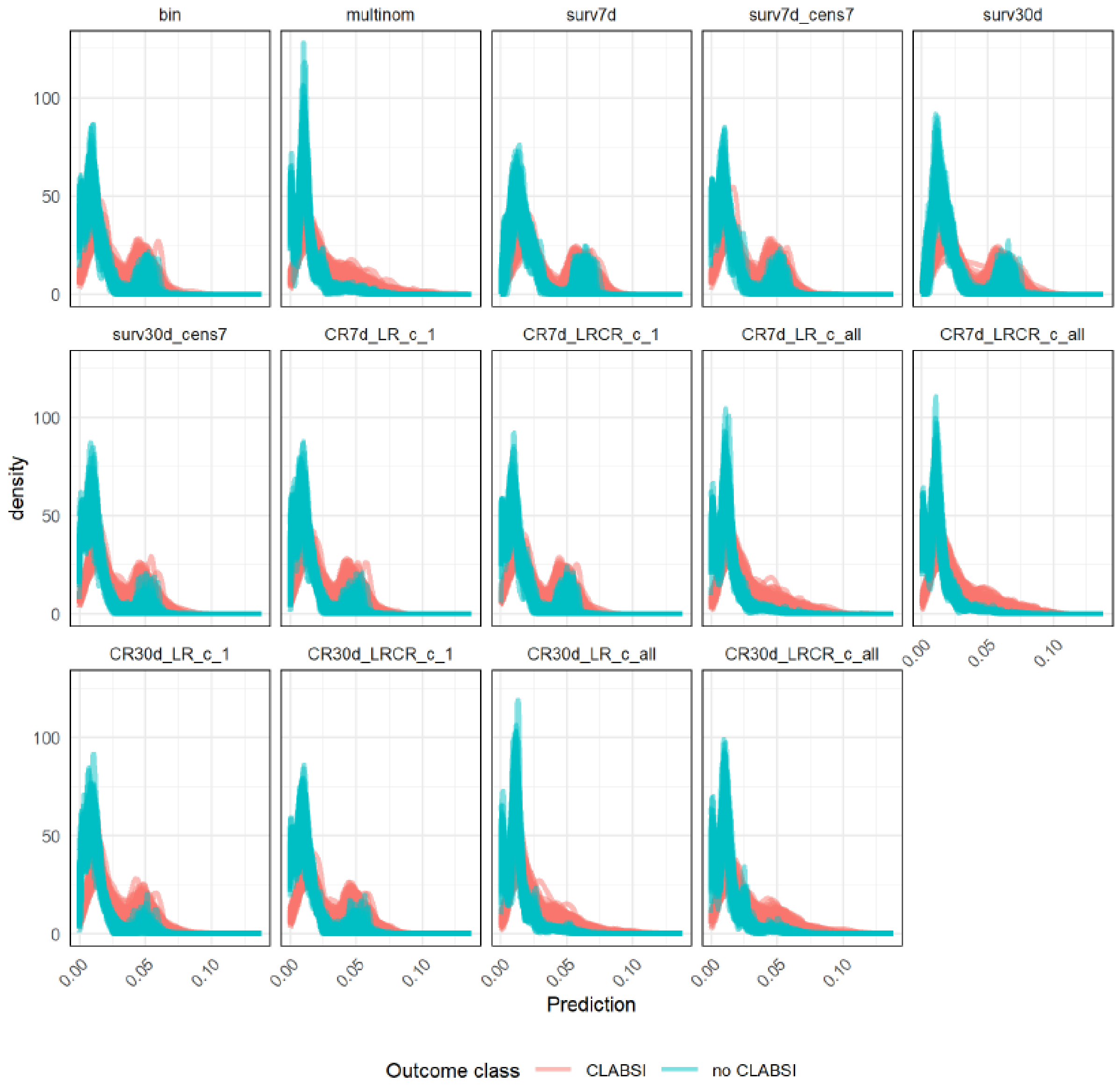

S-Figure 14: Density curves of predicted risks by outcome for static models

7.9 Tuned hyperparameters

The tuned hyperparameters for the static models (mtry and nodesize) are shown in S-Figure 15. The models using all events in the outcome definition (multinomial and CR models weighting all causes) have tuned nodesizes lower than the other models, indicating a tendency to use more splits in building the trees to achieve the best results for minimizing the binary logloss (all models are tuned for the same metric, based on the binary outcome). As each split is optimizing a loss function over multiple levels of the outcome, it might become less efficient to optimize for the binary outcome, therefore requiring more splits. These models also display a slightly larger ECI; the slight miscalibration might be due to the fact that less observations are left in the final nodes.

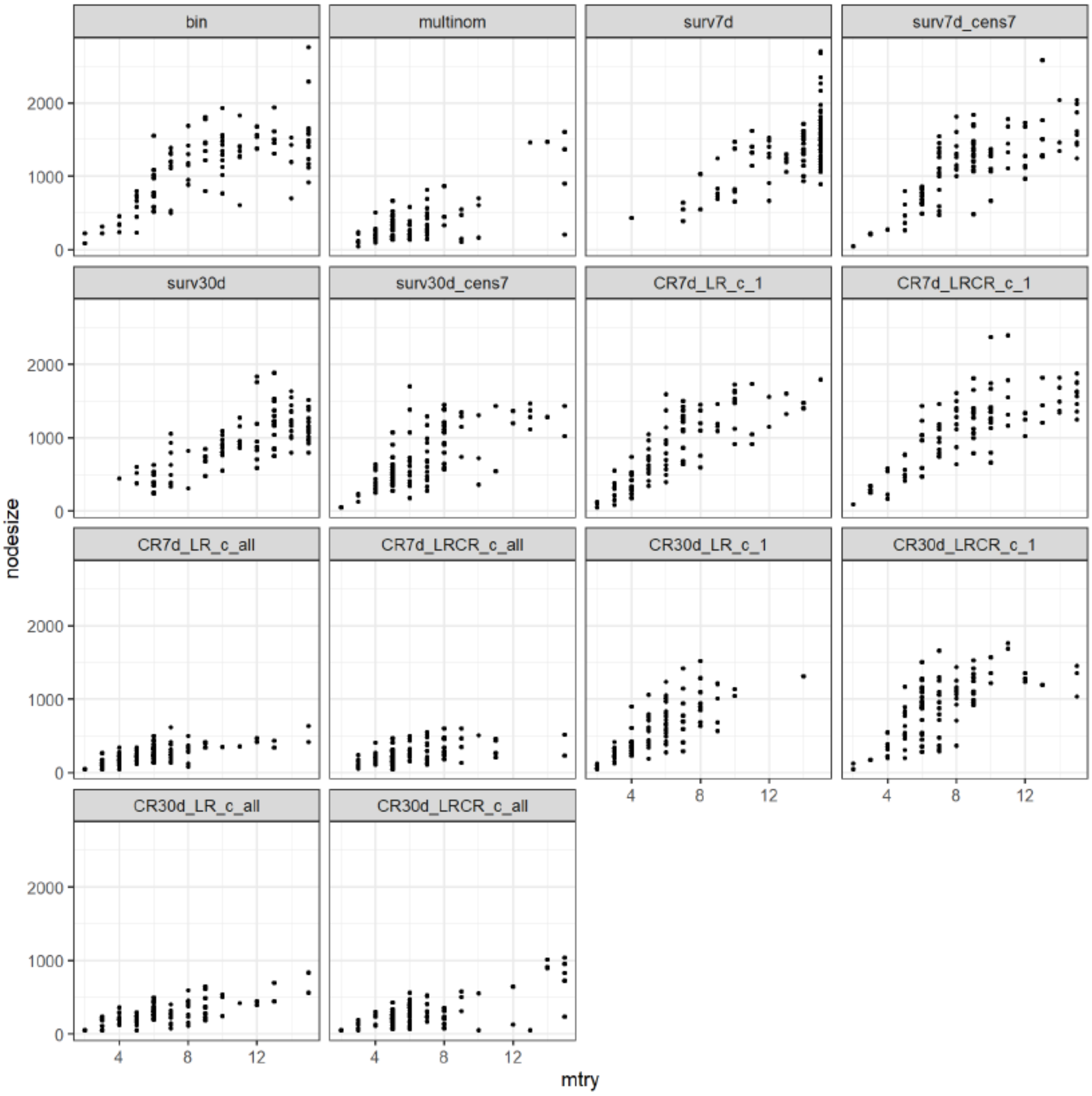

S-Figure 15: Tuned hyperparameters

7.10 Variable importance

The minimal depth of the maximal subtree is used as a variable importance metric (Ishwaran et al. 2021), which is the depth in a tree on which the first split is made on a variable *v*, averaged over all trees in the forest. The lowest possible value is 0 (root node split). The minimal depth of the maximal subtree is presented in S-Figure 16. A guiding line has been added to the plot on value 2, an aleatory choice to guide the focus on most important variables.

Models using multiple levels of the outcome put less weight on the TPN variable (splits tend to made lower down in the trees) and more weight (splits closer to the root) on variables like: ICU, antibacterials, antineoplastic agents (chemotherapy), CRP and port-catheter. Presumably, these variables could play a role in predicting discharge or death.

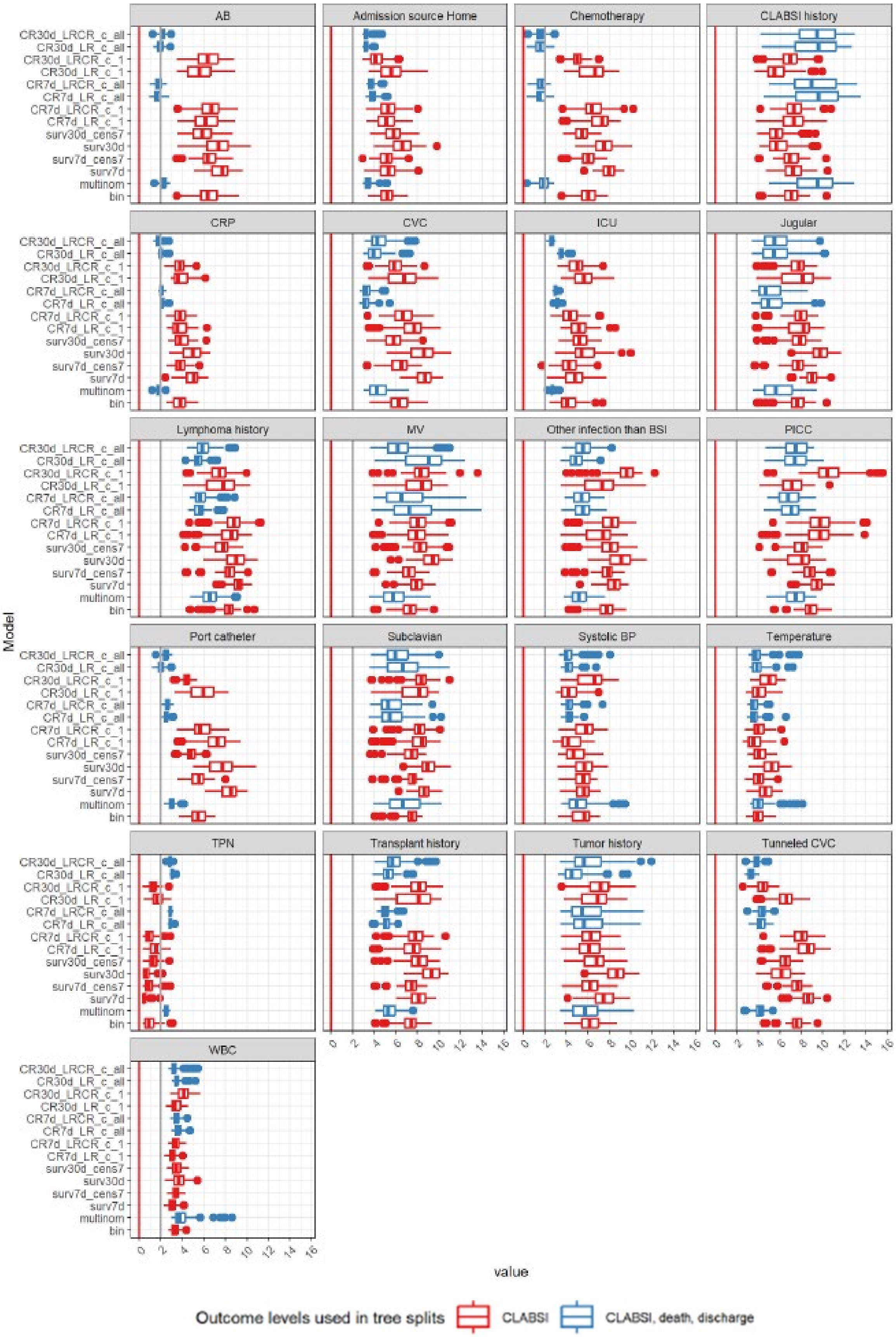

S-Figure 16: Variable importance

Supplementary material 8 - Additional performance evaluation – dynamic models

8.1 Number of CLABSI events and prevalence at each landmark

The number of CLABSI events and prevalence at each landmark over all test sets are presented in the S-Figures 17 and 18. The number of CLABSI events and prevalence at each landmark in train sets will be proportional, as the train/test splits have been performed by random sampling with a train:test ratio of 2:1.

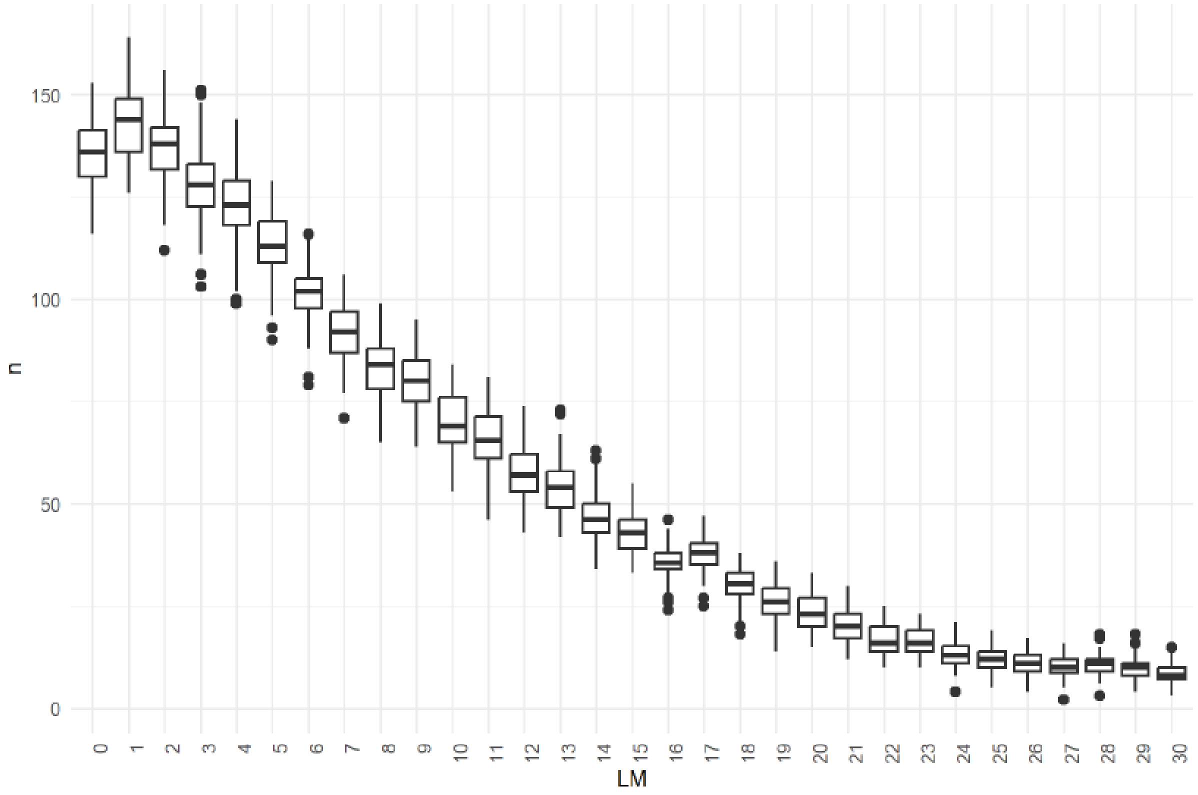

S-Figure 17: Number of CLABSI events at each landmark over all test sets

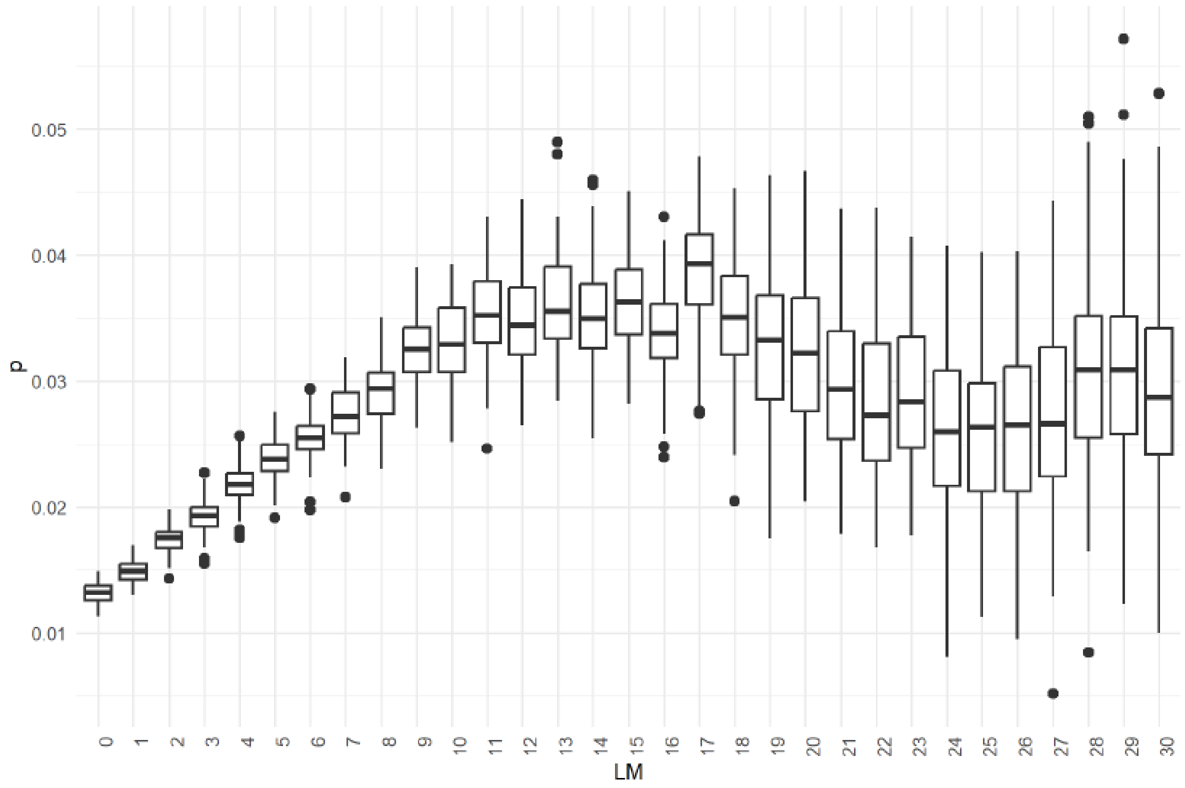

S-Figure 18: CLABSI event prevalence at each landmark over all test sets

8.2 Time-dependent metrics table

The dynamic model performance metrics per LM are presented in S-Table 8.

S-Table 8: Time-dependent metrics table

Model	LM	BSS	AUPRC	AUROC	Calibration slope	ECI	E:O ratio
bin	0	0.015 (0.012 - 0.018)	0.044 (0.04 - 0.048)	0.752 (0.742 - 0.762)	0.938 (0.871 - 1.015)	0.001 (0.001 - 0.002)	1.084 (1.019 - 1.15)
bin	1	0.021 (0.019 - 0.024)	0.056 (0.052 - 0.062)	0.763 (0.753 - 0.775)	1.033 (0.941 - 1.111)	0.002 (0.001 - 0.003)	1.065 (1.012 - 1.146)
bin	2	0.023 (0.02 - 0.026)	0.062 (0.057 - 0.07)	0.764 (0.753 - 0.777)	1.063 (0.965 - 1.169)	0.002 (0.001 - 0.004)	1.012 (0.963 - 1.067)

S-Table 8: Time-dependent metrics table *(continued)*

Model	LM	BSS	AUPRC	AUROC	Calibration slope	ECI	E:O ratio
bin	3	0.024 (0.021 - 0.027)	0.066 (0.061 - 0.074)	0.759 (0.747 - 0.775)	1.041 (0.953 - 1.137)	0.003 (0.002 - 0.004)	0.991 (0.94 - 1.045)
bin	4	0.029 (0.026 - 0.032)	0.078 (0.071 - 0.089)	0.771 (0.759 - 0.787)	1.082 (0.974 - 1.214)	0.005 (0.002 - 0.009)	0.961 (0.904 - 1.005)
bin	5	0.027 (0.024 - 0.029)	0.073 (0.068 - 0.081)	0.775 (0.762 - 0.787)	1.096 (1.006 - 1.196)	0.004 (0.003 - 0.006)	0.955 (0.891 - 1.009)
bin	6	0.024 (0.021 - 0.028)	0.07 (0.065 - 0.079)	0.757 (0.743 - 0.769)	1.043 (0.947 - 1.139)	0.005 (0.002 - 0.007)	0.973 (0.908 - 1.021)
bin	7	0.024 (0.02 - 0.027)	0.074 (0.066 - 0.079)	0.754 (0.743 - 0.771)	1.063 (0.954 - 1.198)	0.005 (0.003 - 0.008)	0.996 (0.907 - 1.049)
bin	8	0.025 (0.022 - 0.028)	0.077 (0.07 - 0.085)	0.756 (0.742 - 0.771)	1.083 (0.981 - 1.213)	0.006 (0.004 - 0.009)	0.972 (0.909 - 1.061)
bin	9	0.03 (0.026 - 0.033)	0.091 (0.083 - 0.1)	0.757 (0.744 - 0.773)	1.135 (1.004 - 1.261)	0.01 (0.006 - 0.018)	0.918 (0.855 - 0.994)
bin	10	0.025 (0.02 - 0.029)	0.082 (0.075 - 0.091)	0.741 (0.726 - 0.759)	1.041 (0.919 - 1.19)	0.01 (0.005 - 0.016)	0.941 (0.869 - 1.038)
bin	11	0.026 (0.021 - 0.03)	0.086 (0.077 - 0.097)	0.743 (0.727 - 0.758)	1.054 (0.93 - 1.2)	0.012 (0.006 - 0.021)	0.93 (0.834 - 0.989)
bin	12	0.024 (0.019 - 0.028)	0.083 (0.073 - 0.093)	0.733 (0.709 - 0.75)	1.002 (0.846 - 1.144)	0.01 (0.005 - 0.018)	0.954 (0.867 - 1.043)
bin	13	0.022 (0.017 - 0.027)	0.085 (0.072 - 0.098)	0.726 (0.7 - 0.74)	1 (0.793 - 1.127)	0.012 (0.007 - 0.021)	0.941 (0.85 - 1.029)
bin	14	0.023 (0.018 - 0.027)	0.085 (0.076 - 0.1)	0.732 (0.708 - 0.745)	1.038 (0.817 - 1.183)	0.011 (0.006 - 0.019)	0.988 (0.902 - 1.068)
bin	15	0.025 (0.022 - 0.028)	0.093 (0.083 - 0.104)	0.731 (0.718 - 0.755)	1.082 (0.949 - 1.271)	0.014 (0.01 - 0.022)	0.967 (0.884 - 1.058)
bin	16	0.025 (0.021 - 0.028)	0.089 (0.078 - 0.099)	0.739 (0.72 - 0.759)	1.106 (0.938 - 1.328)	0.014 (0.009 - 0.02)	1.052 (0.953 - 1.119)
bin	17	0.022 (0.018 - 0.026)	0.103 (0.085 - 0.122)	0.718 (0.698 - 0.739)	1.007 (0.884 - 1.215)	0.019 (0.01 - 0.03)	0.905 (0.831 - 1.004)
bin	18	0.018 (0.013 - 0.023)	0.083 (0.069 - 0.096)	0.712 (0.681 - 0.738)	0.971 (0.785 - 1.218)	0.015 (0.01 - 0.025)	1.003 (0.908 - 1.12)
bin	19	0.014 (0.009 - 0.021)	0.07 (0.06 - 0.084)	0.711 (0.683 - 0.729)	1 (0.839 - 1.192)	0.018 (0.013 - 0.028)	1.042 (0.937 - 1.228)

S-Table 8: Time-dependent metrics table *(continued)*

Model	LM	BSS	AUPRC	AUROC	Calibration slope	ECI	E:O ratio
bin	20	0.011 (0.005 - 0.016)	0.062 (0.053 - 0.075)	0.695 (0.666 - 0.723)	0.973 (0.834 - 1.184)	0.018 (0.011 - 0.028)	1.077 (0.93 - 1.29)
bin	21	0.01 (0.002 - 0.016)	0.062 (0.048 - 0.076)	0.695 (0.661 - 0.721)	1.027 (0.801 - 1.257)	0.021 (0.012 - 0.032)	1.185 (0.97 - 1.373)
bin	22	0.004 (-0.004 - 0.012)	0.046 (0.039 - 0.059)	0.684 (0.652 - 0.707)	1.03 (0.801 - 1.243)	0.028 (0.015 - 0.042)	1.252 (1.016 - 1.517)
bin	23	0.007 (-0.001 - 0.013)	0.051 (0.039 - 0.079)	0.677 (0.648 - 0.709)	1.043 (0.835 - 1.216)	0.032 (0.019 - 0.048)	1.155 (0.977 - 1.393)
bin	24	0.008 (-0.004 - 0.016)	0.048 (0.036 - 0.067)	0.694 (0.661 - 0.729)	1.142 (0.933 - 1.474)	0.03 (0.018 - 0.044)	1.288 (1.057 - 1.583)
bin	25	0.008 (-0.005 - 0.016)	0.049 (0.038 - 0.073)	0.693 (0.665 - 0.731)	1.095 (0.869 - 1.412)	0.03 (0.021 - 0.042)	1.271 (1.077 - 1.566)
bin	26	0.006 (-0.008 - 0.013)	0.047 (0.037 - 0.07)	0.684 (0.644 - 0.707)	1.055 (0.842 - 1.329)	0.041 (0.027 - 0.061)	1.248 (1.042 - 1.592)
bin	27	-0.002 (-0.014 - 0.006)	0.038 (0.029 - 0.048)	0.642 (0.611 - 0.673)	0.819 (0.64 - 1.035)	0.046 (0.03 - 0.062)	1.287 (1.028 - 1.601)
bin	28	0.003 (-0.004 - 0.01)	0.044 (0.035 - 0.053)	0.662 (0.622 - 0.693)	0.914 (0.701 - 1.185)	0.049 (0.035 - 0.07)	1.126 (0.977 - 1.36)
bin	29	0.008 (-0.003 - 0.013)	0.046 (0.038 - 0.058)	0.684 (0.65 - 0.713)	1.134 (0.864 - 1.365)	0.046 (0.033 - 0.072)	1.173 (0.976 - 1.377)
bin	30	0.014 (0.002 - 0.021)	0.055 (0.042 - 0.069)	0.736 (0.7 - 0.772)	1.568 (1.183 - 2.016)	0.048 (0.029 - 0.064)	1.218 (0.99 - 1.463)
multinom	0	0.016 (0.012 - 0.018)	0.046 (0.042 - 0.051)	0.746 (0.736 - 0.76)	0.936 (0.866 - 1.028)	0.002 (0.001 - 0.003)	1.09 (1.028 - 1.161)
multinom	1	0.021 (0.018 - 0.024)	0.056 (0.052 - 0.061)	0.763 (0.752 - 0.772)	1.022 (0.935 - 1.088)	0.002 (0.001 - 0.003)	1.077 (1.019 - 1.15)
multinom	2	0.023 (0.02 - 0.026)	0.062 (0.057 - 0.068)	0.762 (0.751 - 0.773)	1.056 (0.977 - 1.135)	0.002 (0.001 - 0.003)	1.047 (0.992 - 1.102)
multinom	3	0.025 (0.021 - 0.028)	0.068 (0.062 - 0.075)	0.76 (0.747 - 0.777)	1.031 (0.956 - 1.145)	0.003 (0.002 - 0.005)	1.019 (0.98 - 1.083)
multinom	4	0.028 (0.025 - 0.032)	0.076 (0.07 - 0.084)	0.771 (0.757 - 0.782)	1.054 (0.986 - 1.156)	0.003 (0.002 - 0.007)	0.98 (0.929 - 1.025)
multinom	5	0.026 (0.022 - 0.029)	0.072 (0.066 - 0.08)	0.772 (0.761 - 0.786)	1.035 (0.976 - 1.158)	0.004 (0.003 - 0.007)	0.954 (0.896 - 1.01)

S-Table 8: Time-dependent metrics table *(continued)*

Model	LM	BSS	AUPRC	AUROC	Calibration slope	ECI	E:O ratio
multinom	6	0.022 (0.019 - 0.026)	0.067 (0.061 - 0.075)	0.751 (0.736 - 0.763)	0.961 (0.903 - 1.064)	0.005 (0.003 - 0.008)	0.96 (0.9 - 1.014)
multinom	7	0.022 (0.018 - 0.025)	0.069 (0.062 - 0.075)	0.753 (0.739 - 0.769)	1.012 (0.919 - 1.118)	0.007 (0.004 - 0.01)	0.977 (0.9 - 1.042)
multinom	8	0.022 (0.02 - 0.026)	0.073 (0.066 - 0.081)	0.749 (0.734 - 0.763)	1.03 (0.928 - 1.15)	0.007 (0.004 - 0.011)	0.962 (0.906 - 1.039)
multinom	9	0.028 (0.024 - 0.031)	0.087 (0.079 - 0.097)	0.756 (0.74 - 0.768)	1.123 (0.996 - 1.226)	0.01 (0.005 - 0.015)	0.913 (0.865 - 0.988)
multinom	10	0.024 (0.021 - 0.029)	0.082 (0.073 - 0.093)	0.743 (0.727 - 0.758)	1.078 (0.94 - 1.183)	0.011 (0.006 - 0.017)	0.949 (0.873 - 1.038)
multinom	11	0.025 (0.02 - 0.03)	0.086 (0.076 - 0.095)	0.744 (0.728 - 0.763)	1.08 (0.969 - 1.226)	0.012 (0.008 - 0.02)	0.929 (0.843 - 0.989)
multinom	12	0.024 (0.019 - 0.028)	0.084 (0.074 - 0.098)	0.736 (0.712 - 0.75)	1.04 (0.902 - 1.176)	0.011 (0.007 - 0.02)	0.957 (0.88 - 1.038)
multinom	13	0.023 (0.017 - 0.028)	0.09 (0.078 - 0.103)	0.724 (0.7 - 0.74)	1.062 (0.862 - 1.177)	0.012 (0.007 - 0.021)	0.937 (0.843 - 1.014)
multinom	14	0.025 (0.02 - 0.029)	0.09 (0.079 - 0.108)	0.73 (0.71 - 0.745)	1.124 (0.939 - 1.248)	0.013 (0.008 - 0.023)	0.99 (0.897 - 1.072)
multinom	15	0.027 (0.023 - 0.031)	0.106 (0.089 - 0.125)	0.734 (0.714 - 0.747)	1.152 (0.989 - 1.303)	0.022 (0.012 - 0.031)	0.963 (0.878 - 1.054)
multinom	16	0.027 (0.023 - 0.029)	0.1 (0.086 - 0.115)	0.742 (0.72 - 0.757)	1.187 (1.031 - 1.357)	0.018 (0.011 - 0.027)	1.055 (0.948 - 1.11)
multinom	17	0.022 (0.018 - 0.026)	0.097 (0.084 - 0.113)	0.716 (0.696 - 0.734)	1.014 (0.858 - 1.193)	0.018 (0.012 - 0.031)	0.893 (0.83 - 0.984)
multinom	18	0.019 (0.013 - 0.024)	0.085 (0.075 - 0.099)	0.707 (0.678 - 0.738)	0.991 (0.813 - 1.243)	0.019 (0.011 - 0.028)	0.994 (0.913 - 1.098)
multinom	19	0.014 (0.009 - 0.02)	0.072 (0.06 - 0.082)	0.707 (0.677 - 0.729)	0.992 (0.847 - 1.207)	0.019 (0.011 - 0.028)	1.034 (0.938 - 1.182)
multinom	20	0.012 (0.006 - 0.017)	0.064 (0.055 - 0.079)	0.699 (0.666 - 0.727)	0.946 (0.814 - 1.18)	0.021 (0.013 - 0.03)	1.064 (0.914 - 1.234)
multinom	21	0.009 (0.001 - 0.015)	0.06 (0.046 - 0.073)	0.69 (0.656 - 0.72)	0.943 (0.745 - 1.152)	0.02 (0.015 - 0.03)	1.163 (0.955 - 1.332)
multinom	22	0.004 (-0.003 - 0.013)	0.048 (0.039 - 0.065)	0.686 (0.653 - 0.711)	0.985 (0.76 - 1.151)	0.029 (0.018 - 0.041)	1.212 (1.011 - 1.447)

S-Table 8: Time-dependent metrics table *(continued)*

Model	LM	BSS	AUPRC	AUROC	Calibration slope	ECI	E:O ratio
multinom	23	0.004 (-0.003 - 0.01)	0.046 (0.036 - 0.063)	0.668 (0.643 - 0.703)	0.918 (0.764 - 1.11)	0.036 (0.025 - 0.053)	1.143 (0.95 - 1.372)
multinom	24	0.007 (-0.006 - 0.015)	0.047 (0.035 - 0.065)	0.699 (0.669 - 0.734)	1.108 (0.879 - 1.37)	0.034 (0.021 - 0.051)	1.249 (1.025 - 1.513)
multinom	25	0.007 (-0.007 - 0.015)	0.047 (0.034 - 0.073)	0.692 (0.664 - 0.726)	1.058 (0.779 - 1.312)	0.033 (0.022 - 0.05)	1.21 (1.049 - 1.538)
multinom	26	0.008 (-0.006 - 0.015)	0.051 (0.034 - 0.07)	0.688 (0.643 - 0.718)	0.994 (0.78 - 1.273)	0.041 (0.025 - 0.062)	1.193 (0.996 - 1.527)
multinom	27	0 (-0.009 - 0.007)	0.042 (0.031 - 0.051)	0.654 (0.618 - 0.686)	0.806 (0.634 - 1.078)	0.045 (0.026 - 0.06)	1.185 (0.962 - 1.496)
multinom	28	0.004 (-0.004 - 0.009)	0.045 (0.036 - 0.055)	0.66 (0.631 - 0.69)	0.862 (0.649 - 1.087)	0.044 (0.03 - 0.066)	1.039 (0.912 - 1.265)
multinom	29	0.009 (0.001 - 0.017)	0.053 (0.039 - 0.068)	0.701 (0.669 - 0.734)	1.088 (0.84 - 1.438)	0.042 (0.027 - 0.06)	1.07 (0.905 - 1.271)
multinom	30	0.011 (0.001 - 0.02)	0.053 (0.041 - 0.073)	0.722 (0.686 - 0.764)	1.285 (0.957 - 1.658)	0.041 (0.029 - 0.058)	1.088 (0.922 - 1.307)
surv7d	0	0.007 (0.001 - 0.011)	0.043 (0.039 - 0.048)	0.74 (0.732 - 0.753)	1.069 (0.982 - 1.152)	0.01 (0.008 - 0.015)	1.576 (1.49 - 1.688)
surv7d	1	0.014 (0.01 - 0.017)	0.056 (0.051 - 0.062)	0.751 (0.744 - 0.766)	1.155 (1.068 - 1.255)	0.01 (0.007 - 0.013)	1.529 (1.457 - 1.653)
surv7d	2	0.018 (0.013 - 0.022)	0.061 (0.056 - 0.068)	0.754 (0.741 - 0.764)	1.174 (1.083 - 1.259)	0.009 (0.007 - 0.013)	1.429 (1.363 - 1.526)
surv7d	3	0.019 (0.013 - 0.024)	0.064 (0.059 - 0.071)	0.752 (0.74 - 0.765)	1.159 (1.063 - 1.267)	0.011 (0.008 - 0.015)	1.406 (1.338 - 1.504)
surv7d	4	0.025 (0.02 - 0.03)	0.076 (0.069 - 0.087)	0.763 (0.748 - 0.78)	1.2 (1.102 - 1.331)	0.012 (0.008 - 0.016)	1.365 (1.291 - 1.444)
surv7d	5	0.023 (0.018 - 0.028)	0.075 (0.068 - 0.083)	0.768 (0.755 - 0.78)	1.189 (1.092 - 1.285)	0.012 (0.008 - 0.017)	1.347 (1.261 - 1.413)
surv7d	6	0.019 (0.015 - 0.026)	0.072 (0.066 - 0.082)	0.751 (0.738 - 0.763)	1.111 (1.005 - 1.205)	0.015 (0.01 - 0.02)	1.351 (1.271 - 1.43)
surv7d	7	0.019 (0.011 - 0.025)	0.074 (0.065 - 0.082)	0.75 (0.736 - 0.764)	1.099 (0.989 - 1.232)	0.018 (0.012 - 0.026)	1.363 (1.233 - 1.445)
surv7d	8	0.021 (0.015 - 0.028)	0.08 (0.07 - 0.088)	0.757 (0.741 - 0.77)	1.13 (1.037 - 1.262)	0.018 (0.012 - 0.025)	1.317 (1.224 - 1.412)

S-Table 8: Time-dependent metrics table *(continued)*

Model	LM	BSS	AUPRC	AUROC	Calibration slope	ECI	E:O ratio
surv7d	9	0.03 (0.023 - 0.035)	0.094 (0.085 - 0.102)	0.757 (0.74 - 0.77)	1.155 (1.06 - 1.3)	0.014 (0.008 - 0.02)	1.204 (1.131 - 1.307)
surv7d	10	0.025 (0.018 - 0.03)	0.086 (0.076 - 0.097)	0.741 (0.726 - 0.759)	1.085 (0.976 - 1.219)	0.018 (0.011 - 0.024)	1.227 (1.114 - 1.336)
surv7d	11	0.025 (0.019 - 0.03)	0.085 (0.078 - 0.092)	0.741 (0.726 - 0.756)	1.073 (0.964 - 1.239)	0.017 (0.009 - 0.023)	1.2 (1.079 - 1.276)
surv7d	12	0.02 (0.014 - 0.026)	0.078 (0.07 - 0.088)	0.733 (0.713 - 0.747)	1.028 (0.887 - 1.153)	0.021 (0.013 - 0.029)	1.224 (1.11 - 1.338)
surv7d	13	0.02 (0.013 - 0.027)	0.08 (0.07 - 0.093)	0.724 (0.703 - 0.741)	1.012 (0.839 - 1.154)	0.02 (0.014 - 0.031)	1.193 (1.081 - 1.298)
surv7d	14	0.018 (0.009 - 0.025)	0.077 (0.068 - 0.09)	0.723 (0.705 - 0.738)	1.022 (0.846 - 1.163)	0.022 (0.015 - 0.033)	1.256 (1.145 - 1.362)
surv7d	15	0.02 (0.014 - 0.025)	0.08 (0.072 - 0.09)	0.724 (0.704 - 0.741)	1.042 (0.941 - 1.246)	0.021 (0.015 - 0.035)	1.222 (1.113 - 1.324)
surv7d	16	0.019 (0.011 - 0.024)	0.08 (0.067 - 0.09)	0.73 (0.71 - 0.748)	1.088 (0.911 - 1.297)	0.023 (0.017 - 0.038)	1.32 (1.191 - 1.4)
surv7d	17	0.019 (0.012 - 0.025)	0.086 (0.072 - 0.104)	0.706 (0.688 - 0.727)	1.018 (0.827 - 1.172)	0.02 (0.011 - 0.035)	1.126 (1.04 - 1.255)
surv7d	18	0.012 (0.004 - 0.017)	0.071 (0.062 - 0.083)	0.7 (0.679 - 0.723)	0.945 (0.788 - 1.165)	0.028 (0.017 - 0.043)	1.247 (1.123 - 1.395)
surv7d	19	0.007 (-0.002 - 0.016)	0.064 (0.055 - 0.076)	0.696 (0.671 - 0.718)	1 (0.806 - 1.147)	0.038 (0.02 - 0.053)	1.317 (1.17 - 1.541)
surv7d	20	0.003 (-0.009 - 0.014)	0.058 (0.048 - 0.071)	0.685 (0.654 - 0.713)	0.95 (0.79 - 1.18)	0.044 (0.024 - 0.059)	1.357 (1.156 - 1.614)
surv7d	21	-0.001 (-0.013 - 0.011)	0.054 (0.044 - 0.07)	0.69 (0.655 - 0.711)	0.994 (0.768 - 1.227)	0.045 (0.029 - 0.067)	1.504 (1.224 - 1.738)
surv7d	22	-0.009 (-0.026 - 0.003)	0.043 (0.035 - 0.056)	0.665 (0.638 - 0.696)	0.929 (0.808 - 1.155)	0.069 (0.04 - 0.089)	1.587 (1.285 - 1.901)
surv7d	23	-0.003 (-0.022 - 0.006)	0.046 (0.035 - 0.06)	0.671 (0.644 - 0.698)	0.97 (0.845 - 1.204)	0.054 (0.035 - 0.085)	1.491 (1.228 - 1.809)
surv7d	24	-0.006 (-0.033 - 0.007)	0.042 (0.033 - 0.056)	0.688 (0.655 - 0.716)	1.134 (0.829 - 1.325)	0.059 (0.036 - 0.091)	1.634 (1.339 - 2.033)
surv7d	25	-0.009 (-0.031 - 0.007)	0.044 (0.034 - 0.061)	0.685 (0.66 - 0.711)	1.099 (0.847 - 1.361)	0.068 (0.036 - 0.096)	1.633 (1.383 - 2.03)

S-Table 8: Time-dependent metrics table *(continued)*

Model	LM	BSS	AUPRC	AUROC	Calibration slope	ECI	E:O ratio
surv7d	26	-0.007 (-0.029 - 0.005)	0.044 (0.033 - 0.057)	0.671 (0.639 - 0.697)	1.06 (0.821 - 1.277)	0.069 (0.035 - 0.102)	1.621 (1.333 - 2.028)
surv7d	27	-0.016 (-0.038 - -0.001)	0.037 (0.029 - 0.044)	0.647 (0.616 - 0.678)	0.865 (0.693 - 1.047)	0.089 (0.06 - 0.113)	1.653 (1.302 - 2.044)
surv7d	28	-0.005 (-0.024 - 0.003)	0.043 (0.035 - 0.05)	0.654 (0.623 - 0.686)	0.945 (0.701 - 1.148)	0.078 (0.056 - 0.105)	1.446 (1.229 - 1.731)
surv7d	29	-0.002 (-0.021 - 0.007)	0.046 (0.036 - 0.056)	0.676 (0.635 - 0.7)	1.061 (0.866 - 1.366)	0.073 (0.049 - 0.113)	1.465 (1.224 - 1.764)
surv7d	30	0 (-0.019 - 0.015)	0.05 (0.038 - 0.064)	0.708 (0.661 - 0.733)	1.366 (0.995 - 1.772)	0.071 (0.047 - 0.096)	1.541 (1.272 - 1.841)
surv7d_cens7	0	0.015 (0.012 - 0.018)	0.044 (0.04 - 0.049)	0.751 (0.742 - 0.761)	0.938 (0.86 - 1.019)	0.002 (0.001 - 0.003)	1.088 (1.021 - 1.153)
surv7d_cens7	1	0.021 (0.019 - 0.023)	0.057 (0.052 - 0.061)	0.763 (0.752 - 0.776)	1.02 (0.945 - 1.104)	0.002 (0.001 - 0.003)	1.069 (1.02 - 1.148)
surv7d_cens7	2	0.023 (0.02 - 0.026)	0.062 (0.058 - 0.071)	0.763 (0.753 - 0.778)	1.069 (0.969 - 1.165)	0.002 (0.001 - 0.004)	1.015 (0.963 - 1.078)
surv7d_cens7	3	0.024 (0.021 - 0.027)	0.065 (0.061 - 0.073)	0.76 (0.748 - 0.774)	1.037 (0.955 - 1.135)	0.003 (0.002 - 0.005)	0.991 (0.945 - 1.053)
surv7d_cens7	4	0.029 (0.026 - 0.032)	0.078 (0.07 - 0.087)	0.77 (0.759 - 0.785)	1.079 (0.983 - 1.182)	0.004 (0.002 - 0.009)	0.958 (0.906 - 1.004)
surv7d_cens7	5	0.027 (0.024 - 0.029)	0.073 (0.068 - 0.082)	0.775 (0.762 - 0.787)	1.08 (1.007 - 1.192)	0.005 (0.002 - 0.007)	0.951 (0.887 - 1.005)
surv7d_cens7	6	0.024 (0.021 - 0.028)	0.07 (0.065 - 0.079)	0.756 (0.744 - 0.768)	1.034 (0.927 - 1.126)	0.005 (0.003 - 0.007)	0.968 (0.909 - 1.019)
surv7d_cens7	7	0.024 (0.02 - 0.027)	0.075 (0.066 - 0.08)	0.753 (0.742 - 0.771)	1.051 (0.956 - 1.203)	0.005 (0.003 - 0.008)	0.989 (0.903 - 1.05)
surv7d_cens7	8	0.025 (0.022 - 0.029)	0.077 (0.07 - 0.084)	0.755 (0.743 - 0.77)	1.074 (0.982 - 1.212)	0.006 (0.004 - 0.01)	0.968 (0.91 - 1.057)
surv7d_cens7	9	0.03 (0.026 - 0.033)	0.092 (0.084 - 0.099)	0.758 (0.744 - 0.774)	1.104 (0.995 - 1.276)	0.01 (0.006 - 0.017)	0.918 (0.858 - 0.99)
surv7d_cens7	10	0.025 (0.02 - 0.03)	0.083 (0.074 - 0.093)	0.739 (0.726 - 0.76)	1.028 (0.923 - 1.174)	0.011 (0.005 - 0.016)	0.941 (0.868 - 1.038)
surv7d_cens7	11	0.026 (0.021 - 0.03)	0.086 (0.078 - 0.096)	0.743 (0.728 - 0.759)	1.057 (0.927 - 1.199)	0.011 (0.006 - 0.022)	0.928 (0.84 - 0.994)

S-Table 8: Time-dependent metrics table *(continued)*

Model	LM	BSS	AUPRC	AUROC	Calibration slope	ECI	E:O ratio
surv7d_cens7	12	0.024 (0.019 - 0.028)	0.083 (0.075 - 0.093)	0.734 (0.71 - 0.75)	1.02 (0.814 - 1.169)	0.01 (0.006 - 0.02)	0.958 (0.87 - 1.047)
surv7d_cens7	13	0.023 (0.017 - 0.028)	0.086 (0.074 - 0.096)	0.727 (0.701 - 0.742)	1.002 (0.798 - 1.117)	0.012 (0.008 - 0.02)	0.941 (0.844 - 1.016)
surv7d_cens7	14	0.023 (0.018 - 0.028)	0.085 (0.076 - 0.097)	0.731 (0.71 - 0.744)	1.029 (0.854 - 1.168)	0.012 (0.006 - 0.017)	0.987 (0.9 - 1.068)
surv7d_cens7	15	0.025 (0.022 - 0.029)	0.093 (0.083 - 0.105)	0.733 (0.717 - 0.753)	1.076 (0.933 - 1.277)	0.014 (0.009 - 0.022)	0.978 (0.887 - 1.062)
surv7d_cens7	16	0.025 (0.021 - 0.029)	0.089 (0.078 - 0.099)	0.74 (0.717 - 0.762)	1.113 (0.947 - 1.32)	0.013 (0.009 - 0.021)	1.058 (0.957 - 1.121)
surv7d_cens7	17	0.022 (0.018 - 0.027)	0.104 (0.085 - 0.12)	0.718 (0.697 - 0.741)	1.021 (0.828 - 1.183)	0.018 (0.012 - 0.035)	0.903 (0.834 - 1.005)
surv7d_cens7	18	0.018 (0.013 - 0.023)	0.08 (0.069 - 0.096)	0.712 (0.679 - 0.734)	0.93 (0.761 - 1.175)	0.017 (0.009 - 0.026)	1.011 (0.907 - 1.115)
surv7d_cens7	19	0.014 (0.009 - 0.02)	0.068 (0.059 - 0.083)	0.705 (0.676 - 0.73)	0.959 (0.807 - 1.19)	0.02 (0.014 - 0.026)	1.042 (0.95 - 1.234)
surv7d_cens7	20	0.011 (0.005 - 0.016)	0.061 (0.052 - 0.075)	0.693 (0.664 - 0.72)	0.953 (0.817 - 1.175)	0.018 (0.012 - 0.033)	1.08 (0.925 - 1.289)
surv7d_cens7	21	0.01 (0.001 - 0.017)	0.063 (0.048 - 0.075)	0.697 (0.66 - 0.721)	1.02 (0.797 - 1.233)	0.021 (0.013 - 0.036)	1.185 (0.966 - 1.378)
surv7d_cens7	22	0.003 (-0.003 - 0.011)	0.045 (0.038 - 0.062)	0.682 (0.655 - 0.704)	1.05 (0.794 - 1.235)	0.027 (0.017 - 0.041)	1.243 (1.018 - 1.513)
surv7d_cens7	23	0.007 (-0.002 - 0.013)	0.051 (0.039 - 0.074)	0.678 (0.652 - 0.707)	1.042 (0.85 - 1.225)	0.033 (0.02 - 0.048)	1.157 (0.958 - 1.396)
surv7d_cens7	24	0.008 (-0.005 - 0.016)	0.048 (0.037 - 0.063)	0.7 (0.662 - 0.728)	1.19 (0.916 - 1.442)	0.031 (0.016 - 0.041)	1.28 (1.06 - 1.582)
surv7d_cens7	25	0.006 (-0.005 - 0.017)	0.05 (0.036 - 0.08)	0.694 (0.664 - 0.73)	1.102 (0.861 - 1.435)	0.029 (0.019 - 0.048)	1.286 (1.085 - 1.579)
surv7d_cens7	26	0.007 (-0.01 - 0.014)	0.047 (0.036 - 0.071)	0.68 (0.639 - 0.708)	1.081 (0.782 - 1.275)	0.043 (0.025 - 0.057)	1.274 (1.034 - 1.611)
surv7d_cens7	27	0 (-0.014 - 0.006)	0.04 (0.029 - 0.046)	0.647 (0.612 - 0.676)	0.812 (0.629 - 1.033)	0.048 (0.03 - 0.063)	1.285 (1.018 - 1.62)
surv7d_cens7	28	0.004 (-0.005 - 0.01)	0.045 (0.035 - 0.052)	0.665 (0.626 - 0.695)	0.948 (0.699 - 1.189)	0.049 (0.036 - 0.067)	1.126 (0.969 - 1.379)

S-Table 8: Time-dependent metrics table *(continued)*

Model	LM	BSS	AUPRC	AUROC	Calibration slope	ECI	E:O ratio
surv7d_cens7	29	0.008 (-0.001 - 0.013)	0.047 (0.038 - 0.058)	0.686 (0.644 - 0.724)	1.121 (0.888 - 1.446)	0.046 (0.035 - 0.073)	1.146 (0.97 - 1.384)
surv7d_cens7	30	0.015 (0.003 - 0.021)	0.054 (0.041 - 0.069)	0.736 (0.692 - 0.771)	1.528 (1.183 - 1.968)	0.044 (0.028 - 0.066)	1.221 (1.01 - 1.445)
CR7d_LR_c_1	0	0.016 (0.012 - 0.018)	0.044 (0.04 - 0.048)	0.749 (0.74 - 0.76)	0.955 (0.872 - 1.04)	0.001 (0.001 - 0.002)	1.061 (0.998 - 1.136)
CR7d_LR_c_1	1	0.021 (0.018 - 0.023)	0.056 (0.052 - 0.062)	0.761 (0.75 - 0.774)	1.037 (0.971 - 1.123)	0.002 (0.001 - 0.003)	1.061 (1.004 - 1.141)
CR7d_LR_c_1	2	0.022 (0.02 - 0.025)	0.062 (0.057 - 0.07)	0.763 (0.751 - 0.775)	1.077 (0.99 - 1.175)	0.003 (0.001 - 0.004)	1.013 (0.962 - 1.071)
CR7d_LR_c_1	3	0.024 (0.021 - 0.027)	0.066 (0.061 - 0.072)	0.759 (0.748 - 0.773)	1.081 (0.975 - 1.158)	0.003 (0.002 - 0.005)	1.005 (0.953 - 1.064)
CR7d_LR_c_1	4	0.028 (0.025 - 0.032)	0.078 (0.07 - 0.09)	0.771 (0.758 - 0.785)	1.12 (1.021 - 1.225)	0.004 (0.002 - 0.009)	0.98 (0.926 - 1.03)
CR7d_LR_c_1	5	0.027 (0.024 - 0.029)	0.073 (0.069 - 0.082)	0.774 (0.761 - 0.786)	1.114 (1.025 - 1.213)	0.005 (0.003 - 0.007)	0.973 (0.911 - 1.026)
CR7d_LR_c_1	6	0.024 (0.021 - 0.028)	0.071 (0.064 - 0.079)	0.756 (0.744 - 0.769)	1.037 (0.947 - 1.149)	0.005 (0.002 - 0.008)	0.989 (0.923 - 1.037)
CR7d_LR_c_1	7	0.024 (0.02 - 0.027)	0.074 (0.065 - 0.08)	0.753 (0.741 - 0.771)	1.042 (0.935 - 1.181)	0.005 (0.003 - 0.009)	1.003 (0.917 - 1.06)
CR7d_LR_c_1	8	0.025 (0.022 - 0.029)	0.078 (0.069 - 0.085)	0.756 (0.741 - 0.773)	1.074 (0.982 - 1.203)	0.006 (0.004 - 0.01)	0.974 (0.912 - 1.063)
CR7d_LR_c_1	9	0.031 (0.026 - 0.034)	0.092 (0.083 - 0.102)	0.759 (0.744 - 0.775)	1.1 (0.99 - 1.261)	0.011 (0.006 - 0.018)	0.914 (0.853 - 0.987)
CR7d_LR_c_1	10	0.026 (0.021 - 0.03)	0.083 (0.076 - 0.094)	0.742 (0.728 - 0.759)	1.036 (0.905 - 1.145)	0.01 (0.006 - 0.016)	0.941 (0.858 - 1.025)
CR7d_LR_c_1	11	0.027 (0.021 - 0.03)	0.084 (0.077 - 0.094)	0.745 (0.73 - 0.759)	1.048 (0.922 - 1.152)	0.011 (0.007 - 0.019)	0.927 (0.832 - 0.991)
CR7d_LR_c_1	12	0.024 (0.018 - 0.028)	0.083 (0.073 - 0.094)	0.735 (0.712 - 0.75)	0.995 (0.832 - 1.13)	0.01 (0.005 - 0.02)	0.949 (0.867 - 1.029)
CR7d_LR_c_1	13	0.023 (0.017 - 0.028)	0.084 (0.073 - 0.096)	0.727 (0.701 - 0.741)	0.964 (0.782 - 1.092)	0.012 (0.007 - 0.019)	0.942 (0.841 - 1.025)
CR7d_LR_c_1	14	0.023 (0.018 - 0.028)	0.084 (0.073 - 0.096)	0.731 (0.708 - 0.746)	1.009 (0.839 - 1.158)	0.01 (0.006 - 0.02)	0.987 (0.897 - 1.062)

S-Table 8: Time-dependent metrics table *(continued)*

Model	LM	BSS	AUPRC	AUROC	Calibration slope	ECI	E:O ratio
CR7d_LR_c_1	15	0.025 (0.021 - 0.029)	0.09 (0.082 - 0.101)	0.732 (0.712 - 0.751)	1.026 (0.909 - 1.233)	0.014 (0.007 - 0.02)	0.962 (0.88 - 1.047)
CR7d_LR_c_1	16	0.025 (0.02 - 0.028)	0.087 (0.077 - 0.097)	0.74 (0.717 - 0.758)	1.076 (0.905 - 1.27)	0.013 (0.007 - 0.019)	1.046 (0.95 - 1.112)
CR7d_LR_c_1	17	0.022 (0.018 - 0.026)	0.102 (0.085 - 0.115)	0.72 (0.698 - 0.738)	0.983 (0.811 - 1.174)	0.019 (0.01 - 0.032)	0.9 (0.825 - 0.997)
CR7d_LR_c_1	18	0.018 (0.013 - 0.023)	0.079 (0.069 - 0.094)	0.712 (0.685 - 0.733)	0.946 (0.734 - 1.173)	0.016 (0.009 - 0.026)	1.001 (0.896 - 1.112)
CR7d_LR_c_1	19	0.014 (0.009 - 0.02)	0.069 (0.059 - 0.082)	0.706 (0.68 - 0.731)	0.975 (0.818 - 1.145)	0.018 (0.012 - 0.029)	1.045 (0.927 - 1.226)
CR7d_LR_c_1	20	0.01 (0.004 - 0.015)	0.061 (0.052 - 0.075)	0.692 (0.661 - 0.72)	0.91 (0.792 - 1.108)	0.019 (0.012 - 0.03)	1.087 (0.914 - 1.281)
CR7d_LR_c_1	21	0.01 (0.001 - 0.016)	0.06 (0.047 - 0.073)	0.695 (0.659 - 0.72)	0.995 (0.763 - 1.188)	0.021 (0.013 - 0.036)	1.183 (0.958 - 1.368)
CR7d_LR_c_1	22	0.003 (-0.004 - 0.011)	0.045 (0.038 - 0.061)	0.683 (0.653 - 0.7)	0.988 (0.808 - 1.18)	0.03 (0.019 - 0.044)	1.242 (1.008 - 1.509)
CR7d_LR_c_1	23	0.006 (-0.001 - 0.012)	0.05 (0.037 - 0.076)	0.675 (0.648 - 0.698)	0.97 (0.836 - 1.164)	0.036 (0.021 - 0.049)	1.165 (0.963 - 1.398)
CR7d_LR_c_1	24	0.006 (-0.005 - 0.016)	0.047 (0.036 - 0.064)	0.69 (0.659 - 0.727)	1.125 (0.855 - 1.414)	0.034 (0.019 - 0.043)	1.28 (1.063 - 1.572)
CR7d_LR_c_1	25	0.006 (-0.006 - 0.016)	0.05 (0.035 - 0.075)	0.696 (0.667 - 0.729)	1.075 (0.867 - 1.399)	0.033 (0.02 - 0.045)	1.267 (1.079 - 1.569)
CR7d_LR_c_1	26	0.006 (-0.008 - 0.014)	0.047 (0.035 - 0.072)	0.677 (0.642 - 0.71)	1.024 (0.818 - 1.306)	0.045 (0.026 - 0.062)	1.235 (1.023 - 1.585)
CR7d_LR_c_1	27	-0.002 (-0.014 - 0.006)	0.039 (0.03 - 0.045)	0.644 (0.614 - 0.671)	0.806 (0.62 - 1.014)	0.048 (0.034 - 0.064)	1.296 (1.027 - 1.593)
CR7d_LR_c_1	28	0.002 (-0.005 - 0.01)	0.044 (0.035 - 0.053)	0.665 (0.625 - 0.697)	0.945 (0.696 - 1.177)	0.049 (0.036 - 0.069)	1.121 (0.98 - 1.371)
CR7d_LR_c_1	29	0.007 (-0.003 - 0.012)	0.045 (0.036 - 0.057)	0.678 (0.644 - 0.715)	1.086 (0.824 - 1.341)	0.047 (0.034 - 0.07)	1.149 (0.983 - 1.392)
CR7d_LR_c_1	30	0.012 (0 - 0.02)	0.054 (0.04 - 0.065)	0.728 (0.686 - 0.767)	1.513 (1.078 - 1.957)	0.047 (0.033 - 0.068)	1.209 (0.996 - 1.466)
CR7d_LRCR_c_1	0	0.015 (0.012 - 0.018)	0.045 (0.04 - 0.049)	0.751 (0.742 - 0.762)	0.949 (0.869 - 1.008)	0.001 (0.001 - 0.003)	1.084 (1.019 - 1.158)

S-Table 8: Time-dependent metrics table *(continued)*

Model	LM	BSS	AUPRC	AUROC	Calibration slope	ECI	E:O ratio
CR7d_LRCR_c_1	1	0.021 (0.018 - 0.023)	0.056 (0.052 - 0.061)	0.764 (0.752 - 0.775)	1.019 (0.944 - 1.121)	0.002 (0.001 - 0.003)	1.07 (1.013 - 1.154)
CR7d_LRCR_c_1	2	0.023 (0.02 - 0.026)	0.062 (0.057 - 0.07)	0.764 (0.753 - 0.778)	1.067 (0.978 - 1.162)	0.002 (0.001 - 0.003)	1.012 (0.966 - 1.073)
CR7d_LRCR_c_1	3	0.025 (0.021 - 0.027)	0.067 (0.061 - 0.074)	0.761 (0.748 - 0.774)	1.048 (0.946 - 1.138)	0.003 (0.002 - 0.005)	0.995 (0.941 - 1.051)
CR7d_LRCR_c_1	4	0.029 (0.025 - 0.032)	0.078 (0.07 - 0.088)	0.77 (0.76 - 0.788)	1.081 (0.998 - 1.204)	0.005 (0.002 - 0.009)	0.958 (0.906 - 1.007)
CR7d_LRCR_c_1	5	0.026 (0.024 - 0.029)	0.073 (0.069 - 0.081)	0.775 (0.763 - 0.787)	1.097 (1.006 - 1.162)	0.004 (0.003 - 0.007)	0.948 (0.888 - 1.003)
CR7d_LRCR_c_1	6	0.024 (0.021 - 0.028)	0.071 (0.064 - 0.078)	0.757 (0.744 - 0.768)	1.039 (0.952 - 1.126)	0.005 (0.002 - 0.008)	0.963 (0.906 - 1.02)
CR7d_LRCR_c_1	7	0.024 (0.02 - 0.027)	0.075 (0.066 - 0.081)	0.754 (0.743 - 0.771)	1.048 (0.959 - 1.195)	0.005 (0.003 - 0.009)	0.988 (0.906 - 1.05)
CR7d_LRCR_c_1	8	0.025 (0.022 - 0.028)	0.077 (0.07 - 0.084)	0.756 (0.741 - 0.771)	1.077 (0.988 - 1.205)	0.006 (0.003 - 0.01)	0.967 (0.91 - 1.058)
CR7d_LRCR_c_1	9	0.03 (0.025 - 0.033)	0.092 (0.082 - 0.098)	0.757 (0.743 - 0.772)	1.117 (1.003 - 1.274)	0.011 (0.006 - 0.016)	0.92 (0.854 - 0.989)
CR7d_LRCR_c_1	10	0.025 (0.021 - 0.029)	0.082 (0.074 - 0.093)	0.739 (0.726 - 0.757)	1.047 (0.925 - 1.186)	0.011 (0.005 - 0.015)	0.947 (0.867 - 1.036)
CR7d_LRCR_c_1	11	0.026 (0.021 - 0.03)	0.086 (0.078 - 0.096)	0.743 (0.726 - 0.76)	1.058 (0.932 - 1.179)	0.011 (0.006 - 0.022)	0.928 (0.835 - 0.997)
CR7d_LRCR_c_1	12	0.025 (0.019 - 0.028)	0.084 (0.074 - 0.093)	0.736 (0.711 - 0.751)	1.016 (0.872 - 1.149)	0.01 (0.005 - 0.018)	0.959 (0.865 - 1.046)
CR7d_LRCR_c_1	13	0.023 (0.017 - 0.028)	0.086 (0.074 - 0.099)	0.728 (0.7 - 0.741)	1.012 (0.81 - 1.121)	0.012 (0.007 - 0.018)	0.941 (0.848 - 1.026)
CR7d_LRCR_c_1	14	0.023 (0.018 - 0.028)	0.085 (0.076 - 0.096)	0.731 (0.71 - 0.747)	1.038 (0.848 - 1.182)	0.011 (0.006 - 0.019)	0.994 (0.899 - 1.067)
CR7d_LRCR_c_1	15	0.026 (0.022 - 0.029)	0.094 (0.083 - 0.105)	0.732 (0.714 - 0.755)	1.064 (0.941 - 1.278)	0.014 (0.009 - 0.021)	0.964 (0.882 - 1.055)
CR7d_LRCR_c_1	16	0.025 (0.021 - 0.028)	0.089 (0.079 - 0.101)	0.739 (0.718 - 0.759)	1.104 (0.939 - 1.295)	0.014 (0.008 - 0.02)	1.054 (0.954 - 1.113)
CR7d_LRCR_c_1	17	0.022 (0.018 - 0.026)	0.107 (0.084 - 0.123)	0.717 (0.697 - 0.739)	0.995 (0.836 - 1.171)	0.02 (0.01 - 0.034)	0.907 (0.83 - 1.006)

S-Table 8: Time-dependent metrics table *(continued)*

Model	LM	BSS	AUPRC	AUROC	Calibration slope	ECI	E:O ratio
CR7d_LRCR_c_1	18	0.018 (0.014 - 0.023)	0.081 (0.07 - 0.095)	0.712 (0.68 - 0.737)	0.93 (0.763 - 1.222)	0.016 (0.009 - 0.026)	1.005 (0.912 - 1.12)
CR7d_LRCR_c_1	19	0.014 (0.009 - 0.02)	0.069 (0.059 - 0.082)	0.707 (0.678 - 0.729)	0.976 (0.823 - 1.204)	0.018 (0.012 - 0.028)	1.041 (0.943 - 1.225)
CR7d_LRCR_c_1	20	0.011 (0.005 - 0.016)	0.062 (0.053 - 0.076)	0.694 (0.663 - 0.722)	0.967 (0.809 - 1.178)	0.02 (0.012 - 0.03)	1.077 (0.926 - 1.276)
CR7d_LRCR_c_1	21	0.011 (0.001 - 0.016)	0.061 (0.046 - 0.074)	0.698 (0.662 - 0.718)	1.028 (0.799 - 1.239)	0.02 (0.012 - 0.031)	1.18 (0.975 - 1.373)
CR7d_LRCR_c_1	22	0.003 (-0.004 - 0.012)	0.046 (0.038 - 0.061)	0.685 (0.655 - 0.707)	1.061 (0.785 - 1.23)	0.028 (0.016 - 0.04)	1.246 (1.014 - 1.512)
CR7d_LRCR_c_1	23	0.008 (-0.001 - 0.014)	0.052 (0.038 - 0.076)	0.686 (0.65 - 0.71)	1.04 (0.879 - 1.233)	0.029 (0.019 - 0.049)	1.157 (0.959 - 1.406)
CR7d_LRCR_c_1	24	0.008 (-0.005 - 0.017)	0.048 (0.036 - 0.065)	0.695 (0.666 - 0.733)	1.183 (0.898 - 1.485)	0.031 (0.017 - 0.043)	1.269 (1.077 - 1.568)
CR7d_LRCR_c_1	25	0.007 (-0.005 - 0.017)	0.05 (0.037 - 0.079)	0.698 (0.665 - 0.735)	1.133 (0.861 - 1.466)	0.03 (0.021 - 0.045)	1.252 (1.071 - 1.575)
CR7d_LRCR_c_1	26	0.006 (-0.008 - 0.015)	0.046 (0.034 - 0.073)	0.679 (0.641 - 0.71)	1.087 (0.818 - 1.31)	0.039 (0.027 - 0.059)	1.269 (1.027 - 1.598)
CR7d_LRCR_c_1	27	0.001 (-0.013 - 0.007)	0.039 (0.028 - 0.047)	0.646 (0.612 - 0.673)	0.805 (0.614 - 1.046)	0.046 (0.028 - 0.064)	1.294 (1.012 - 1.585)
CR7d_LRCR_c_1	28	0.004 (-0.005 - 0.01)	0.045 (0.035 - 0.052)	0.667 (0.629 - 0.697)	0.938 (0.703 - 1.169)	0.049 (0.035 - 0.068)	1.134 (0.989 - 1.34)
CR7d_LRCR_c_1	29	0.008 (-0.003 - 0.013)	0.045 (0.037 - 0.057)	0.689 (0.646 - 0.719)	1.121 (0.85 - 1.385)	0.048 (0.033 - 0.072)	1.155 (0.964 - 1.359)
CR7d_LRCR_c_1	30	0.014 (0.003 - 0.021)	0.054 (0.041 - 0.068)	0.736 (0.691 - 0.772)	1.507 (1.122 - 1.999)	0.045 (0.028 - 0.065)	1.212 (0.991 - 1.419)
CR7d_LR_c_all	0	0.015 (0.012 - 0.018)	0.045 (0.04 - 0.05)	0.744 (0.734 - 0.757)	0.981 (0.901 - 1.05)	0.001 (0.001 - 0.002)	1.086 (1.018 - 1.149)
CR7d_LR_c_all	1	0.021 (0.018 - 0.024)	0.056 (0.052 - 0.061)	0.763 (0.75 - 0.772)	1.048 (0.942 - 1.114)	0.002 (0.001 - 0.003)	1.085 (1.013 - 1.149)
CR7d_LR_c_all	2	0.023 (0.02 - 0.025)	0.06 (0.056 - 0.067)	0.76 (0.747 - 0.771)	1.089 (0.995 - 1.176)	0.002 (0.001 - 0.004)	1.051 (0.992 - 1.106)
CR7d_LR_c_all	3	0.025 (0.021 - 0.028)	0.067 (0.062 - 0.073)	0.758 (0.742 - 0.773)	1.065 (0.974 - 1.194)	0.003 (0.002 - 0.005)	1.034 (0.993 - 1.09)

S-Table 8: Time-dependent metrics table *(continued)*

Model	LM	BSS	AUPRC	AUROC	Calibration slope	ECI	E:O ratio
CR7d_LR_c_all	4	0.028 (0.024 - 0.032)	0.076 (0.07 - 0.084)	0.767 (0.751 - 0.78)	1.102 (1.008 - 1.206)	0.003 (0.002 - 0.006)	0.997 (0.95 - 1.046)
CR7d_LR_c_all	5	0.026 (0.022 - 0.029)	0.072 (0.067 - 0.079)	0.77 (0.759 - 0.783)	1.07 (1.008 - 1.186)	0.005 (0.002 - 0.006)	0.97 (0.909 - 1.029)
CR7d_LR_c_all	6	0.022 (0.018 - 0.026)	0.066 (0.061 - 0.074)	0.749 (0.735 - 0.763)	1.01 (0.918 - 1.085)	0.004 (0.003 - 0.009)	0.975 (0.91 - 1.026)
CR7d_LR_c_all	7	0.021 (0.018 - 0.025)	0.067 (0.061 - 0.074)	0.75 (0.735 - 0.766)	1.016 (0.923 - 1.128)	0.006 (0.004 - 0.011)	0.989 (0.902 - 1.052)
CR7d_LR_c_all	8	0.022 (0.019 - 0.026)	0.071 (0.065 - 0.077)	0.746 (0.732 - 0.76)	1.045 (0.936 - 1.157)	0.008 (0.004 - 0.011)	0.966 (0.908 - 1.04)
CR7d_LR_c_all	9	0.027 (0.024 - 0.031)	0.087 (0.078 - 0.094)	0.754 (0.738 - 0.767)	1.137 (0.994 - 1.241)	0.01 (0.005 - 0.015)	0.919 (0.86 - 0.991)
CR7d_LR_c_all	10	0.024 (0.02 - 0.028)	0.083 (0.073 - 0.091)	0.741 (0.728 - 0.757)	1.078 (0.971 - 1.218)	0.01 (0.005 - 0.017)	0.94 (0.867 - 1.029)
CR7d_LR_c_all	11	0.025 (0.02 - 0.029)	0.085 (0.076 - 0.091)	0.745 (0.727 - 0.763)	1.108 (0.986 - 1.267)	0.011 (0.008 - 0.02)	0.925 (0.836 - 0.986)
CR7d_LR_c_all	12	0.024 (0.019 - 0.029)	0.087 (0.077 - 0.098)	0.735 (0.711 - 0.749)	1.077 (0.907 - 1.213)	0.013 (0.007 - 0.021)	0.952 (0.869 - 1.028)
CR7d_LR_c_all	13	0.023 (0.017 - 0.027)	0.088 (0.076 - 0.099)	0.722 (0.699 - 0.74)	1.074 (0.887 - 1.216)	0.015 (0.008 - 0.022)	0.935 (0.848 - 1.014)
CR7d_LR_c_all	14	0.025 (0.021 - 0.029)	0.09 (0.081 - 0.108)	0.73 (0.712 - 0.746)	1.136 (0.987 - 1.332)	0.014 (0.008 - 0.029)	0.98 (0.895 - 1.056)
CR7d_LR_c_all	15	0.026 (0.022 - 0.029)	0.102 (0.088 - 0.12)	0.73 (0.711 - 0.746)	1.177 (0.992 - 1.317)	0.021 (0.013 - 0.034)	0.952 (0.868 - 1.041)
CR7d_LR_c_all	16	0.026 (0.022 - 0.029)	0.097 (0.083 - 0.11)	0.737 (0.717 - 0.758)	1.219 (1.014 - 1.399)	0.017 (0.011 - 0.032)	1.037 (0.935 - 1.091)
CR7d_LR_c_all	17	0.022 (0.016 - 0.025)	0.092 (0.079 - 0.112)	0.715 (0.691 - 0.733)	1.058 (0.873 - 1.254)	0.021 (0.012 - 0.033)	0.884 (0.817 - 0.97)
CR7d_LR_c_all	18	0.019 (0.013 - 0.023)	0.082 (0.071 - 0.096)	0.705 (0.677 - 0.735)	1.021 (0.817 - 1.265)	0.017 (0.011 - 0.03)	0.977 (0.9 - 1.089)
CR7d_LR_c_all	19	0.013 (0.009 - 0.019)	0.066 (0.058 - 0.077)	0.705 (0.674 - 0.725)	1.021 (0.847 - 1.179)	0.018 (0.012 - 0.024)	1.04 (0.917 - 1.19)
CR7d_LR_c_all	20	0.012 (0.006 - 0.017)	0.062 (0.054 - 0.076)	0.7 (0.671 - 0.727)	0.996 (0.825 - 1.244)	0.019 (0.013 - 0.029)	1.065 (0.895 - 1.23)

S-Table 8: Time-dependent metrics table *(continued)*

Model	LM	BSS	AUPRC	AUROC	Calibration slope	ECI	E:O ratio
CR7d_LR_c_all	21	0.008 (0.002 - 0.014)	0.056 (0.046 - 0.074)	0.684 (0.651 - 0.715)	0.974 (0.751 - 1.154)	0.022 (0.014 - 0.03)	1.153 (0.949 - 1.311)
CR7d_LR_c_all	22	0.004 (-0.003 - 0.012)	0.046 (0.039 - 0.063)	0.682 (0.652 - 0.713)	0.98 (0.778 - 1.146)	0.027 (0.016 - 0.041)	1.186 (0.984 - 1.423)
CR7d_LR_c_all	23	0.006 (-0.002 - 0.011)	0.046 (0.036 - 0.063)	0.675 (0.646 - 0.701)	0.943 (0.752 - 1.153)	0.032 (0.022 - 0.049)	1.118 (0.931 - 1.34)
CR7d_LR_c_all	24	0.007 (-0.005 - 0.015)	0.047 (0.033 - 0.066)	0.7 (0.668 - 0.73)	1.13 (0.866 - 1.403)	0.034 (0.022 - 0.046)	1.212 (1.008 - 1.479)
CR7d_LR_c_all	25	0.006 (-0.007 - 0.013)	0.047 (0.035 - 0.073)	0.686 (0.655 - 0.715)	1.005 (0.779 - 1.245)	0.034 (0.021 - 0.05)	1.187 (1.022 - 1.507)
CR7d_LR_c_all	26	0.007 (-0.003 - 0.013)	0.048 (0.037 - 0.076)	0.677 (0.639 - 0.707)	0.966 (0.76 - 1.183)	0.039 (0.026 - 0.058)	1.165 (0.968 - 1.491)
CR7d_LR_c_all	27	0 (-0.007 - 0.007)	0.039 (0.031 - 0.05)	0.645 (0.611 - 0.683)	0.784 (0.631 - 1.056)	0.039 (0.028 - 0.054)	1.154 (0.944 - 1.426)
CR7d_LR_c_all	28	0.004 (-0.003 - 0.009)	0.044 (0.035 - 0.054)	0.654 (0.634 - 0.688)	0.849 (0.657 - 1.127)	0.042 (0.031 - 0.058)	1.029 (0.868 - 1.202)
CR7d_LR_c_all	29	0.008 (0.001 - 0.016)	0.052 (0.04 - 0.067)	0.697 (0.658 - 0.725)	1.075 (0.839 - 1.387)	0.038 (0.024 - 0.057)	1.037 (0.874 - 1.26)
CR7d_LR_c_all	30	0.011 (0.003 - 0.019)	0.055 (0.04 - 0.072)	0.717 (0.678 - 0.76)	1.236 (0.973 - 1.629)	0.039 (0.025 - 0.056)	1.06 (0.875 - 1.261)
CR7d_LRCR_c_all	0	0.015 (0.013 - 0.018)	0.046 (0.041 - 0.05)	0.745 (0.735 - 0.756)	0.96 (0.897 - 1.023)	0.002 (0.001 - 0.003)	1.086 (1.018 - 1.156)
CR7d_LRCR_c_all	1	0.02 (0.019 - 0.024)	0.056 (0.052 - 0.062)	0.764 (0.75 - 0.772)	1.031 (0.946 - 1.106)	0.002 (0.001 - 0.003)	1.08 (1.011 - 1.145)
CR7d_LRCR_c_all	2	0.023 (0.02 - 0.026)	0.063 (0.057 - 0.068)	0.762 (0.749 - 0.773)	1.079 (0.973 - 1.142)	0.002 (0.001 - 0.004)	1.046 (0.99 - 1.102)
CR7d_LRCR_c_all	3	0.025 (0.022 - 0.029)	0.069 (0.063 - 0.076)	0.759 (0.747 - 0.774)	1.068 (0.972 - 1.163)	0.003 (0.002 - 0.005)	1.031 (0.985 - 1.089)
CR7d_LRCR_c_all	4	0.029 (0.025 - 0.032)	0.077 (0.069 - 0.084)	0.768 (0.753 - 0.779)	1.079 (0.986 - 1.183)	0.003 (0.002 - 0.006)	0.989 (0.941 - 1.043)
CR7d_LRCR_c_all	5	0.026 (0.023 - 0.029)	0.072 (0.068 - 0.079)	0.77 (0.76 - 0.784)	1.079 (0.99 - 1.177)	0.004 (0.003 - 0.006)	0.964 (0.903 - 1.024)
CR7d_LRCR_c_all	6	0.022 (0.018 - 0.026)	0.067 (0.061 - 0.075)	0.748 (0.735 - 0.763)	0.983 (0.909 - 1.084)	0.004 (0.003 - 0.008)	0.97 (0.904 - 1.019)

S-Table 8: Time-dependent metrics table *(continued)*

Model	LM	BSS	AUPRC	AUROC	Calibration slope	ECI	E:O ratio
CR7d_LRCR_c_all	7	0.022 (0.017 - 0.025)	0.068 (0.062 - 0.074)	0.75 (0.738 - 0.764)	1.004 (0.908 - 1.109)	0.006 (0.004 - 0.01)	0.988 (0.895 - 1.047)
CR7d_LRCR_c_all	8	0.022 (0.019 - 0.026)	0.072 (0.066 - 0.08)	0.747 (0.733 - 0.761)	1.027 (0.924 - 1.117)	0.007 (0.004 - 0.01)	0.959 (0.906 - 1.04)
CR7d_LRCR_c_all	9	0.028 (0.023 - 0.03)	0.086 (0.079 - 0.094)	0.753 (0.74 - 0.764)	1.112 (1.008 - 1.194)	0.01 (0.005 - 0.015)	0.913 (0.858 - 0.983)
CR7d_LRCR_c_all	10	0.024 (0.02 - 0.028)	0.082 (0.073 - 0.092)	0.741 (0.727 - 0.756)	1.07 (0.965 - 1.184)	0.011 (0.006 - 0.017)	0.943 (0.865 - 1.028)
CR7d_LRCR_c_all	11	0.025 (0.02 - 0.029)	0.085 (0.077 - 0.093)	0.745 (0.728 - 0.762)	1.091 (0.961 - 1.255)	0.012 (0.008 - 0.02)	0.926 (0.835 - 0.985)
CR7d_LRCR_c_all	12	0.024 (0.019 - 0.029)	0.087 (0.078 - 0.096)	0.738 (0.714 - 0.752)	1.06 (0.916 - 1.193)	0.013 (0.007 - 0.021)	0.961 (0.867 - 1.035)
CR7d_LRCR_c_all	13	0.023 (0.017 - 0.027)	0.087 (0.075 - 0.098)	0.724 (0.698 - 0.744)	1.058 (0.857 - 1.2)	0.013 (0.007 - 0.022)	0.928 (0.84 - 1.013)
CR7d_LRCR_c_all	14	0.026 (0.02 - 0.029)	0.092 (0.082 - 0.108)	0.731 (0.712 - 0.745)	1.145 (0.97 - 1.282)	0.015 (0.008 - 0.027)	0.978 (0.895 - 1.055)
CR7d_LRCR_c_all	15	0.027 (0.022 - 0.03)	0.104 (0.091 - 0.124)	0.732 (0.716 - 0.746)	1.176 (1.004 - 1.272)	0.02 (0.014 - 0.036)	0.963 (0.876 - 1.051)
CR7d_LRCR_c_all	16	0.026 (0.022 - 0.03)	0.101 (0.089 - 0.117)	0.74 (0.717 - 0.757)	1.192 (1.025 - 1.351)	0.019 (0.012 - 0.033)	1.038 (0.942 - 1.099)
CR7d_LRCR_c_all	17	0.022 (0.017 - 0.026)	0.094 (0.079 - 0.114)	0.717 (0.697 - 0.734)	1.05 (0.888 - 1.226)	0.02 (0.012 - 0.029)	0.888 (0.819 - 0.968)
CR7d_LRCR_c_all	18	0.019 (0.013 - 0.024)	0.085 (0.074 - 0.101)	0.705 (0.679 - 0.738)	0.996 (0.84 - 1.242)	0.018 (0.011 - 0.029)	0.983 (0.896 - 1.089)
CR7d_LRCR_c_all	19	0.014 (0.009 - 0.02)	0.067 (0.058 - 0.08)	0.707 (0.676 - 0.726)	1.006 (0.824 - 1.163)	0.019 (0.012 - 0.025)	1.037 (0.92 - 1.191)
CR7d_LRCR_c_all	20	0.013 (0.007 - 0.017)	0.066 (0.054 - 0.078)	0.701 (0.672 - 0.728)	0.975 (0.844 - 1.238)	0.019 (0.012 - 0.031)	1.051 (0.899 - 1.234)
CR7d_LRCR_c_all	21	0.009 (0.003 - 0.016)	0.058 (0.048 - 0.072)	0.686 (0.656 - 0.719)	0.938 (0.775 - 1.202)	0.021 (0.012 - 0.033)	1.15 (0.934 - 1.317)
CR7d_LRCR_c_all	22	0.006 (-0.001 - 0.013)	0.048 (0.04 - 0.069)	0.687 (0.658 - 0.716)	0.989 (0.803 - 1.161)	0.028 (0.019 - 0.039)	1.196 (0.974 - 1.429)
CR7d_LRCR_c_all	23	0.006 (-0.001 - 0.011)	0.048 (0.037 - 0.067)	0.674 (0.648 - 0.706)	0.933 (0.775 - 1.165)	0.033 (0.026 - 0.047)	1.106 (0.929 - 1.332)

S-Table 8: Time-dependent metrics table *(continued)*

Model	LM	BSS	AUPRC	AUROC	Calibration slope	ECI	E:O ratio
CR7d_LRCR_c_all	24	0.008 (-0.004 - 0.015)	0.049 (0.036 - 0.069)	0.697 (0.669 - 0.729)	1.13 (0.893 - 1.413)	0.032 (0.024 - 0.045)	1.218 (0.991 - 1.48)
CR7d_LRCR_c_all	25	0.007 (-0.006 - 0.014)	0.048 (0.036 - 0.074)	0.688 (0.657 - 0.721)	0.987 (0.818 - 1.288)	0.033 (0.022 - 0.049)	1.178 (1.017 - 1.485)
CR7d_LRCR_c_all	26	0.008 (-0.003 - 0.013)	0.052 (0.038 - 0.085)	0.681 (0.643 - 0.714)	0.996 (0.758 - 1.208)	0.041 (0.026 - 0.059)	1.16 (0.954 - 1.473)
CR7d_LRCR_c_all	27	0 (-0.007 - 0.007)	0.04 (0.031 - 0.05)	0.646 (0.61 - 0.686)	0.837 (0.608 - 1.05)	0.039 (0.023 - 0.058)	1.142 (0.928 - 1.421)
CR7d_LRCR_c_all	28	0.004 (-0.004 - 0.009)	0.044 (0.034 - 0.056)	0.658 (0.632 - 0.687)	0.866 (0.682 - 1.103)	0.043 (0.032 - 0.058)	1.001 (0.861 - 1.2)
CR7d_LRCR_c_all	29	0.009 (0.001 - 0.015)	0.051 (0.04 - 0.065)	0.698 (0.661 - 0.727)	1.037 (0.814 - 1.403)	0.038 (0.022 - 0.055)	1.032 (0.848 - 1.208)
CR7d_LRCR_c_all	30	0.011 (0.003 - 0.02)	0.053 (0.04 - 0.071)	0.714 (0.675 - 0.757)	1.216 (0.93 - 1.59)	0.04 (0.029 - 0.053)	1.041 (0.858 - 1.237)

8.3 Pooled model evaluation

In contrast to the time-dependent evaluation, the “pooled” evaluation considers all landmarks in the model pooled together (each landmark represents an independent observation). The pooled evaluation metric are presented in S-Figure 19. The findings are similar to the time-dependent evaluation, with the survival model with competing events censoring at their event time showing poorer performance especially in terms of calibration.

8.4 ROC curves

ROC curves are calculated using the “pooled” predictions (each landmark represents an independent observation) and presented in S-Figure 20 for each test set and each model.

8.5 Precision-recall curves

Precision-recall curves are calculated using the “pooled” predictions (each landmark represents an independent observation) and presented in S-Figure 21 for each test set and each model.

8.6 Calibration curves - deciles

Calibration curves are calculated as for the static model, using the “pooled” predictions (each landmark represents an independent observation). The survival model with competing risks censored at the time of event shows overestimated predictions. The deciles calibration curves for dynamic models are presented in S-Figure 22.

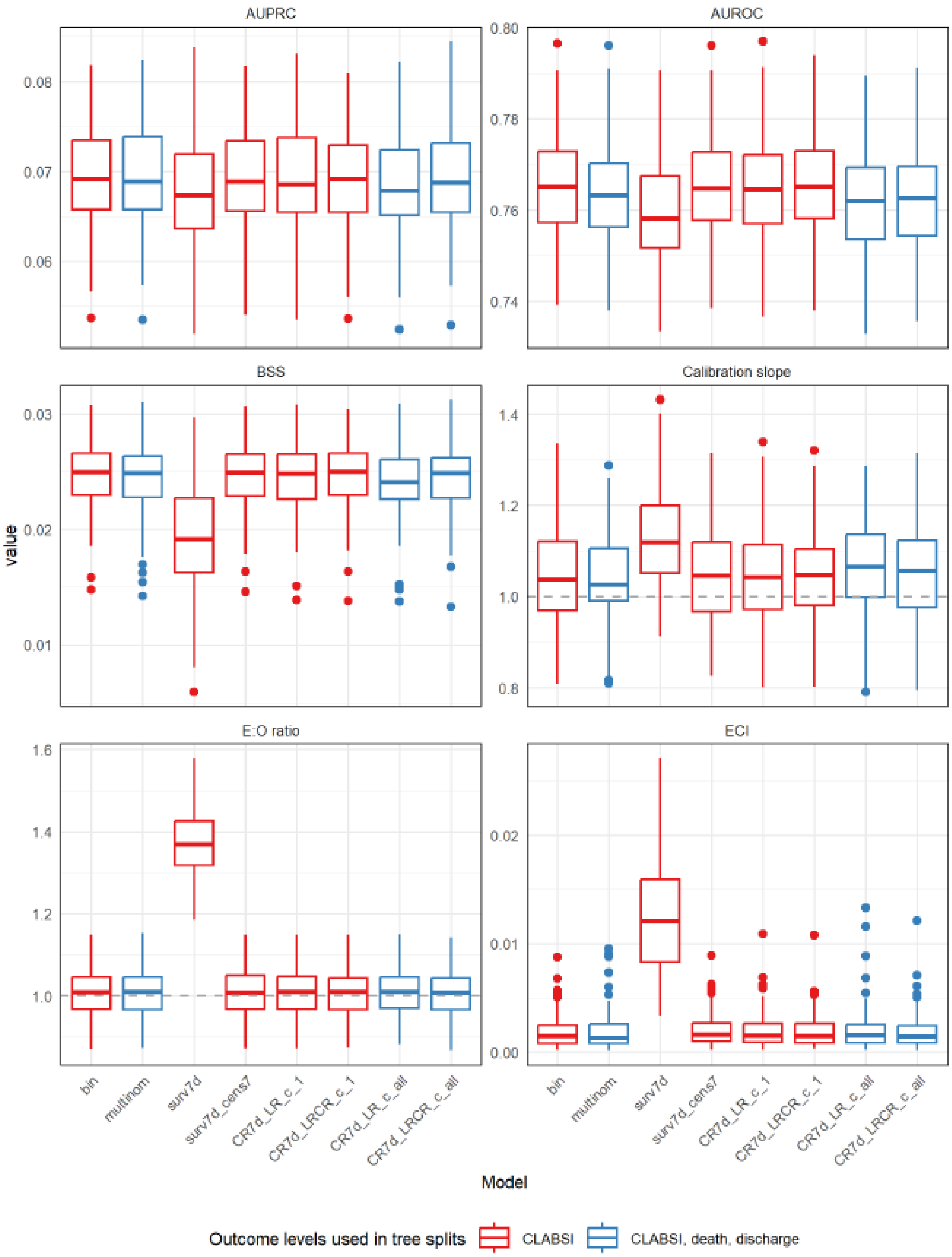

S-Figure 19: Prediction performance for dynamic models - pooled metrics

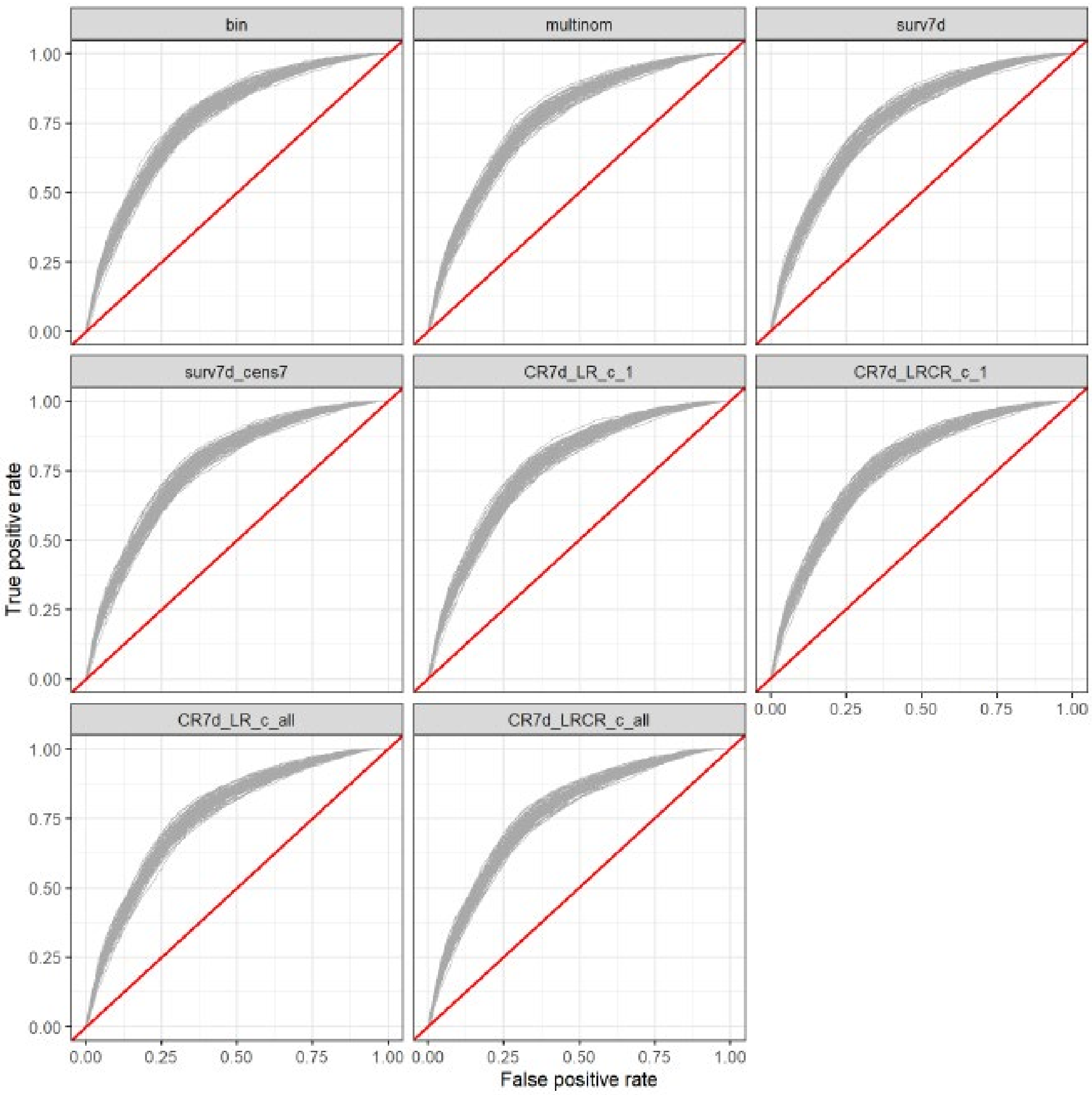

S-Figure 20: ROC curves for dynamic models

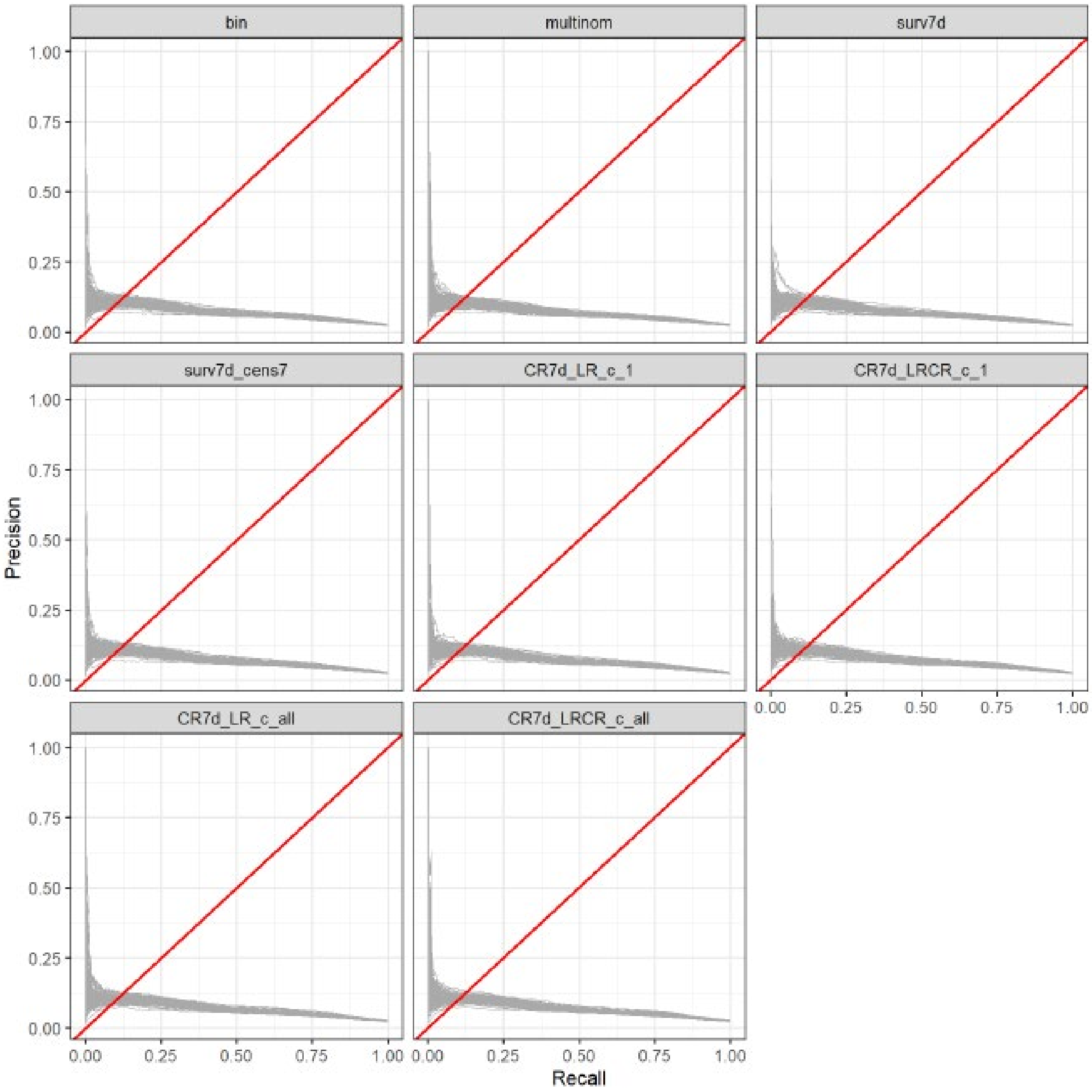

S-Figure 21: Precision-recall curves for dynamic models

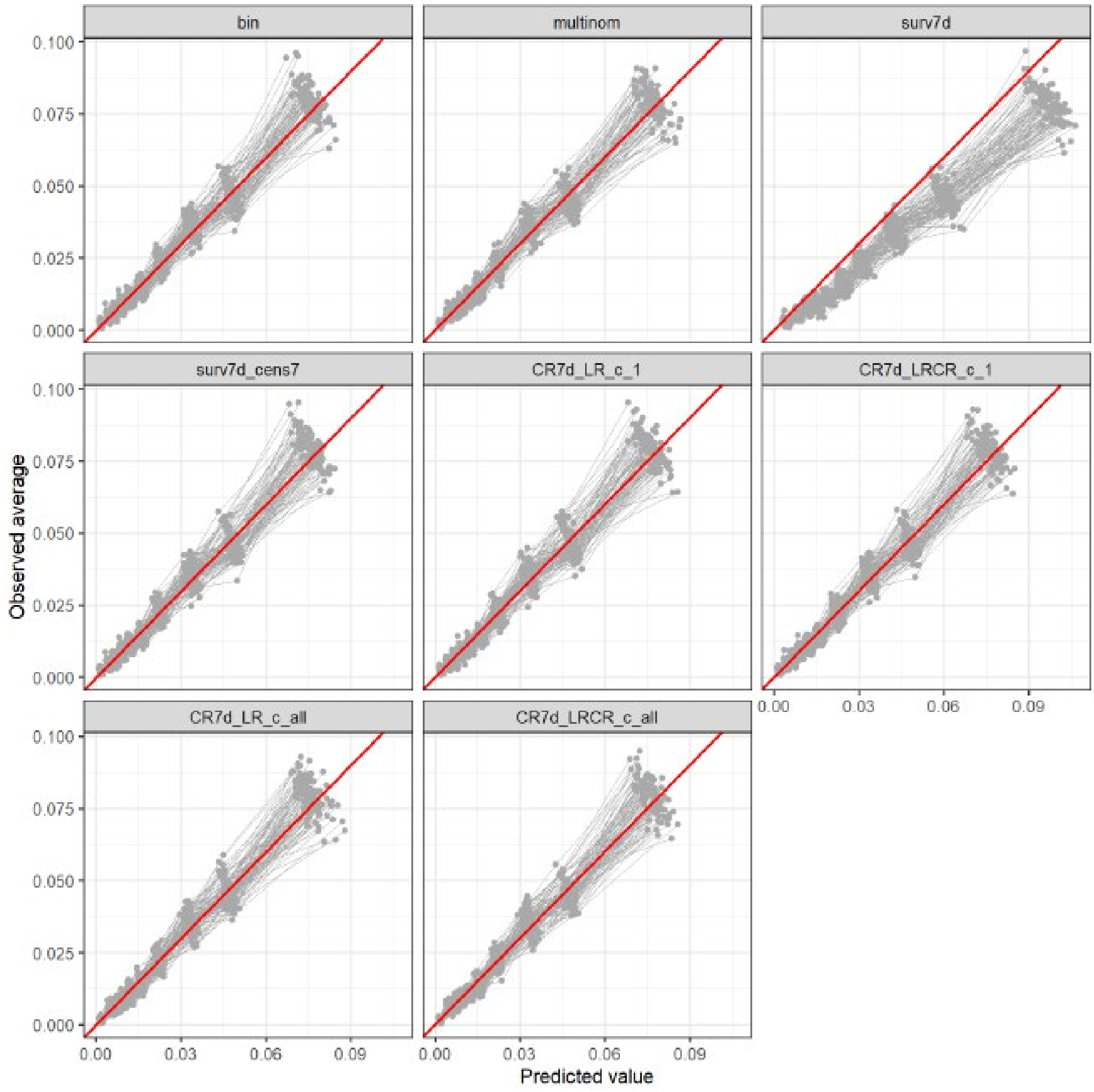

8.7 Calibration curves - splines

Calibration curves are calculated as for the static model, using the “pooled” predictions (each landmark represents an independent observation). The survival model with competing risks censored at the time of event shows overestimated predictions. The calibration curves based on cubic splines are presented in S-Figure 23.

8.8 Decision curves

Decision curves are calculated as for the static model, using the “pooled” predictions (each landmark represents an independent observation). The decision curves for dynamic models are presented in S-Figure 24.

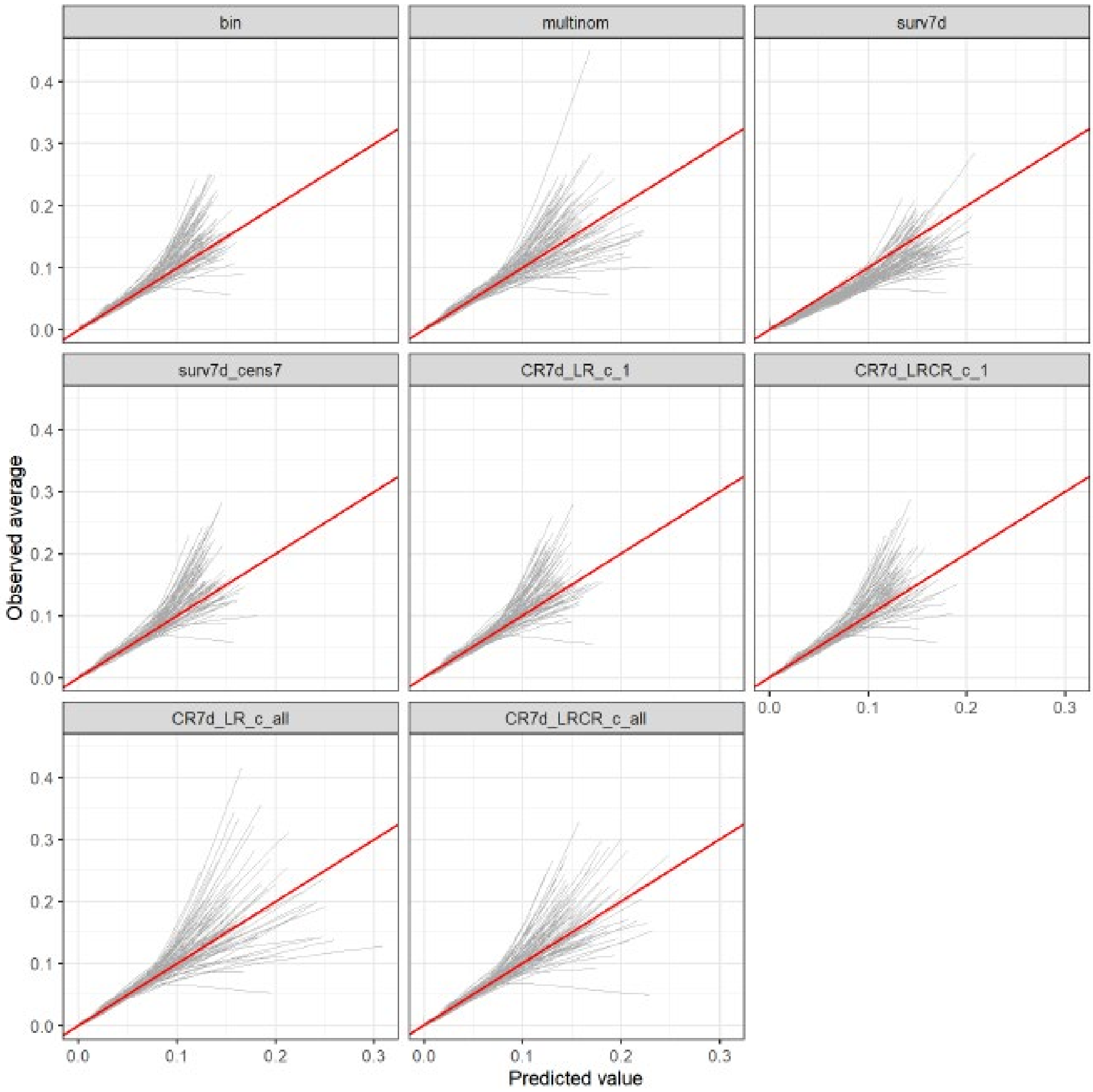

S-Figure 23: Calibration curves (splines) for dynamic models

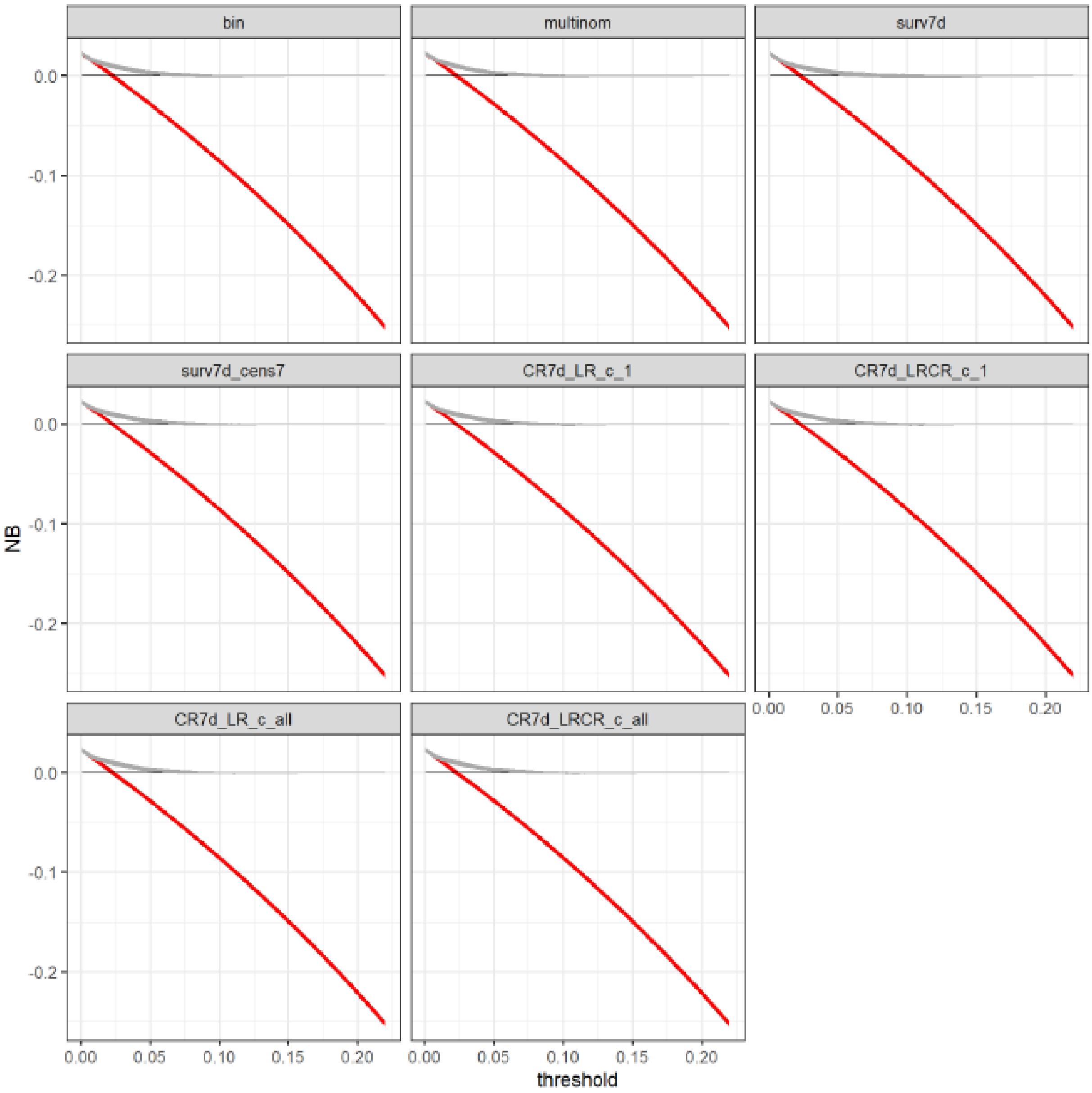

S-Figure 24: Decision curves for dynamic models

8.9 Predictions density curves

Prediction density curves for dynamic models using the "pooled" predictions (each landmark represents an independent observation) are presented in S-Figure 25. As opposed to the static models, there is no notable difference between models.

8.10 Tuned hyperparameters

The tuned hyperparameters for the dynamic models (mtry, nodesize and sample.fraction) are shown in S-Figures 26, 27 and 28. As for static models, models using all events in the outcome definition (multinomial and CR models weighting all causes), have tuned nodesizes lower than the other models. The sample fraction hyperparameter does not show any specific pattern.

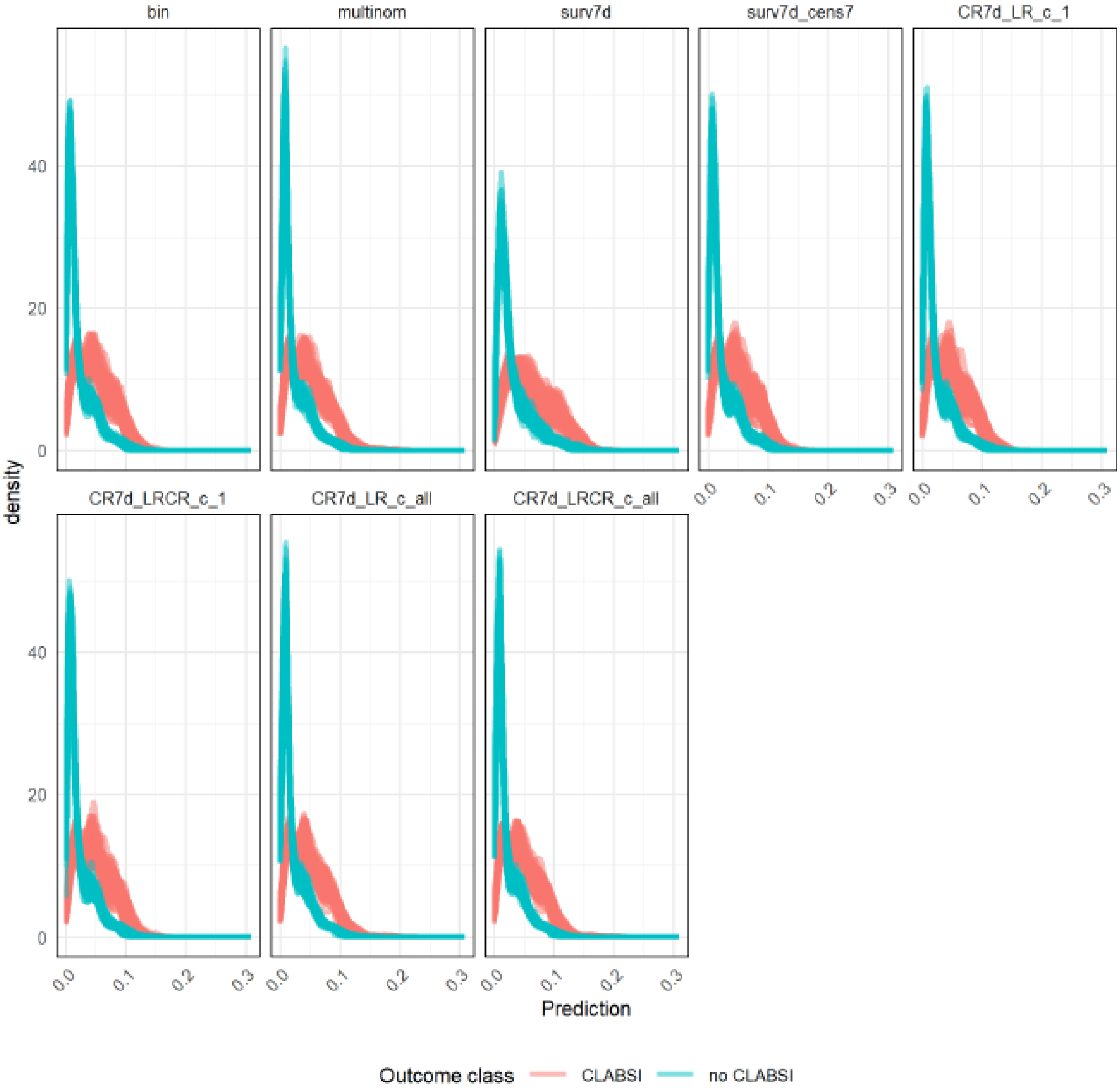

S-Figure 25: Density curves of predicted risks by outcome for dynamic models

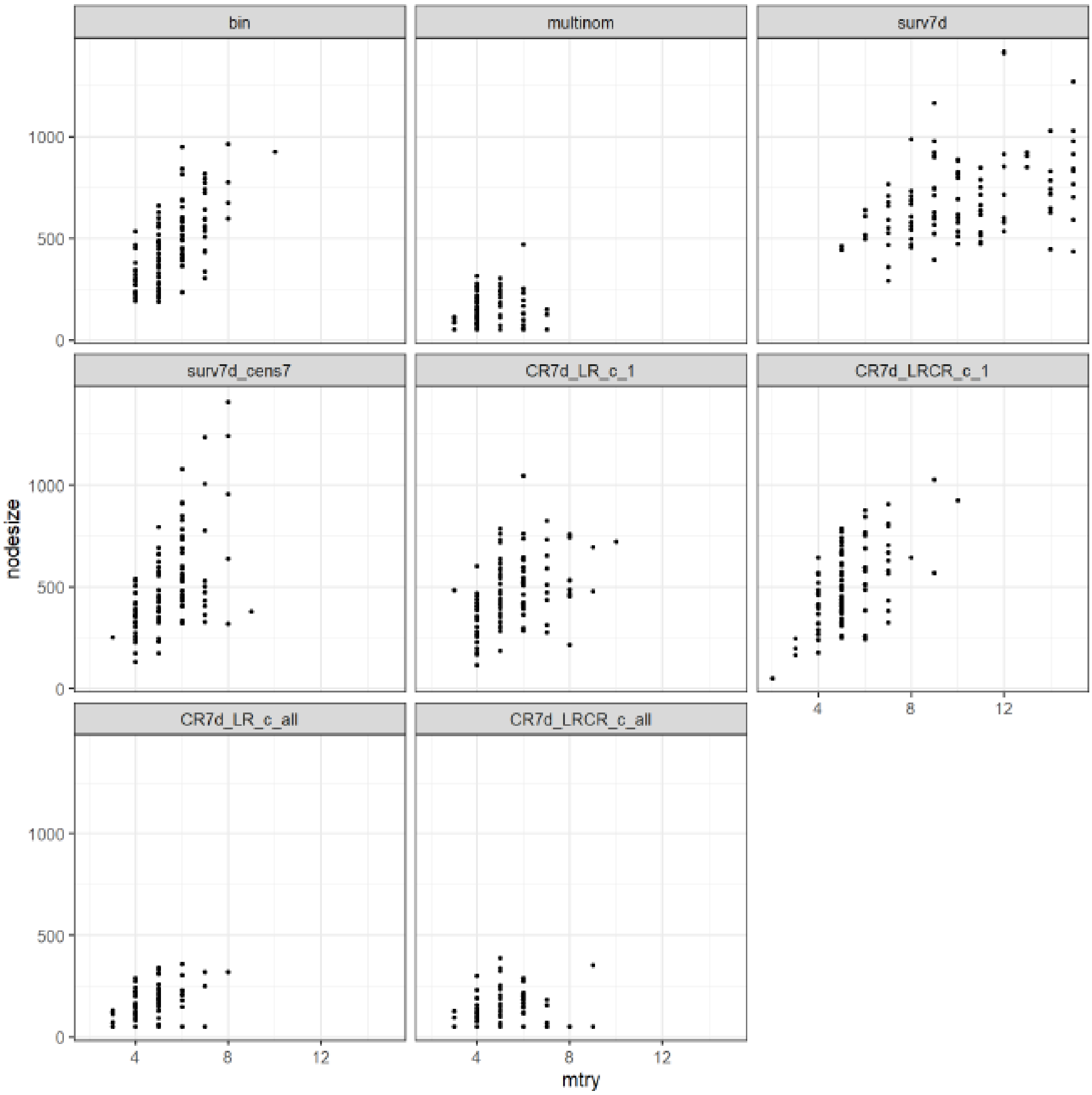

S-Figure 26: Tune hyperparameters (nodesize in function of mtry)

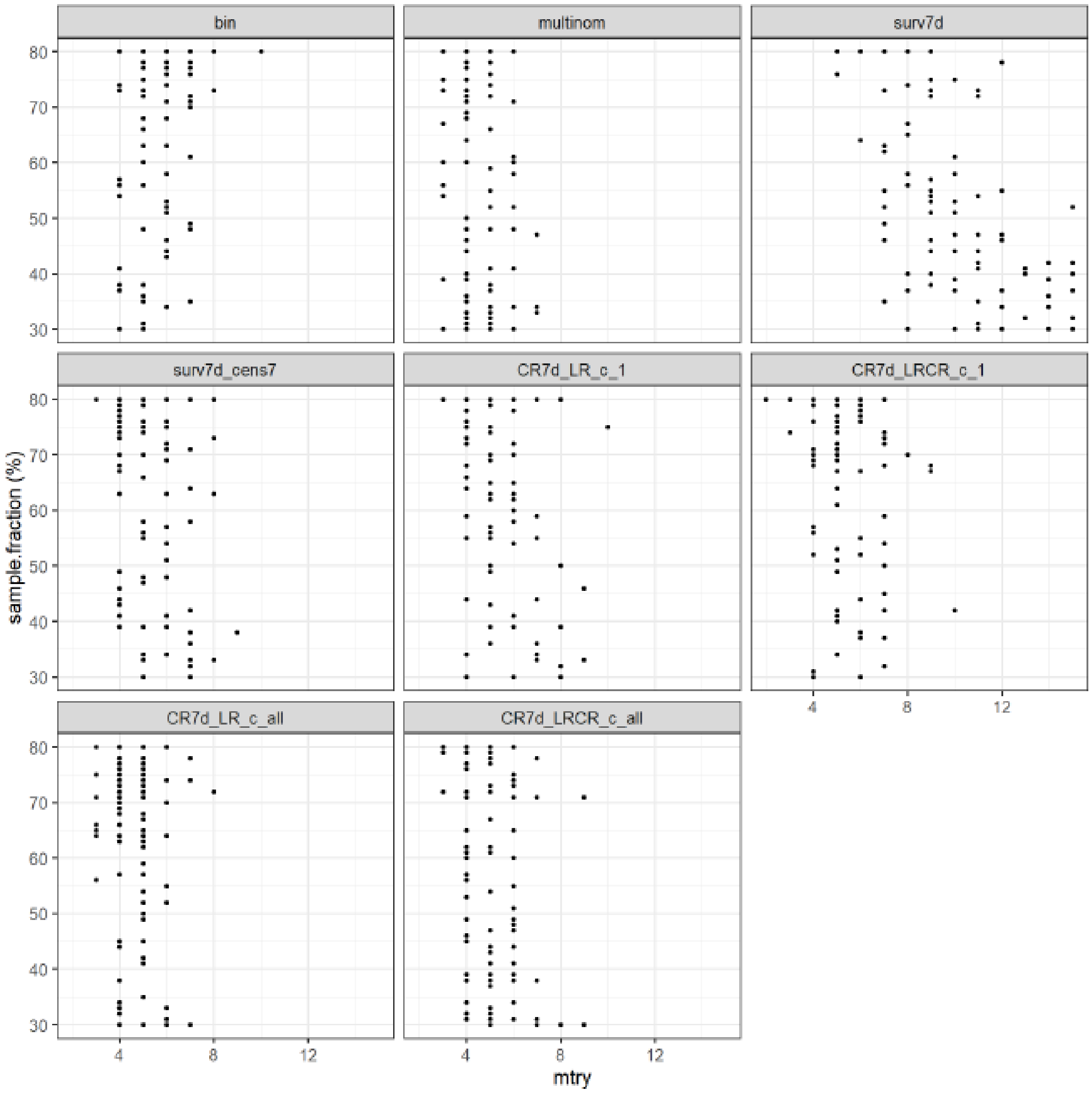

S-Figure 27: Tune hyperparameters (sample.fraction in function of mtry)

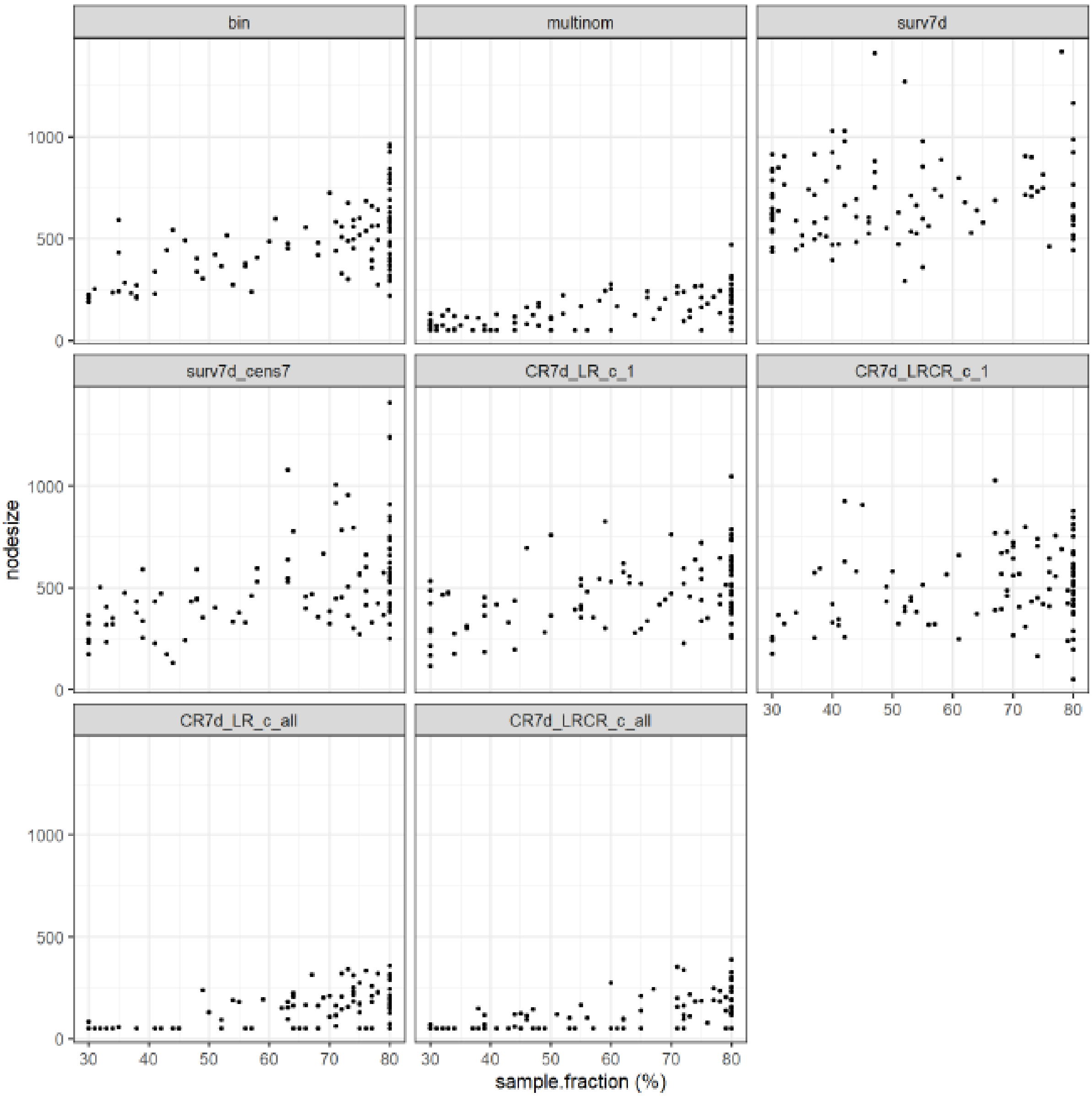

S-Figure 28: Tune hyperparameters (nodesize in function of sample.fraction)

8.11 Variable importance

The minimal depth of the maximal subtree is used as a variable importance metric (Ishwaran et al. 2021), which is the depth in a tree on which the first split is made on a variable *v*, averaged over all trees in the forest. The lowest possible value is 0 (root node split). The minimal depth of the maximal subtree for dynamic models is presented in S-Figure 29. A guiding line has been added to the plot on value 2, an aleatory choice to guide the focus on most important variables.

Models using multiple levels of the outcome put more weight (splits closer to the root) on variables: ICU, antibacterials, antineoplastic agents (chemotherapy) and CRP, “other infection than BSI” and tunneled catheters. The differences in the minimal depth for variables TPN and port-catheter are less notable than for static models.

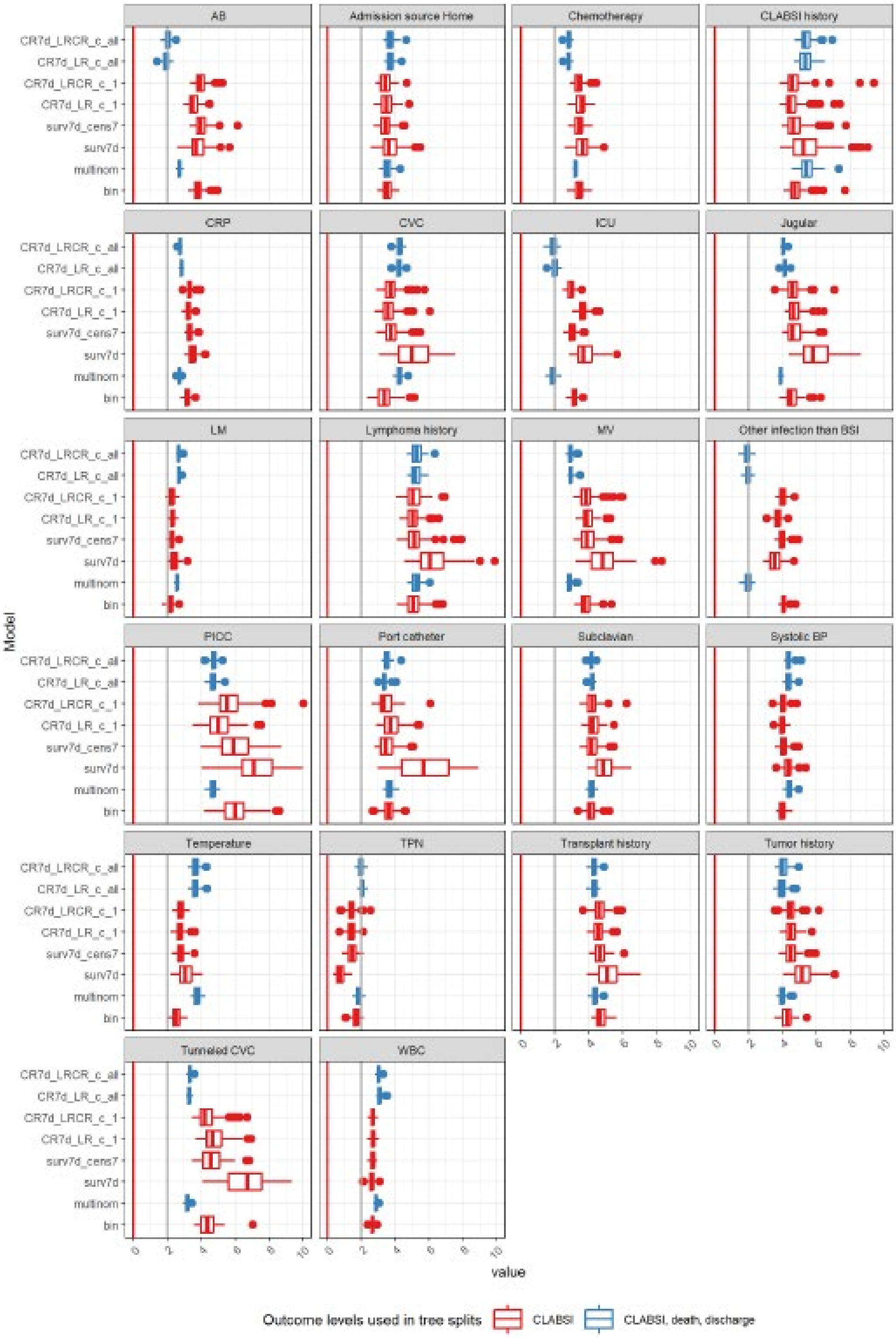

S-Figure 29: Variable importance for dynamic models

Supplementary material 9 - Timings table

The runtimes for static and dynamic models are presented in S-Table 9.

S-Table 9: Runtimes for all models (in seconds)

Model	Static / Dynamic	Tuning	Build final model	Predict
bin	Static	209.404 (197.433 - 218.468)	11.641 (10.403 - 12.321)	0.346 (0.327 - 0.37)
multinom	Static	226.332 (208.266 - 236.412)	7.073 (6.541 - 7.567)	0.377 (0.345 - 0.4)
surv7d	Static	370.972 (355.951 - 385.155)	9.201 (8.297 - 9.667)	0.296 (0.288 - 0.306)
surv7d_cens7	Static	262.443 (249.593 - 274.153)	7.129 (6.851 - 7.452)	0.289 (0.278 - 0.307)
surv30d	Static	459.341 (446.094 - 476.892)	10.512 (9.328 - 11.825)	0.319 (0.309 - 0.328)
surv30d_cens7	Static	378.045 (363.976 - 393.637)	7.893 (7.504 - 8.279)	0.323 (0.308 - 0.34)
CR7d_LR_c_1	Static	447.95 (431.07 - 472.655)	8.851 (8.475 - 9.445)	0.488 (0.465 - 0.509)
CR7d_LRCR_c_1	Static	468.8 (445.506 - 486.273)	9.578 (8.996 - 11.015)	0.466 (0.445 - 0.491)
CR7d_LR_c_all	Static	401.085 (384.14 - 426.019)	9.288 (8.962 - 10.001)	0.481 (0.474 - 0.49)
CR7d_LRCR_c_all	Static	426.47 (408.683 - 460.104)	9.728 (9.195 - 10.307)	0.514 (0.497 - 0.527)
CR30d_LR_c_1	Static	567.148 (545.541 - 590.394)	10.082 (9.295 - 11.126)	0.518 (0.493 - 0.532)
CR30d_LRCR_c_1	Static	543.608 (526.061 - 578.87)	10.339 (9.373 - 11.483)	0.486 (0.467 - 0.513)
CR30d_LR_c_all	Static	539.688 (513.044 - 573.47)	11.179 (10.126 - 13.222)	0.526 (0.512 - 0.546)
CR30d_LRCR_c_all	Static	552.918 (525.582 - 582.852)	11.528 (10.614 - 13.562)	0.528 (0.513 - 0.548)
bin	Dynamic	760.216 (736.662 - 786.84)	209.655 (205.827 - 213.852)	1.884 (1.778 - 2.019)
multinom	Dynamic	843.682 (815.876 - 866.967)	214.542 (209.687 - 219.267)	2.326 (2.178 - 2.511)
surv7d	Dynamic	935.971 (896.896 - 970.382)	207.88 (203.285 - 213.398)	2.25 (2.025 - 2.441)
surv7d_cens7	Dynamic	776.992 (747.751 - 804.212)	205.993 (201.512 - 209.779)	2.114 (1.768 - 2.278)
CR7d_LR_c_1	Dynamic	1040.23 (998.582 - 1091.098)	201.921 (197.449 - 208.597)	1.969 (1.896 - 2.09)
CR7d_LRCR_c_1	Dynamic	1045.289 (1004.061 - 1095.314)	202.667 (197.413 - 206.297)	2.009 (1.939 - 2.1)
CR7d_LR_c_all	Dynamic	1039.008 (996.17 - 1091.213)	207.562 (202.457 - 211.716)	2.359 (2.161 - 2.767)
CR7d_LRCR_c_all	Dynamic	1057.966 (1004.95 - 1097.65)	205.315 (201.36 - 211.757)	2.446 (2.198 - 2.723)

Supplementary material 10 - Static models on test/train split 001

Additional results on the baseline test set 001 (corresponding to the first data split in the 100 train/test splits) using the "pooled" predictions (each landmark represents an independent observation) are presented in S-Figures 30, 31 and 32. To facilitate better visualization, survival and competing risk models with administrative censoring at day 30 (surv30d, surv30d_cens7, CR30d_LRCR_c_1, CR30d_LR_c_1, CR30d_LRCR_c_all, CR30d_LR_c_all) and competing risk models with administrative censoring at day 7 and logrank split statistic (CR7d_LR_c_1, CR7d_LR_c_all) have been excluded.

Supplementary material 11 - Dynamic models on test/train split 001

Additional results on the dynamic test set 001 (corresponding to the first data split in the 100 train/test splits) using the "pooled" predictions (each landmark represents an independent observation) are presented in S-Figures 33, 34 and 35. To facilitate better visualization, competing risk models using logrank split statistic (CR7d_LR_c_1, CR7d_LR_c_all) have been excluded.

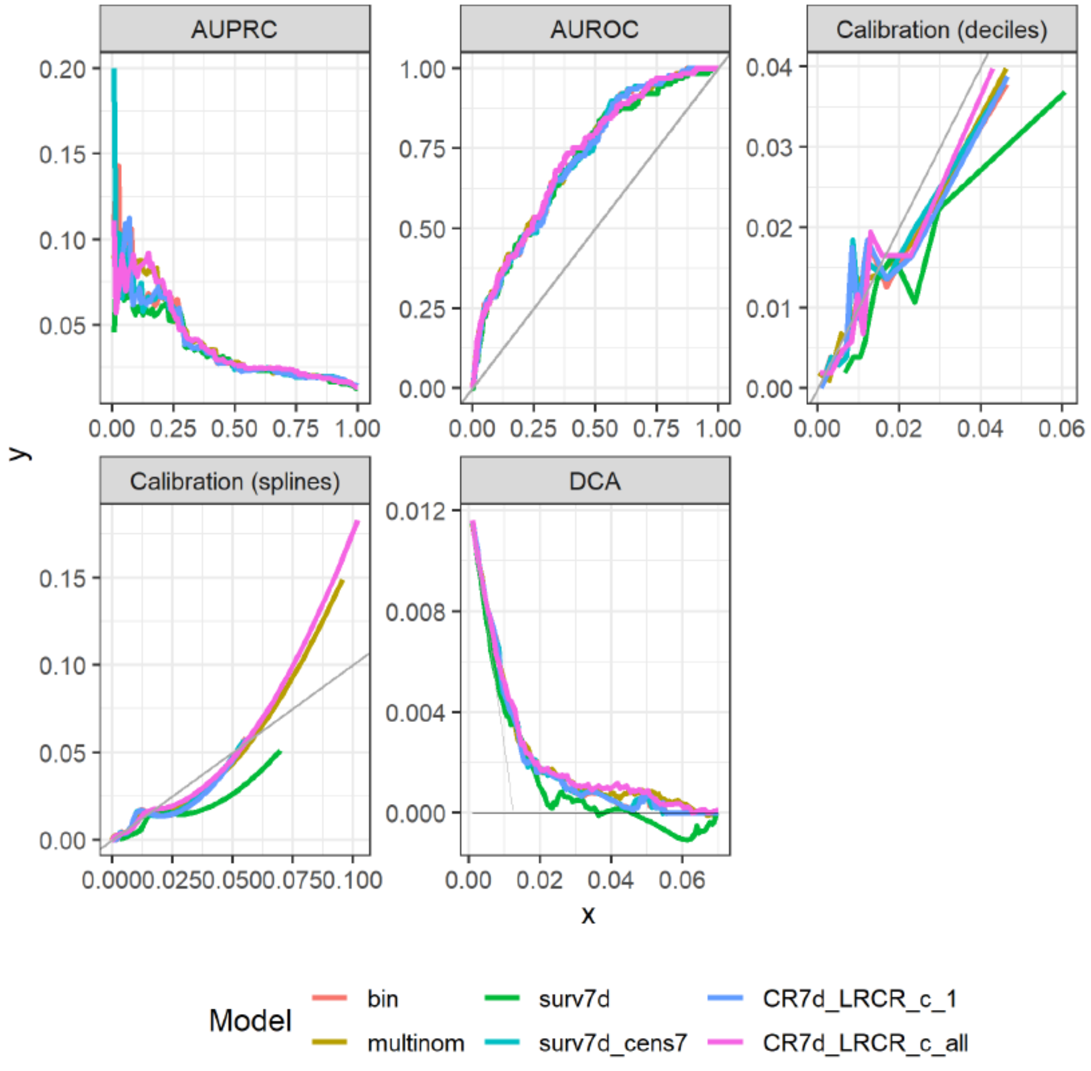

S-Figure 30: All curves. The x and y axis are: Recall and Precision for AUPRC; 1 - Specificity and Sensitivity for AUROC; Predicted probabilities and Observed probabilities for Calibration (deciles) and Calibration (splines); cutoff and Net benefit for DCA

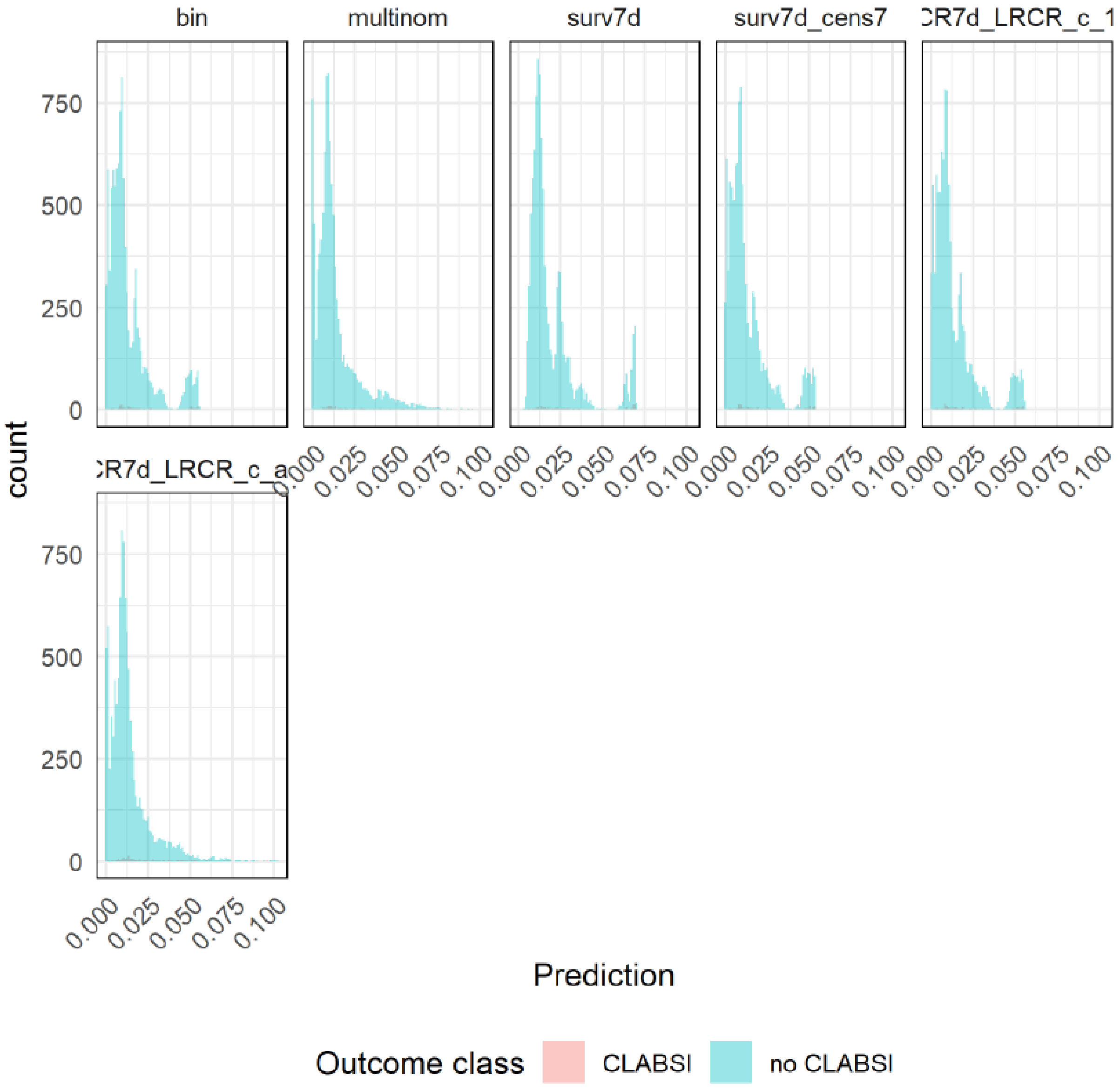

S-Figure 31: Predicted risks histogram (static)

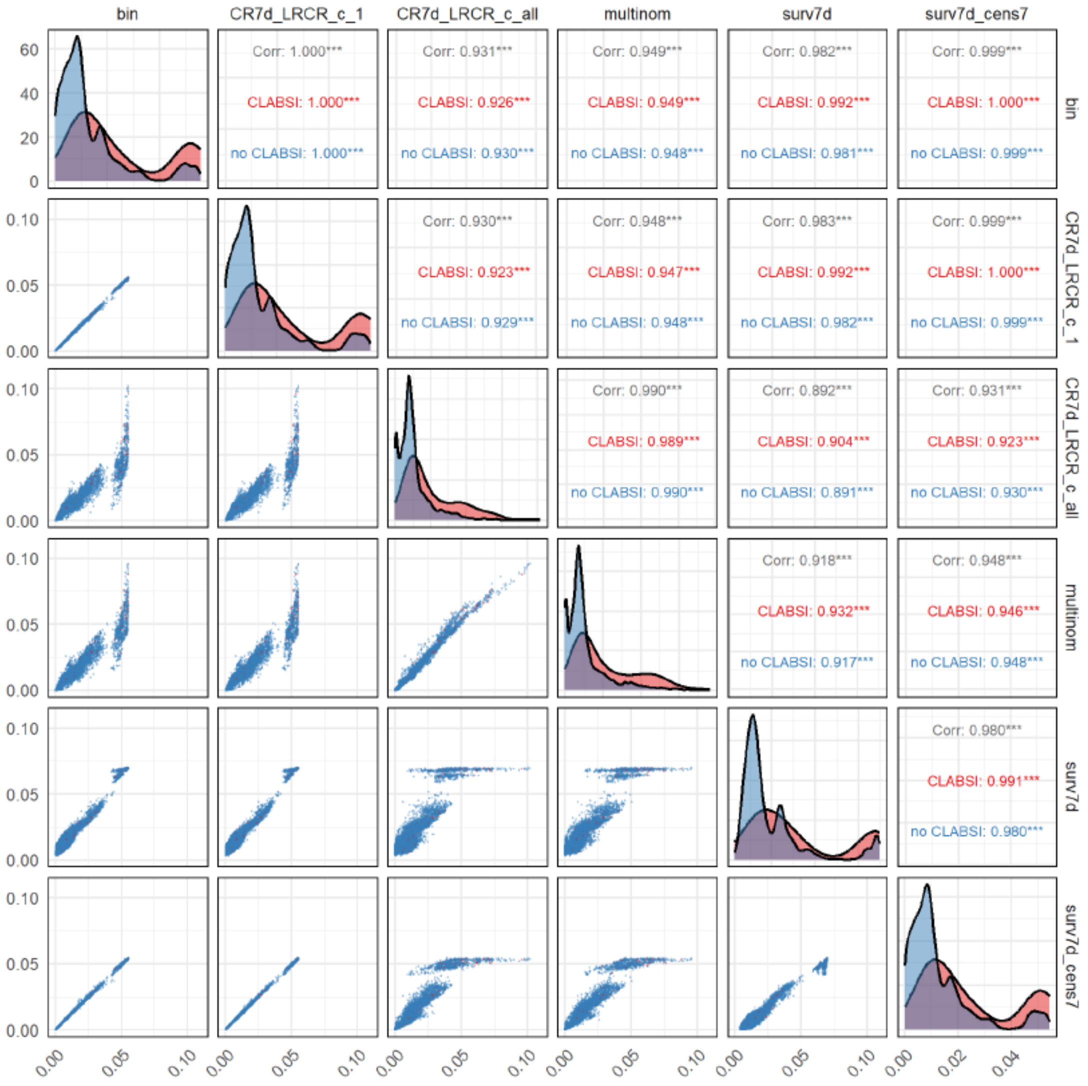

S-Figure 32: Comparison of predictions by model. The lower plots under the diagonal show the predictions of two models plotted against each other. The diagonal contains the the prediction density curves by class (CLABSI vs. no CLABSI). The upper plots above the diagonal show the correlation (Pearson correlation coefficient) for the predictions of the two models, as well as the correlation within in each class.

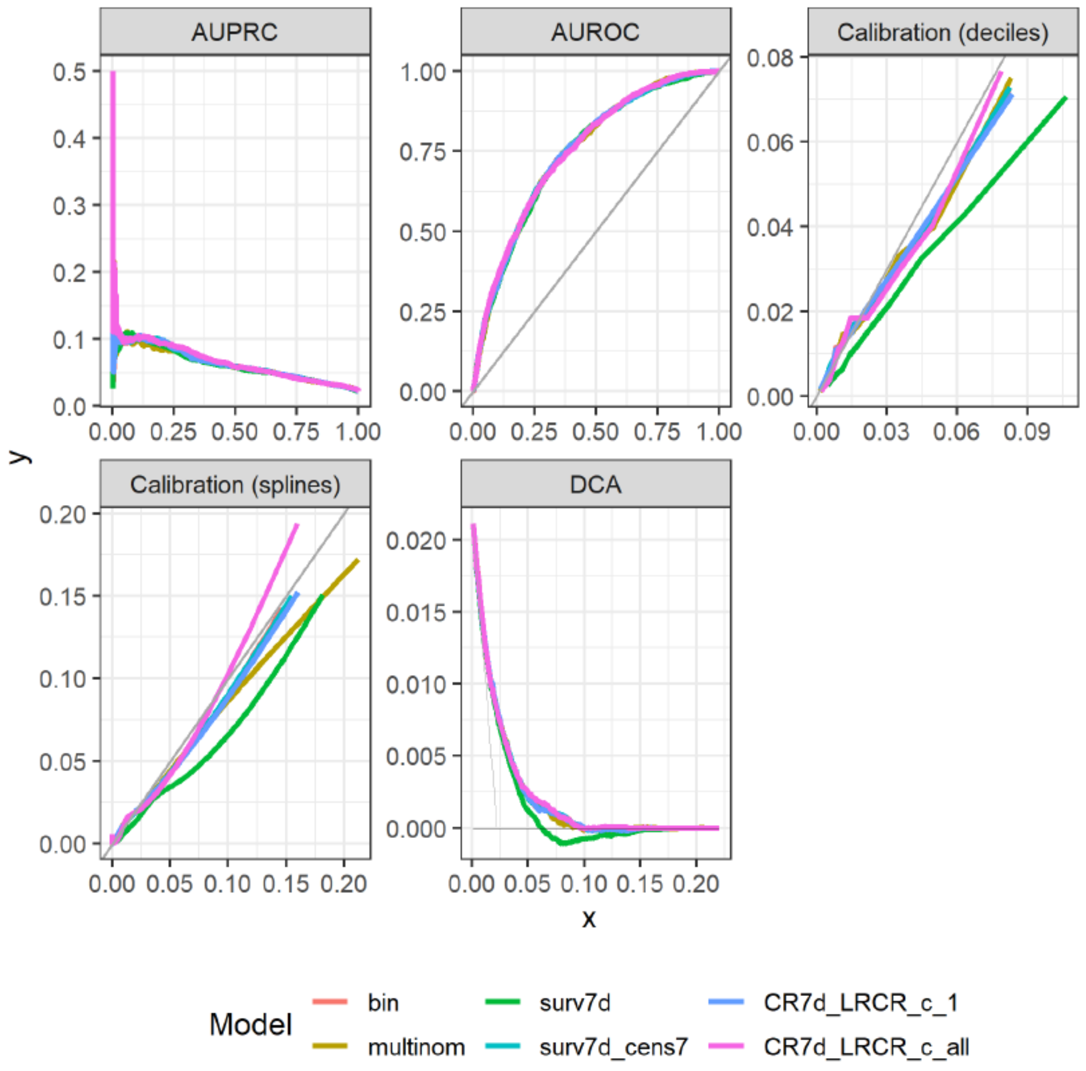

S-Figure 33: All curves. The x and y axis are: Recall and Precision for AUPRC; 1 - Specificity and Sensitivity for AUROC; Predicted probabilities and Observed probabilities for Calibration (deciles) and Calibration (splines); cutoff and Net benefit for DCA

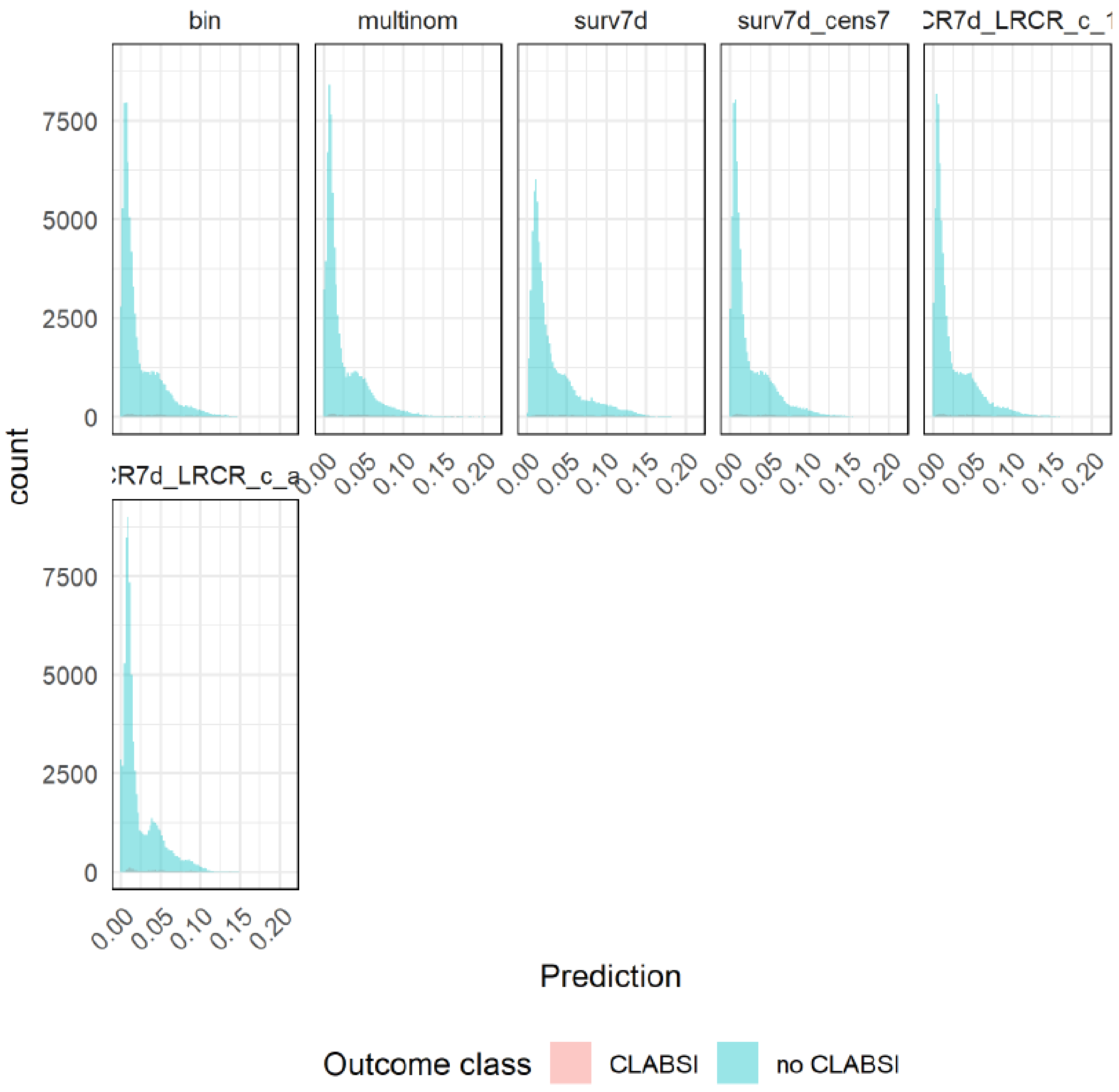

S-Figure 34: Predicted risks histogram (dynamic)

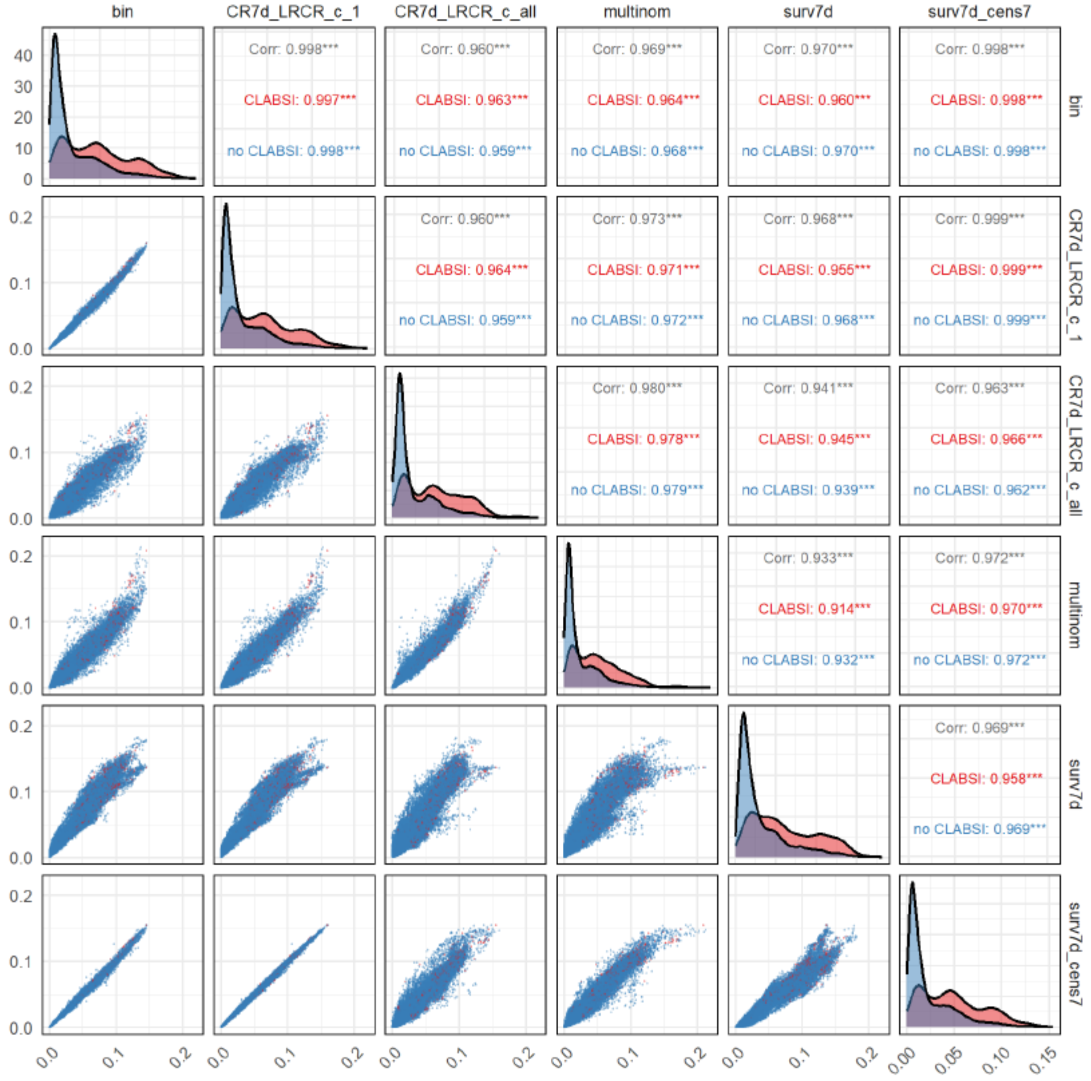

S-Figure 35: Comparison of predictions by model. The lower plots under the diagonal show the predictions of two models plotted against each other. The diagonal contains the the prediction density curves by class (CLABSI vs. no CLABSI). The upper plots above the diagonal show the correlation (Pearson correlation coefficient) for the predictions of the two models, as well as the correlation within in each class.

References

Albu, Elena. 2023. *missForestPredict: Missing Value Imputation Using Random Forest for Prediction Settings*. https://github.com/sibipx/missForestPredict.

Bischl, Bernd, Jakob Richter, Jakob Bossek, Daniel Horn, Janek Thomas, and Michel Lang. 2017. "mlrMBO: A Modular Framework for Model-Based Optimization of Expensive Black-Box Functions." *arXiv Preprint arXiv:1703.03373*.

Ishwaran, Hemant, Xi Chen, Andy J Minn, Min Lu, Michael S Lauer, and Udaya B Kogalur. 2021. "randomForestSRC: Minimal Depth Vignette." *Online Vignette*. https://www.randomforestsrc.org/articles/minidep.html.

Roustant, Olivier, David Ginsbourger, and Yves Deville. 2012. "DiceKriging, DiceOptim: Two r Packages for the Analysis of Computer Experiments by Kriging-Based Metamodeling and Optimization." *Journal of Statistical Software* 51: 1–55.

Vickers, AJ, B Van Calster, and EW Steyerberg. n.d. "A Simple, Step-by-Step Guide to Interpreting Decision Curve Analysis. Diagn Progn Res. 2019; 3: 18.

Vickers, Andrew J, and Elena B Elkin. 2006. "Decision Curve Analysis: A Novel Method for Evaluating Prediction Models." *Medical Decision Making* 26 (6): 565–74.

Wright, Marvin N, Stefan Wager, Philipp Probst, and Maintainer Marvin N Wright. 2019. "Package 'Ranger'." *Version 0.11* 2.
